# Promiscuous fungal peroxygenase from *Chaetomium globosum* by directed evolution

**DOI:** 10.1016/j.isci.2026.117534

**Published:** 2026-09-18

**Authors:** David M. Upp, Ivan Mateljak, Daniel Mendez-Sanchez, Patricia Gomez de Santos, Israel Sanchez-Moreno, Morgane Valles, Katarzyna Świderek, Vicent Moliner, Katrin Scheibner, Martin Hofrichter, David Gonzalez-Perez, Miguel Alcalde

**Affiliations:** 1 Department of Biocatalysis, Institute of Catalysis, CSIC, 28049 Madrid, Spain; 2 EvoEnzyme S.L., C/ Faraday 7, Parque Científico de Madrid, 28049 Madrid, Spain; 3 Department of Bio-organic Chemistry, Instituto de Química Orgánica General (IQOG), CSIC, C/ Juan de la Cierva, 3, 28006 Madrid, Spain; 4 BioComp Group, Institute of Advanced Materials (INAM), Universitat Jaume I, 12071 Castellon, Spain; 5 JenaBios GmbH, Löbstedter Str. 80, 07749 Jena, Germany; 6 Department of Bio- and Environmental Sciences TU Dresden, International Institute Zittau, Markt 23, 02763 Zittau, Germany

**Keywords:** Chaetomium globosum, peroxygenase, directed evolution, inverted enantioselectivity, anti, -Markovnikov oxidation, terpenes, isophorone, yeast

## Abstract

Fungal unspecific peroxygenases (UPOs) are appealing enzymes for C–H oxyfunctionalization reactions, and, among them, the short UPO type from *Chaetomium globosum* (*Cgl*UPO) is of special interest due to its diverse substrate scope and selectivity. Despite its significance, *Cgl*UPO shows low stability and poor heterologous expression which has hitherto impeded its use for practical applications. In this study, *Cgl*UPO was engineered by directed evolution to produce a highly active and stable secreted variant that performs selective oxidations on a panel of organic molecules, including ethylbenzene, styrene, 1,2,3,4-tetrahydronaphthalene (THN), isophorone, pinene, and limonene. Molecular dynamic simulations demonstrate how mutations F154L and M210V in the heme access channel are responsible for dramatic changes in the enzyme performance, including inverted enantioselectivities and anti-Markovnikov oxidation reactions.

## Highlights

• Directed evolution yielded a stable and highly expressed *Cgl*UPO variant in yeast
• The evolved enzyme efficiently oxyfunctionalizes diverse organic substrates
• *Cgl*UPO variant catalyzes anti-Markovnikov oxidations and inverted enantioselectivities
• Heme-channel mutations reshape substrate binding and catalytic outcome

## Graphical abstract

**Figure:**
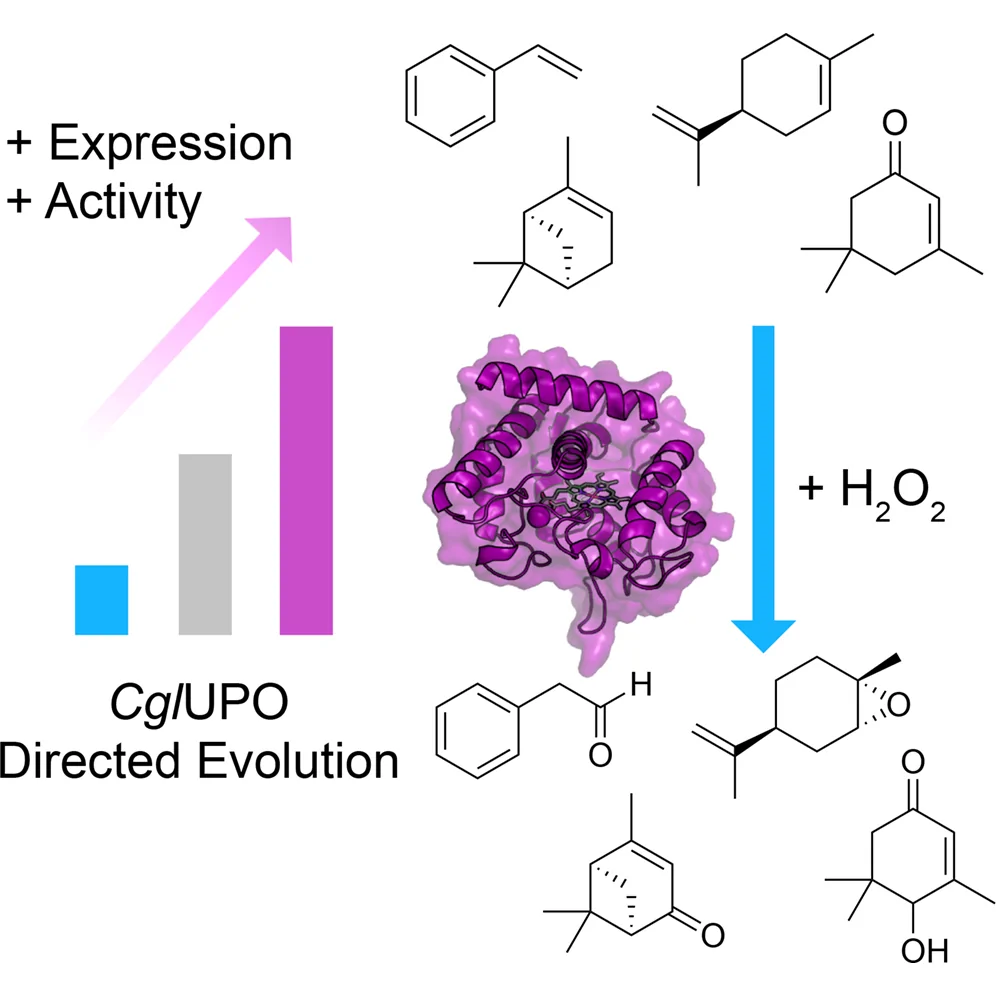

## Teaser

Biochemistry; Enzymology; Bioengineering; Biocatalysis; Biotechnology

## Introduction

Fungal unspecific peroxygenases (UPOs, EC 1.11.2.1) have proven to be a group of diverse and highly effective enzymes for C–H oxyfunctionalization reactions.^1^^,^^2^^,^^3^^,^^4^ Given their ability to selectively insert oxygen into organic molecules under mild conditions, UPOs offer a promising alternative to traditional chemical catalysts, aligning with the growing demand for sustainable and efficient processes in a variety of industrial applications, ranging from the production of fine and bulk chemicals to the synthesis of pharmaceuticals and their metabolites.^5^^,^^6^^,^^7^ In recent years, there has been an increasing demand for new UPOs that combine high selectivity (chemo-, enantio- and regio-), stability and the ability to be rapidly expressed and engineered in heterologous hosts to be able to meet industrial needs.

In 2017, a new UPO -*Cgl*UPO- was discovered in a ubiquitous cellulolytic and endophytic ascomycetous fungus—*Chaetomium globosum*—that colonizes a variety of materials in diverse environments including wallpapers. *Cgl*UPO was surprisingly divergent from the known UPO sequence space.^8^^,^^9^^,^^10^ This enzyme shows unique reactivity and selectivity among UPOs, with high activity against a variety of substrates including steroids (e.g., testosterone),^9^ resveratrol analogs,^11^ isophorone,^12^ norisoprenoids,^13^ long-chain unsaturated fatty acids,^14^ vegetable hydrolysates,^15^ and even short-chain terminal olefin fatty acids,^16^ among other compounds of chemical relevance. Despite the great biocatalytic potential of *Cgl*UPO, there is a common limitation in each of these reports: a lack of protein engineering of this enzyme. Indeed, in each of these studies, the expression of *Cgl*UPO was performed in the native host, *C. globosum*, which is a time-consuming process with limited production and which yields a homologous wild-type enzyme with poor stability. Significant drops in activity can be observed within days, even with storage at 4°C. An additional drawback is that protein engineering by directed evolution methodology cannot be applied in the native fungal host, limiting the capacity to improve its poor stability or modulate the activity and selectivity toward any reaction of interest.

Here, we have overcome these limitations by directed evolution of *Cgl*UPO focused on improving heterologous expression, stability, and activity in yeast. The outcome of this process was a recombinant *Cgl*UPO mutant that is easily secreted by *Saccharomyces cerevisiae* for future laboratory evolution studies and that can be overproduced in *Pichia pastoris* (*Komagataella phaffii*) in fed-batch bioreactor. The activity of variants produced in this campaign was characterized with a panel of substrates, unveiling striking characteristics including challenging anti-Markovnikov oxidations of terminal olefins, inverted enantioselectivities of aromatics, and increased overoxidation. To gain insight into these features, we employed atomistic computational modeling of the enzyme, with the aim of obtaining a mechanistic understanding that could guide the rational improvement of the performance of the newly designed variants.

## Results

### Directed evolution

A previous report described the functional expression of several UPOs, including *Cgl*UPO, in *S. cerevisiae* and *P. pastoris* by a modular shuffling system based on the Golden Gate cloning.^17^ While this study proved that the heterologous expression of *Cgl*UPO was possible, we found that the very low secretion levels in *S. cerevisiae* (∼0.6 mg/L) unfortunately precluded any further engineering. In the current study, we began by testing a variety of signal peptides originating from different peroxygenase species (*Marasmius rotula*, *Caldariomyces fumago*, *Agrocybe* *aegerita*—the native and evolved leader sequences^18^—and *C. globosum*), which were fused to mature *Cgl*UPO and evaluated under different culture conditions in order to foster protein secretion in a protease-deficient *S. cerevisiae* strain. The best result was obtained with the native *Cgl*UPO signal peptide, which allowed the enzyme to be secreted with total activity level of ∼3 U/L from microfermentations in 96-well plates (used for high-throughput screening [HTS] in directed evolution). This small but noticeable activity allowed this fusion (*Cgl*UPO_Sac_) to be used as the parent in a campaign of directed evolution to enhance its secretion in *S. cerevisiae* by iterative rounds of random mutagenesis, saturation mutagenesis, and *in vivo* DNA recombination (Figure 1).

**Figure 1:**
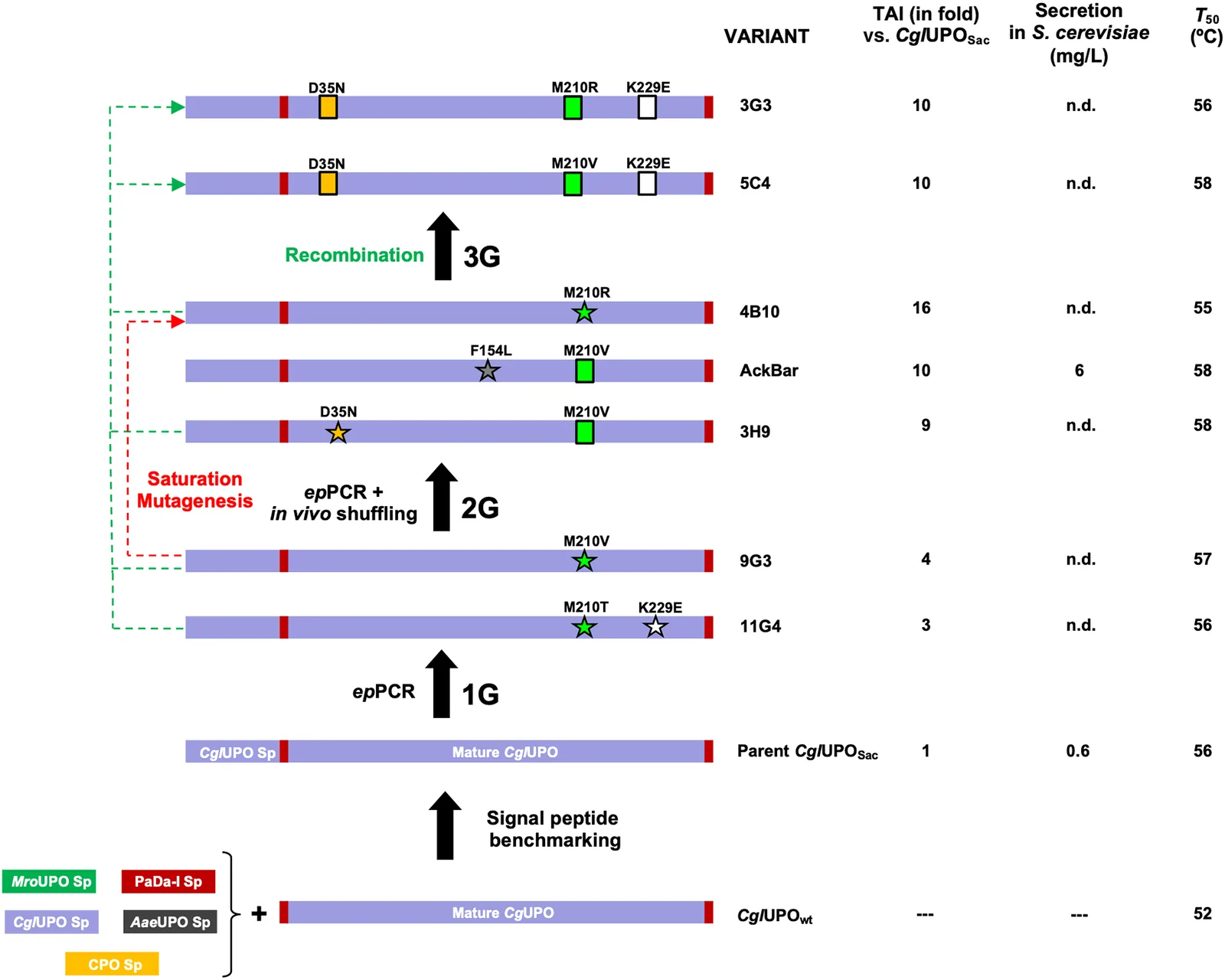
Directed evolution roadmap Different signal peptides were attached to mature *Cgl*UPO for secretion benchmarking: Signal peptides of UPO from *M. rotula* (in green), *C. globosum* (in purple), and *A.* (*Cyclocybe*) *aegerita* (in dark gray); evolved signal peptide of UPO from *A.aegerita* secretion mutant (PaDa-I, in red); and signal peptide of chloroperoxidase from *C. fumago* (in yellow). Three generations of random mutagenesis (by error-prone PCR, *ep*PCR), *in vivo* DNA shuffling, and site-saturation mutagenesis were carried out. Stars indicate the introduction of new mutations, which are represented as squares once they are integrated into the offspring of *Cgl*UPO genes. Red arrow indicates saturation mutagenesis at position 210. Green arrows indicate combination of mutations from first and second generations. Total activity improvement (TAI): The product of functional expression and DMP activity, measured from culture supernatants after second re-screening, presented as the average of triplicate measurements. *T*_50_ values were measured from crude extract supernatants from *S. cerevisiae* with the exception of *Cgl*UPO_wt_, which was homologously produced in *C. globosum*. n.d., not determined.

Due to the poor stability reported for the wild-type enzyme from the native fungus (*Cgl*UPO_wt_),^9^ the HTS assay was set up so that both total activity and thermostability could be monitored through the oxidation of the 2,6-dimethoxyphenol (DMP) into coerulignone (coefficient of variance of the assay ∼15%). It is worth noting that we observed an important kinetic thermostability increase of 4°C—from 52°C to 56°C—in the *T*_50_ (the temperature at which the enzyme keeps 50% of residual activity after incubation for 10 min) simply by switching the expression system of *Cgl*UPO from its native host, *C. globosum*, to *S. cerevisiae*. It is possible that this effect could be due to (hyper)glycosylation by yeast, which can be beneficial for stability (see biochemical characterization in Table 1). The first round of random mutagenesis led to two mutants with a significantly improved functional expression, the variant 9G3, which had the mutation M210V, and the variant 11G4, which carried two mutations, M210T and K229E (Figure 1). Given the presence of M210 T/V substitutions in these two mutants, a site-saturation mutagenesis library was constructed at position 210 leading to 4B10 variant, with a 16-fold total activity improvement due to the inclusion of the M210R mutation. In parallel, a new round of random mutagenesis in conjunction with *in vivo* DNA shuffling was carried out using 9G3 and 11G4 as parental types, generating the AckBar mutant (with the insertion of mutation F154L over 9G3) and the 3H9 mutant, with the incorporation of D35N substitution, and total activity improvements of 10- and 9-fold, respectively, providing secretion levels of ∼6 mg/L. In the final step of evolution, we sought to find an optimal combination of mutations among the different variants, but none of the resulting mutants improved the expression levels achieved in previous generations.

**Table 1:** Biochemical characterization of *Cgl*UPO_Pic_ and the AckBar mutant produced by *P. pastoris*

Biochemical feature	*Cgl*UPO_Pic_	AckBar
Mass (Da)^a^	37,000	37,000
Mass (Da)^b^	27,000	27,000
Mass (Da)^c^	27,560	27,494
Glycosylation degree (%)	24	24
Thermal stability (*T*_50_)	59.9	57.9
pH-activity profile	5.0–9.0	5.0–9.0
Production levels (mg L^−1^)	27 mg/L	90 mg/L
*K*_m_ (DMP, mM)	0.959 ± 0.200	0.014 ± 0.001
*k*_cat_ (DMP, s^−1^)	75 ± 5	68 ± 1
*k*_cat_/*K*_m_ (DMP, s^−1^mM^−1^)	78 ± 17	4,800 ± 400
*K*_i_ (DMP, mM)	45.4 ± 20	12.8 ± 0.8
*K*_m_ (veratryl alcohol, mM)	0.38 ± 0.02	1.28 ± 0.06
*k*_cat_ (veratryl alcohol, s^−1^)	6.14 ± 0.06	11.6 ± 0.1
*k*_cat_/*K*_m_ (veratryl alcohol, s^−1^mM^−1^)	15.9 ± 0.9	9.0 ± 0.4

a Estimated mass of glycosylated protein by SDS-PAGE.

b Estimated deglycosylated enzyme mass by SDS-PAGE (deglycosylation performed with PNGase F).

c Computed mass of protein using amino acid sequence (ProtParam). Kinetic parameters presented as the average of triplicate measurements with the standard deviation.

At this point, we decided to perform a preliminary analysis of the peroxygenase activity and selectivity of the different mutants with three model compounds (ethylbenzene, styrene, and 1,2,3,4-tetrahydronaphthalene [THN]), Table S1. While most of the variants catalyzed the two-electron oxidation (mono-peroxygenation) of these molecules with similar enantioselectivities, the AckBar mutant showed a dramatic inversion of enantioselectivity with THN, which was converted into the corresponding benzylic alcohol (*S*)-1,2,3,4-tetrahydronaphthalen-1-ol, with an enantiomeric excess (ee) of 86%. This was in strong contrast with the parental UPO that showed (*R*) ee values of 20% or 26%, depending on whether the enzyme was expressed by *C. globosum* (the original wild-type host, *Cgl*UPO_wt_) or by *S. cerevisiae* (*Cgl*UPO_Sac_). A further surprising result from this experiment was the observation of anti-Markovnikov Wacker-Tsuji oxidation of styrene to phenylacetaldehyde, a long-pursued synthetic reaction, very recently reported in other UPOs^19^ and cytochrome P450 monooxygenases,^20^ but that has not been described for *Cgl*UPO. In the light of these results and that the two mutations of AckBar mutant were located in the heme access channel (see STAR Methods), we decided to produce the AckBar mutant at a larger scale in order to study its biochemical features and reactivity pattern.

### Fed-batch bioreactor production in *P. pastoris* and biochemical characterization

*S. cerevisiae* is the host of choice for directed UPO evolution, given the availability of several episomal vectors as well as the existence of consolidated protocols for mutant library construction and screening, while *P. pastoris* (*Komagataella phaffii*) is commonly used for overproducing the evolved variants, constituting the so-called tandem-yeast expression system for UPO studies.^21^ Accordingly, the parental *Cgl*UPO and the AckBar mutant were cloned in *P. pastoris* and fermented in a 30-L fed-batch bioreactor achieving production levels for AckBar of ∼90 mg/L (which translates to several UPO grams in culture supernatants), tripling those of parental enzyme in the same host (*Cgl*UPO_Pic_), without any process engineering optimization. Both recombinant UPOs were purified, and their main biochemical properties were assessed. The molecular mass obtained by SDS-PAGE for the smallest UPO glycoform was 37,000 Da, with an estimated degree of glycosylation of ∼24% (Table 1; Figure S1).

Kinetic thermostability was measured by the determination of the *T*_50_ obtaining a value of ∼60°C which lies within the range of most stable recombinant UPOs expressed by yeast reported to date (Table 1; Figure S2).^5^ Unlike *Cgl*UPO_wt_, which is fully inactivated in a matter of days when stored at 4°C, the AckBar mutant keeps near 100% storage stability for months, becoming a suitable departure point for protein engineering and future industrial uses. The recombinant enzymes were active in the pH range from 5.0 to 9.0 (Table 1; Figure S3). In terms of kinetic parameters, a significant increase in DMP catalytic efficiency (Table 1; Figure S4) was observed with AckBar, a side effect of being the substrate used for screening, though an increase in substrate inhibition cooccurred. Oxidation of veratryl alcohol was largely unchanged, however, with both variants having a relatively similar catalytic efficiency.

### Anti-Markovnikov Wacker-Tsuji oxidation of terminal alkenes

With the completion of production, purification, and characterization of parental *Cgl*UPO_Pic_ and AckBar, an investigation into the oxidation of a series of compounds of industrial interest was performed. We began with one of the typical model substrates for peroxygenases, styrene (**1**), and observed the major product to be the expected styrene oxide (**1a**) (Table 2, entries 1 and 2).

**Table 2:** Epoxidation and Wacker-Tsuji anti-Markovnikov oxidation of styrenes by *Cgl*UPO_Pic_ and AckBar mutant


Entry	Variant	Styrene	TTN		Chemoselectivity
			Epoxide **a** (% ee (*S*))	Aldehyde **b**	Aldehyde:Epoxide ratio
1	*Cgl*UPO_Pic_	**1**	5,460 (52)	780	0.14
2	AckBar	**1**	5,580 (52)	820	0.15
3	*Cgl*UPO_Pic_	**2**	6,000 (43)	3,050	0.51
4	AckBar	**2**	3,390 (39)	2,690	0.79
5	*Cgl*UPO_Pic_	**3**	6,460 (ND)	1,730	0.27
6	AckBar	**3**	9,040 (ND)	1,200	0.13

Reactions were performed at 30°C with shaking at 750 rpm, with each reaction containing 2 mM substrate, 2 mM H_2_O_2_, 0.1 μM purified enzyme, and 5% acetone (v/v) in 50 mM phosphate, pH 6.0 (200 μL final volume). The reaction was stopped with the addition of 200 μL ethyl acetate containing 2 mM dodecane, which was extracted and filtered for analysis. TTN (μmol product/μmol enzyme) and enantioselectivity were determined by GC-MS with respect to a dodecane internal standard. Values shown here are the average of triplicate measurements.

As was the case with the *S. cerevisiae* expressed enzyme, high total turnover numbers (>5,000 TTN) with moderate levels of enantioselectivity [ee (*S*) 52%] were observed using low enzyme loadings of 0.1 μM. Wacker-Tsuji oxidation of terminal olefins with anti-Markovnikov chemoselectivity is highly desirable as these reactions provide direct access to aldehydes under mild conditions. Interestingly, both parental *Cgl*UPO_Pic_ and AckBar formed phenylacetaldehyde (**1b**), the anti-Markovnikov Wacker-Tsuji oxidation product, with TTN ∼ 800, but modest chemoselectivity (∼0.15 aldehyde:epoxide ratio). Two other styrenes were tested, 4-chlorostyrene (**2**) (entries 3 and 4) and α-methylstyrene (**3**) (entries 5 and 6), which showed both variants achieving generally higher ratios of aldehyde to epoxide. In particular, **2** was oxidized to aldehyde (**2b**) with TTN > 2,500 and with much higher chemoselectivity, 0.51 and 0.79 for *Cgl*UPO_Pic_ and AckBar, respectively. This is consistent with other UPO-catalyzed anti-Markovnikov Wacker oxidations,^19^ which suggests substrate electronic effects play a significant role in the observed selectivity. To ensure that the aldehyde product is formed directly from styrene by UPO catalysis and not from alternate pathways after epoxide formation (i.e., through a Meinwald-type rearrangement), reactions were carried out using styrene oxide as a substrate, both in the presence and absence of hydrogen peroxide. In both cases, no phenylacetaldehyde formation was observed (Figure S5), supporting the direct anti-Markovnikov oxidation from terminal alkenes by the UPOs without an epoxide intermediate.

As abovementioned, several recombinant UPOs expressed by *P. pastoris* have been recently reported to perform the anti-Markovnikov oxidation of terminal olefins.^19^^,^^22^ However, it is important to note that none of them were expressed in *S. cerevisiae* at the levels required to carry out a laboratory evolution campaign to improve their moderate chemoselectivity, which makes the AckBar mutant a promising departure point for engineering this activity toward the levels of selectivity necessary for industrial processes. To demonstrate this, we validated our *Cgl*UPO *S. cerevisiae* expression platform with an HTS assay based on an aldehyde-specific colorimetric compound, Purpald, which has previously been applied to the evolution of P450s for anti-Markovnikov oxidations.^20^ From microfermentations of *S. cerevisiae* containing the AckBar mutant in 96 well plates (i.e., clones grown in a final volume of 200 μL per well, with very poor oxygen availability and limited agitation), the assay provided a stable and quantifiable response against the formation of phenylacetaldehyde (Figures S6A and S6B) and was used to construct and screen a mutant library for anti-Markovnikov oxidations (Figure S6C). The mutational landscape was consistent with a low mutational loading highlighting the high levels of activity of AckBar secreted by *S. cerevisiae* in an HTS format, which paves the way for the future-directed evolution of this mutant with the aim of designing an efficient anti-Markovnikov-selective UPO.

### Investigation of other oxidations

We further explored the activity of our recombinant *Cgl*UPOs with a panel of different compounds including ethylbenzene (**4**), THN (**5**), isophorone (**6**), pinene (**7**), and limonene (**8**), which have been targeted for oxidations by UPOs previously.^23^^,^^24^^,^^25^ The oxidation of **4** to 1-phenylethanol (**4a**) and subsequent two-electron overoxidation to acetophenone (**4b**) were observed with modest turnovers, though AckBar catalyzed the overoxidation to a much greater extent than *Cgl*UPO_Pic_ (Figure 2A). Unexpectedly, enantioselectivity was considerably higher for AckBar (ee (*R*) 79%) compared to the *Cgl*UPO_Pic_ (ee (*R*) 31%). As seen during the preliminary characterization in *S. cerevisiae* with recombinant enzymes (Table S1), AckBar showed inverted enantioselectivity with **5**, producing **5a** (1-tetralol) with 85% ee for the (*S*)-enantiomer compared to *Cgl*UPO_Pic_ that achieved 39% ee for the (*R*)-enantiomer (Figure 2B). Once again, AckBar overoxidized to form **5b** (1-tetralone) to a much greater extent than the parental enzyme, but, even so, it yielded twice as much **5a** compared to *Cgl*UPO_Pic_. We also examined the oxidation of isophorone (**6**), a known *Cgl*UPO substrate of high industrial relevance. Oxidation at C4 of the α,β-unsaturated ketone was carried out with high regioselectivity and decent TTN (>1,200), in line with previous reports of *Cgl*UPO_wt_ activity,^12^ with higher rates of overoxidation observed for AckBar, though enantioselectivity was not analyzed (Figure 2C).

**Figure 2:**
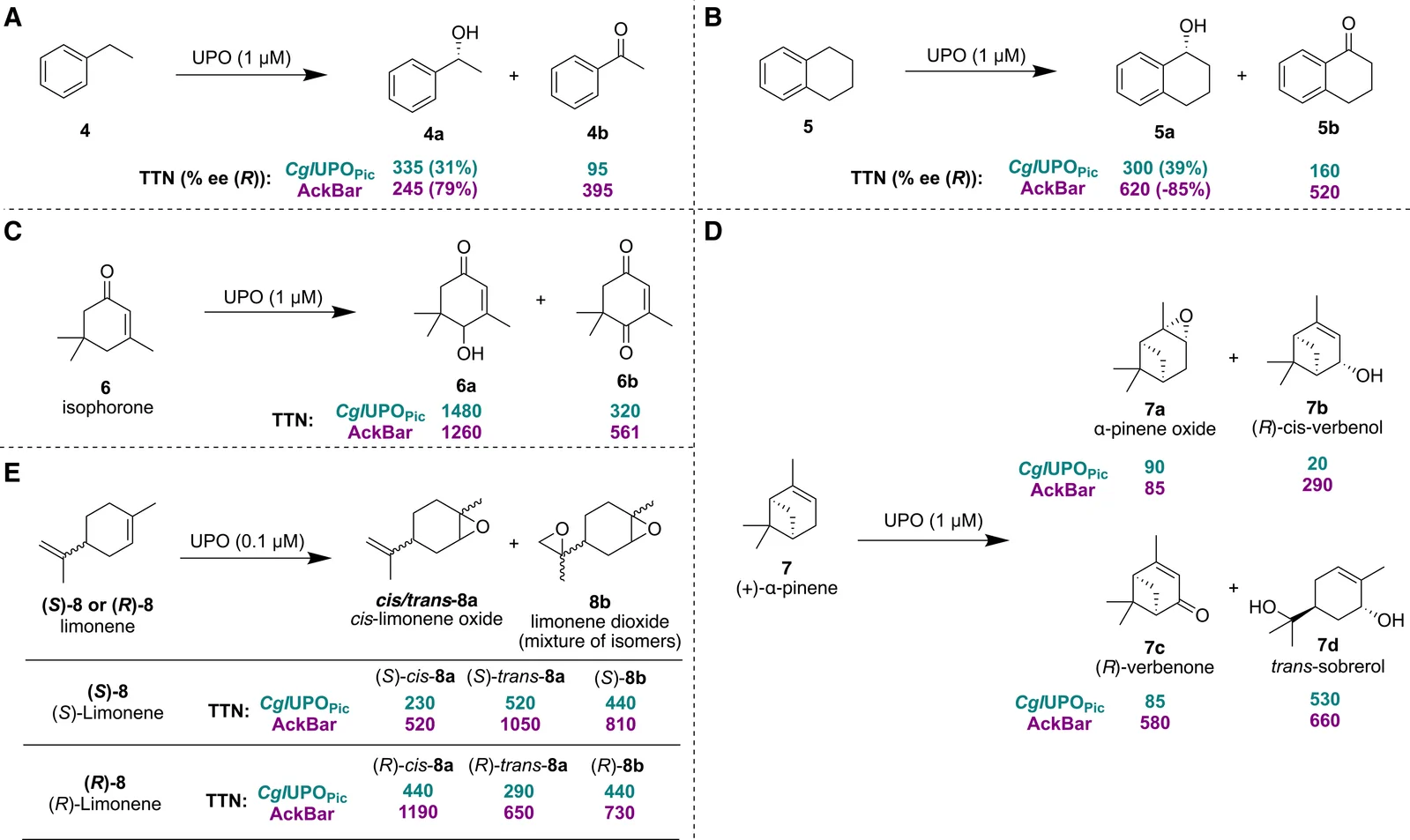
Oxidation reactions studied with *Cgl*UPO_Pic_ and AckBar mutant Ethylbenzene (A), tetrahydronaphthalene (B), isophorone (C), pinene (D), and (*S*)- and (*R*)-limonene (E). Reactions were carried out in 50 mM potassium phosphate pH 6.0 with 2 mM substrate, 2 mM H_2_O_2_, and 1 or 0.1 μM enzyme containing 5% acetone (v/v) (200 μL final volume). The reaction was stopped with the addition of 200 μL ethyl acetate containing 2 mM dodecane, which was extracted and filtered for analysis. TTN and enantioselectivity were determined by GC-MS with respect to a dodecane internal standard. Values shown here are the average of triplicate measurements. Enantioselectivity for the (*R*)-alcohols are shown in parentheses.

We also analyzed the oxidation of two prototypical terpenes, pinene and limonene (mono- and bicyclic monoterpenes) (Figures 2D and 2E). Concerning pinene (**7**), both *Cgl*UPO_Pic_ and AckBar produced considerable quantities of the epoxide **7a**, most of which underwent a spontaneous ring opening to the diol **7d**.^23^^,^^26^ While low levels ofC–H oxidation to verbenol (–**7b**) and verbenone (**7c**) were produced by *Cgl*UPO_Pic_, we were pleased to find that AckBar catalyzed considerably higher levels of C–H oxidation, with over 800 TTN between verbenol and verbenone. We hoped that similar C–H oxidation patterns would be observed in the oxidation of (*S*)-limonene (**(*S*)-8**) but unfortunately the *cis*- and *trans*-epoxide diastereomers (***cis*-8a** and ***trans*-8a**, respectively), along with the double epoxide diastereomers (8b), were the only products identified. Interestingly, no hydroxylation products, such as carveol or carvone, were observed in the reaction by either variant. We wondered if the specific limonene enantiomer was affecting the product distribution, so we also looked at the reaction of **(*R*)-8**. Rather than favoring ***trans*-8a** as was the case with **(*S*)-8**, both variants now favored the ***cis*-8a** with a ratio of approximately 2:1. Once again, however, no hydroxylation was observed, highlighting the clear preference for epoxidation over allylic C–H oxidation with this substrate, a finding that generally matches the oxidation of limonene by other UPOs.^22^^,^^23^

### Molecular dynamics studies

Long unbiased molecular dynamics (MD) simulations were conducted on the parental *Cgl*UPO and the AckBar variant to shed light on the observed opposite enantioselectivities in the oxidation of THN, as well as to reveal the origin of the observed anti-Markovnikov oxidation of styrene. Detailed descriptions of all system setups and computational methods used are provided in the supplemental information. Because no experimentally determined crystallographic structures are currently available, the initial structural model used for MD studies was generated with AlphaFold2^27^ and subsequently aligned using the DALI server^28^ with the crystal structure of the similarly folded unspecific peroxygenase from *Hypoxylon* sp. EC38 (*Hsp*UPO; PDB: 7O1R)^29^ (Figures S8 and S9).

#### Inverted enantioselectivity for THN oxidation

After initial preparation and equilibration of the non-covalent enzyme:substrate complex in *Cgl*UPO and AckBar using 0.1 μs of NPT (isothermal–isobaric ensemble) MD simulations, three independent 0.5 μs replicas were run for each system. As discussed earlier, AckBar showed a dramatic inverted enantioselectivity (i.e., the mutant and the parental enzyme had (*R*) ee values of −85% and 39%, respectively, Figure 2B). Simulations revealed that the substrate seems to remain firmly bound in the active site of both enzymes, despite slightly different poses, as depicted in Figure 3A. Interestingly, in both systems, the orientation of the substrate was found to be locked upon entry into the active site tunnel, where C8 (assignment based on orientation in the active site) was found to remain nearer to the Fe^IV^ = O1 group of compound I (Cpd I) with no flipping occurring (Figures S10–S12). This is supported by the population analysis, shown in Figure S13. For both enzymes, most structures exhibit a C8^THN^–O1^CpdI^ distance of ∼3.5 Å, while the C7^THN^–O1^CpdI^ distance is centered around ∼6 Å. In addition, as shown in Figure 3B, THN displays increased flexibility within parent *Cgl*UPO (see Figures S13 and S14 for further details) and no preference interaction for the two H atoms of C8:H7 or H8. In contrast, a preferential and more pronounced interaction between O1 and H8 is established in the case of the AckBar variant. Interestingly, a significant population of structures with THN in non-reactive poses, characterized by a C8^THN^–O1^CpdI^ distance centered around 7.5 Å, is observed in some of the replicas of *Cgl*UPO (Figure S14).

**Figure 3:**
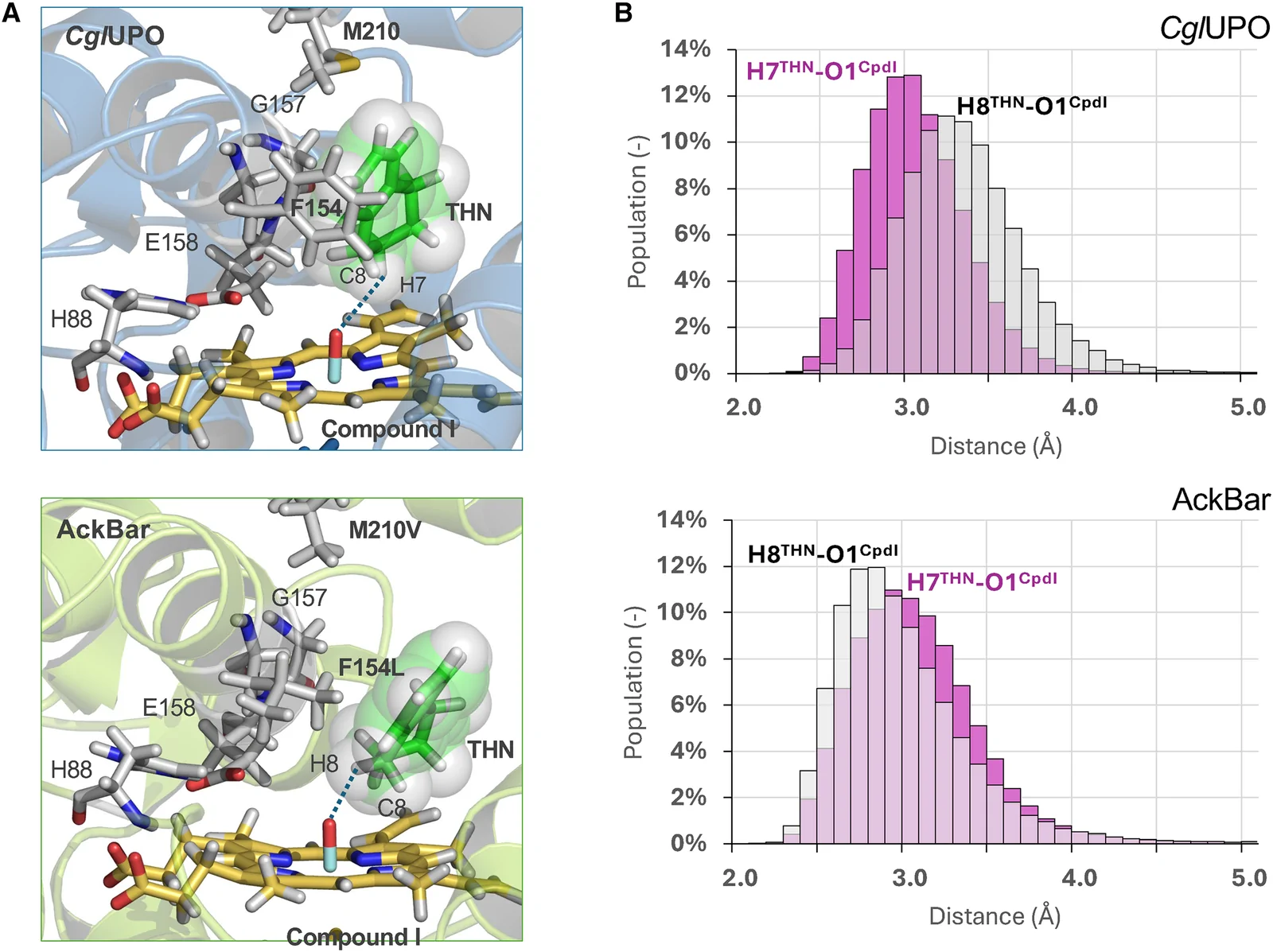
Computational analysis of the oxidation of THN (A) Detail of the representative structures of THN bound in the active site of *Cgl*UPO and AckBar mutant, compound I (Cpd I), derived from 0.5 μs of NPT MD. (B) Population analysis of the acceptor (O1^CpdI^)–hydrogen (H7^THN^ or H8^THN^ of C8) for THN bound in the active site of *Cgl*UPO and AckBar mutant. Data were obtained from 125,000 structures generated in five independent 0.5 μs NPT MD simulation replicas. AckBar mutations are highlighted in bold.

These findings suggest that the primary effect of the introduced F154L and M210V mutations in AckBar is to stabilize the substrate and maintain its position near the oxygen atom (O1) of Cpd I. AckBar meaningfully enhances the population of catalytically competent poses that would render a higher population of (*S*)-stereoisomers (defined by the threshold O1^CpdI^–Hx^THN^ distance <2.8 Å, shown in Figure S15). Despite the lack of perfect quantitative agreement, the computationally determined enantiomer ratios reproduce the same trend observed experimentally. The AckBar variant shows a significant shift toward enhanced formation of the (*S*)-enantiomer, while *Cgl*UPO favors the formation of the (*R*)-enantiomer.

Deeper structural analysis of THN positioning in the active sites of *Cgl*UPO and AckBar confirms notable differences (Figure 4). The *Cgl*UPO model suggests that the aromatic ring of THN is slightly tilted away from F154, leaving space between the substrate and the phenylalanine side chain. Although F154 provides a hydrophobic surface, it does not strongly restrict THN’s orientation. In contrast, the AckBar model simulations suggest that THN penetrates deeper into the active site, positioning its aromatic ring closer to L154. According to our simulations on the models, the AckBar pocket appears more open, as both mutations, F154L and M210V, enlarge the cavity and reduce steric constraints, allowing THN to adopt orientations that favor a single oxidation site. This deeper insertion also enables two stabilizing interactions with H88 and E158, which likely help maintain the substrate in a fixed position during MD simulations (Figure S16).

**Figure 4:**
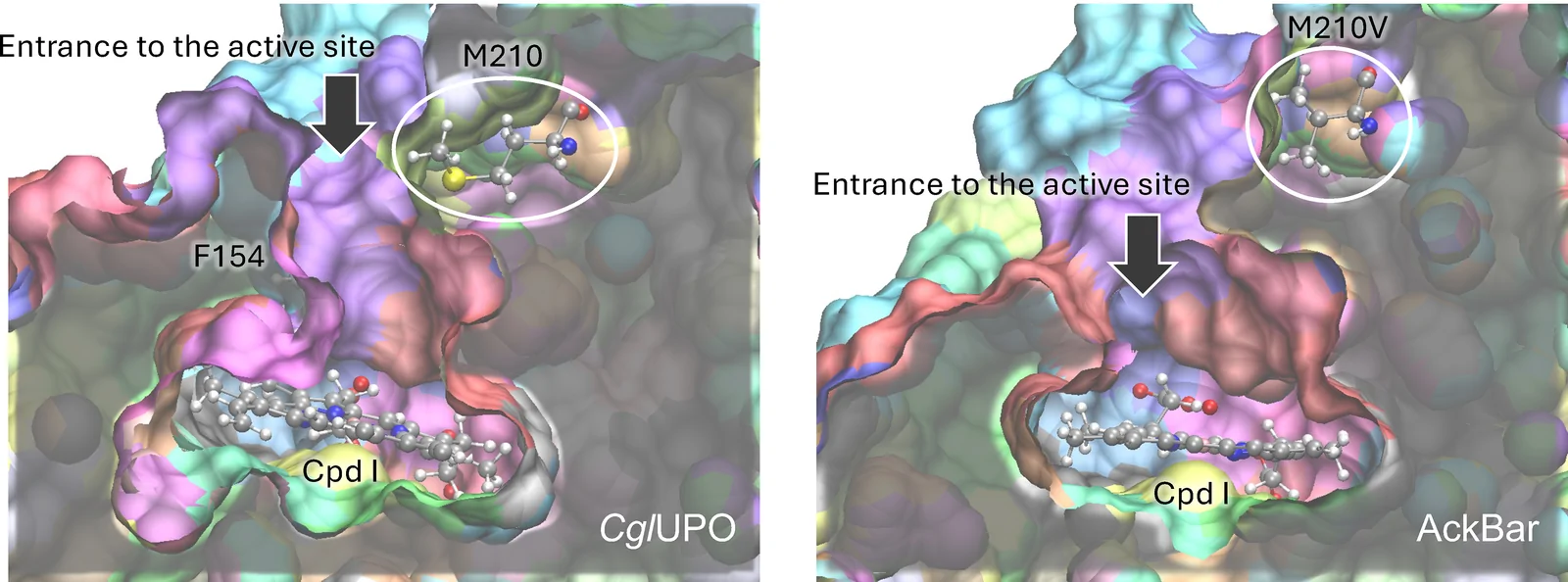
Comparison of the entrance to the active site of *Cgl*UPO and AckBar mutant Structures derived from 0.5 μs of NPT MD simulations. F154L mutation in AckBar, which is located behind the plane shown in the figure, is not displayed for clarity. The surface of the binding site is colored by the default color for the amino acid residue used in the VMD software.

#### Insights into the anti-Markovnikov Wacker oxidation

To elucidate the structural features of the AckBar variant that determine the epoxidation vs. anti-Markovnikov Wacker oxidation selectivity in the reaction with styrene, extensive MD simulations were performed with the styrene substrate positioned in the active site. While a radical cation intermediate has been proposed during the reaction pathway,^30^ we assume a concerted epoxidation pathway for this modeling study that matches other approaches.^19^^,^^20^^,^^31^ As illustrated in Figure 5, MD simulations suggest that the observed chemo- and enantioselectivity may depend on the orientation of the O1 atom of the Fe^IV^ = O moiety in Cpd I relative to the C7–C8 double bond of styrene. Specifically, an attack on the C7 carbon atom favors the epoxidation pathway (Table 2, **1a**), whereas a preferential attack on the C8 atom results in phenylacetaldehyde formation via an anti-Markovnikov Wacker oxidation mechanism (Table 2, **1b**). Furthermore, the stereochemistry of the resulting epoxide in the epoxidation pathway is determined by the relative positioning of the O1 atom with respect to the plane defined by the double bond and its substituents.^30^ In sum, our observations, shown in Figure 5, indicate that oxidation of styrene by AckBar predominantly yields the epoxide (∼80% of cases), with a slight preference for the (*S*)-enantiomer (ee (*S*) = 67%) consistent with experimental results (ee (*S*) = 52%, Table 2, entry 2). This descriptive analysis of the AckBar-styrene non-covalent complex improves our understanding of the factors that influence chemoselectivity and will be useful in future evolution campaigns.

**Figure 5:**
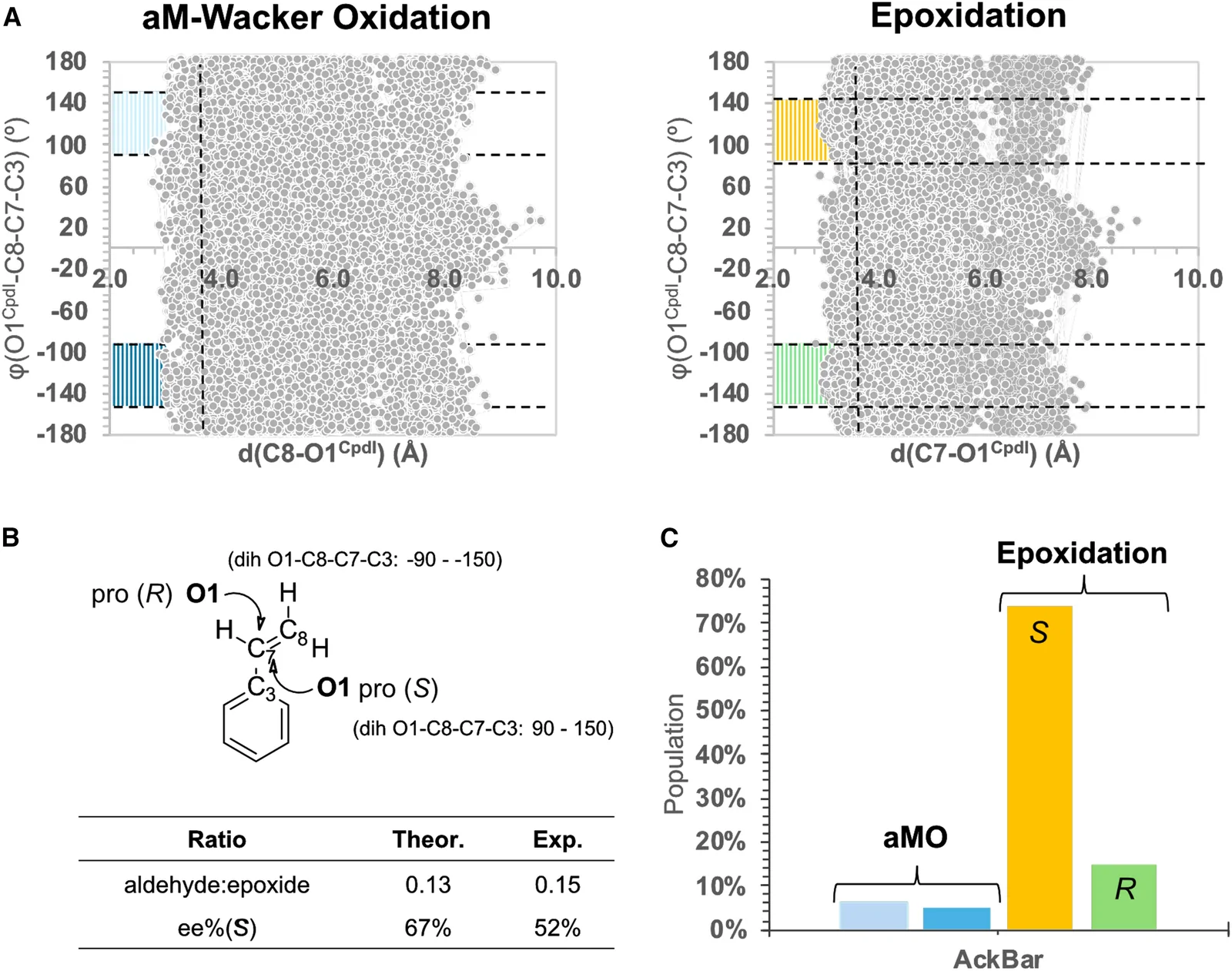
Epoxidation vs. anti-Markovnikov selective Wacker oxidation by AckBar mutant (A) Scatterplots of key geometrical parameters were extracted from the MD trajectories. The geometric analysis of AckBar-styrene non-covalent reactant complex was based on structures obtained from three independent 0.5 μs MD replicas. Frames exhibiting a shorter O1–C8 distance suggest an anti-Markovnikov selective Wacker oxidation pathway, while those with an O1–C7 distance below 3.8 Å are indicative of an epoxidation mechanism. Among these, reactive conformations can be further classified based on the O1–C8–C7–C3 dihedral angle: angles between 90° and 150° correspond to formation of the (*R*)-epoxide, while those between −90° and −150° lead to the (*S*)-enantiomer. (B) Scheme showing C7 and C8 on styrene and the reaction data at these positions. (C) Populations of the different positions shown in (A).

## Discussion

The results discussed here are an important step in making *Cgl*UPO, an enzyme that has received significant interest because of its unique activity profile among UPOs, a more practically obtained and evolvable enzyme. Using directed evolution, we designed a recombinant secretion variant (AckBar) that is highly active on a number of small organic substrates and that is also significantly more stable than the homologously produced enzyme. The ability to catalyze anti-Markovnikov selective Wacker oxidation of alkenes makes this enzyme an excellent starting point to evolve toward this activity (work ongoing). This reaction is of great industrial relevance,^32^ and our platform based on a tandem yeast expression system (*S. cerevisiae* for lab evolution and *P. pastoris* for scale-up production) provides a straightforward approach to face this challenge. It is worth noting that no mutations were introduced into the signal peptide during the evolution campaign described in this work, despite the entire fusion gene being subjected to random mutagenesis and recombination. However, this does not suggest that the secretion leader is unsuitable for engineering toward this objective. Indeed, during the evolution of AckBar for anti-Markovnikov oxidations, mutations were deliberately introduced into the leader sequence to enhance expression levels beyond the current limits, to align with the sensitivity of the screening assay.^33^ Another promising strategy to improve functional expression is the use of ancestral sequence reconstruction (ASR), as recently demonstrated by the generation of an ancestral *Cgl*UPO variant that, after rational engineering, exhibited high efficiency in steroid core oxyfunctionalization.^34^ In this context, our group has engineered several ancestral nodes from different UPO lineages to explore new-to-nature chemistries (work currently under evaluation).

Finally, the increased overoxidation of AckBar is relevant for many biotransformations, among them with isophorone, as the biocatalytic synthesis of overoxidized product 4-ketoisophorone (**6b**) requires cumbersome enzyme cascade reactions.^12^ The particular reaction profile with the monoterpenes limonene and pinene further highlights the capacity of *Cgl*UPO to complement known UPO catalysis. Though limonene epoxidation is the primary reaction generally observed here and in other recent UPO studies,^22^^,^^23^ bicyclic pinene was notably hydroxylated with decent (approximately 50%) regioselectivity. Theoretical modeling was then used to understand the effects of mutations on the reactivity profiles between the variants and found that the more open active site of AckBar allowed unique binding interactions to occur, altering activity and selectivity. We are confident that with this starting point, the full extent of this powerful enzyme will be able to be engineered in the near future.

### Limitations of the study

All biotransformations in this report were carried out on analytical scale, so performance will likely change under preparative scales. The enantioselectivity of several products was not analyzed due to the instability or inability to separate enantiomers using several chiral columns. Moreover, no experimentally determined structure of *Cgl*UPO is available, so every structural and mechanistic interpretation offered here relies on an AlphaFold2 model aligned to the *Hsp*UPO crystal structure. The C-terminal region of the model was predicted with low confidence and was equilibrated separately before use.

## Resource availability

### Lead contact

Requests for further information and resources should be directed to and will be fulfilled by the lead contact, Miguel Alcalde (malcalde@icp.csic.es).

### Materials availability

All materials generated in this study are available from the lead contact without restriction upon reasonable request.

### Data and code availability

• Data supporting this study are available in the manuscript or supplemental information. Experimental procedures are included in the supplemental information and include materials and methods, production of the UPOs used in this manuscript, gas chromatography-mass spectrometry (GC-MS) or liquid chromatography (LC) chromatograms of all reactions shown in this manuscript, and details on modeling studies.
• This paper does not report original code.
• As of now, no standard database for MD data exists (see Holm et al.^28^ for a discussion); reduced trajectories from MD simulations with an explicit solvent model have been deposited at Zenodo^35^ with https://doi.org/10.5281/zenodo.19047657. Additional data are available from the lead contact upon reasonable request.

## Acknowledgments

D.M.U. was funded through Juan de la Cierva Formación - FJC2021-047144-I MCIN/AEI/10.13039/501100011033 and through la Unión Europea NextGenerationEU/PRTR. D.G.-P. was funded through CAM Talento Modalidad I, ref. 2022-T1/BIO-23851. This work was supported by the I+D+I PID2025-171440OB-I00-DARING, PID2022-142074OBI00-RAINBOW, PID 2019-106166RB-100-OXYWAVE, and PID2024-160737OB-I00 and Spanish projects funded by MCIN/AEI/10.13039/501100011033/FEDER, EU. K.Ś. thanks the Spanish Ministerio de Ciencia, Innovación y Universidades, for the grant to encourage research consolidation (ref. CNS2024-154238). The authors also thank the staff of the Servei d’Informàtica of the Universitat Jaume I as well as the local computational resources funded by the Generalitat Valenciana─European Regional Development Fund (ref. IDIFEDER/2021/02).

## Author contributions

D.M.U., I.M., D.M.-S., P.G.d.S., I.S.-M., and M.V. performed experiments and analyzed data. K.Ś. and V.M. performed molecular dynamics simulations and relevant analysis. K.S. and M.H. provided native *Cgl*UPO (DNA sequence and purified protein). M.A. conceived the project and provided funding for execution. D.M.U., D.G.-P., and M.A. wrote the paper, which was reviewed by all the authors.

## Declaration of interests

A patent application (EP26382725.5) was filed on this work.

## STAR★Methods

### Key resources table

**Table:** 

REAGENT or RESOURCE	SOURCE	IDENTIFIER
**Bacterial and virus strains**		

*S. cerevisiae* BJ5465	ATCC 208289; LGCPromochem (Spain) Genotype: MATa ura3-52 trp1 leu2Δ1 his3Δ200 pep4:HIS3 prb1Δ1.6R can1 GAL	ATCC-208289
*P. pastoris* BG11	BioGrammatics PS 10011/ATUM (USA) Genotype: aox1-Δ1	PPS-9011
*E. coli* XL2-Blue	Agilent Technologies (USA) Genotype: recA1 endA1 gyrA96 thi-1 hsdR17 supE44 relA1 lac [F′ proAB lacIqZΔM15 Tn10 (Tetr) Amy Camr]	200150

**Chemicals, peptides, and recombinant proteins**		

*DL*-2-phenylpropionaldehyde	ThermoScientific (Spain)	130861000
(*S*)-cis-verbenol	ThermoScientific (Spain)	347330050
(1*S*)-cis-verbenone	ThermoScientific (Spain)	203630500
(−)-carveol (mixture of isomers)	ThermoScientific (Spain)	B20636
Acetophenone	ThermoScientific (Spain)	102410025
Phenylacetaldehyde	ThermoScientific (Spain)	A14263
Styrene	Merck Life Science (USA)	S4972
Styrene oxide	Merck Life Science (USA)	S5006
4-chlorostyrene	Merck Life Science (USA)	C71203
Ethylbenzene	Merck Life Science (USA)	E12508
Tetrahydronaphthalene	Merck Life Science (USA)	429325
1-Phenylethanol	Merck Life Science (USA)	P13800
Purpald (4-amino-3-hydrazino-5mercapto-1,2,4-triazole)	Merck Life Science (USA)	162892
(*S*)-(−)-Limonene	Merck Life Science (USA)	218367
(*R*)-(+)-Limonene	Merck Life Science (USA)	183164
(+)-Limonene oxide (mixture of isomers)	Merck Life Science (USA)	218324
(*S*)-(+)-Carvone	Merck Life Science (USA)	435759
(*S*)-(−)-Perillyl alcohol	Merck Life Science (USA)	218391
Myrtenol	Merck Life Science (USA)	W343900
Isophorone	Merck Life Science (USA)	I3509
3-Chloroperbenzoic acid	Merck Life Science (USA)	273031
*Alpha*-methylstyrene	Merck Life Science (USA)	M80903
*Alpha*-tetralone	Merck Life Science (USA)	T19003
*Alpha*-tetralol	Merck Life Science (USA)	122408
2-(4-chlorophenyloxirane)	Merck Life Science (USA)	534757
4-hydroxyisophorone	Enamine Ltd (Ukraine)	EN300-68596
(4-chlorophenyl)acetaldehyde	BLD Pharm (China)	BD28705
(*S*)-(−)-limonene oxide	Ambeed Inc (USA)	A1372474
*Alpha*-pinene oxide	TCI Europe (Switzerland)	P1362
DMP (2,6-dimethoxyphenol)	TCI Europe (Switzerland)	D0639
NBD (5-nitro-1,3-benzodioxole)	TCI Europe (Switzerland)	M0848
Veratryl Alcohol	Merck Life Science (USA)	D133000
H_2_O_2_ (30% w/w)	Merck Life Science (USA)	H1009
*Not*I	New England Biolabs (USA)	R0189
*Bgl*II	New England Biolabs (USA)	R0144
*Pme*I	New England Biolabs (USA)	R0560
*BamH*I	New England Biolabs (USA)	R0136
*Xho*I	New England Biolabs (USA)	R0146
Gibson Assembly Master Mix	New England Biolabs (USA)	E2611
iProof High-Fidelity DNA Polymerase	Bio-Rad (USA)	1725301
Yeast Transformation Kit	Merck Life Science (USA)	YEAST1
NucleoSpin plasmid kit	Macherey-Nagel (Germany)	740588.50
PCR Clean-up kit	Macherey-Nagel (Germany)	740609.50
Zymoprep yeast plasmid miniprep kit	Zymo Research (USA)	D2004

**Oligonucleotides**		

Primers (see Cloning section)	Integrated DNA Technologies (USA)	N/A

**Recombinant DNA**		

pJRoC30	California Institute of Technology (USA)	N/A
pBSY5Z	Bisy (Austria)	N/A

**Deposited data**		

DNA sequences	Supplemental information	N/A
GC/MS data	Supplemental information	N/A

**Software and algorithms**		

Prism 10	GraphPad (USA)	N/A
AlphaFold 2	Open source	N/A
Dali server	http://ekhidna2.biocenter.helsinki.fi/dali/	N/A
PROPKA 3.1	Schrodinger	N/A
AMBER (ff03.r1)	https://ambermd.org	N/A
BIOVIA Discovery Studio Visualizer	https://www.3ds.com/products/biovia/discovery-studio/visualization	N/A
NAMD 3.0	https://www.ks.uiuc.edu/Research/namd/	N/A
Zenodo MD deposit	https://doi.org/10.5281/zenodo.19047657	N/A

### Experimental model and study participant details

All yeast and bacterial species used in this study are listed (including origin and genotypes) in the key resources table.

### Method details

#### Culture media and expression

##### Synthetic complete (SC) drop-out minimal media

SC minimal medium for microplates and flasks contained 6.7 g/L filtered yeast nitrogen base, 1.92 g/L filtered yeast synthetic drop-out medium supplement without uracil, 2 g/L filtered raffinose pentahydrate and 25 mg/L filtered chloramphenicol. SC drop-out plates contained 6.7 g/L filtered yeast nitrogen base, 1.92 g/L filtered yeast synthetic drop-out medium supplement without uracil, 20 g/L autoclaved bacto-agar, 2 g/L filtered glucose and 25 mg/L filtered chloramphenicol.

##### *S. cerevisiae* expression media

Sterile expression medium contained 20 g/L bacto peptone, 10 g/L yeast extract, 67 mM KH_2_PO_4_ pH 6.0 buffer, 2.2% filtered galactose, 2.2 mM filtered MgSO_4_, 3.16% ethanol, and 25 mg/L filtered chloramphenicol. YP medium contained 10 g/L yeast extract, 20 g/L bacto peptone. YPD solution contained 10 g/L yeast extract, 20 g/L bacto peptone, 2 g/L sterile glucose, 25 mg/L chloramphenicol. Luria-Bertani (LB) medium contained 5 g/L yeast extract, 10 g/L bacto peptone, 10 g/L NaCl (5g NaCl for zeocin selection), 100 mg/L ampicillin or 25 mg/L zeocin.

##### Media for bioreactor expression in *P. pastoris*

Basal salts medium (BMG) with initial volume of 12 L contained 26.7 mL/L 85% phosphoric acid, 0.93 g/L CaSO_4_·2H_2_O, 14.9 g/L MgSO_4_·7H_2_O,18.2 g/L K_2_SO_4_, 4.13 g/L KOH, 40 g/L glycerol. The PTM_1_ Trace salts solution was sterilized by filtration and consisted of the following ingredients: 6 g/L CuSO_4_ 5H_2_O, NaI 0.08 g/L, 3 g/L MnSO_4_·H_2_O, 0.2 g/L Na_2_MoO_4_, 0.02 g/L H_3_BO_3_, 0.5 g/L CoCl_2_, 20 g/L ZnCl_2_, 65 g/L FeSO_4_·7H_2_O, 0.2 Biotin, 5 mL/L sulfuric acid.

#### Cloning of *Cgl*UPO in *S. cerevisiae*

The gene encoding for the main *Cgl*UPO (GenBank accession no.: CHGG_00319, NCBI reference sequence: XM_001219539.1) from *Chaetomium globosum* (Mycobank no.: 172545, strain CBS 148.51) was used to synthesize several fusion genes attaching the signal peptides of peroxygenases from *Marasmius rotula*,^36^ *Chaetomium globosum*,^9^ *Agrocybe* *aegerita* wildtype and evolved secretion leader (PaDa-I)^18^ and *Caldariomyces fumago*.^37^ *Cgl*UPO fusions were cloned under the control of the GAL1 promoter of the pJRoC30 expression shuttle vector, using *BamH*I and *Xho*I restriction enzymes. The linearized vector was loaded onto a preparative agarose gel and purified with the NucleoSpin Gel and PCR Clean-up kit. Purified DNA was quantified using an Infinite M200 plate reader equipped with a NanoQuant plate (TECAN, Switzerland). The corresponding gene (200 ng each) was mixed with the linearized plasmid (100 ng) and transformed into *S. cerevisiae* using the yeast transformation kit for promoting *in vivo* gene reassembly and cloning by IVOE.^38^

#### Laboratory evolution of *Cgl*UPO and variants

Each DNA fragment generated by PCR was gel purified with the NucleoSpin Gel and PCR Clean-up kit and quantified using an Infinite M200 plate reader equipped with a NanoQuant plate (TECAN, Switzerland). Then, 200 ng of mutant library (or gene fragment) was mixed with 100 ng of linearized plasmid and transformed into *S. cerevisiae* as described above. The yeast transformants were incubated for 72 h at 30 °C and then subjected to the high-throughput screening protocols described below. The details for the construction of the mutant libraries for each directed evolution round are as follows.

##### First round

*Cgl*UPO_Sac_ gene was subjected to *ep*PCR with *Taq* DNA polymerase in presence of MnCl_2_. Mutagenic PCR was prepared in a final volume of 50 μL containing 3% DMSO, 90 nM RMLN sense (5′-CCTCTATACTTTAACGTCAAGG) and RMLC antisense (5′- GGGAGGGCGTGAATGTAAGC), 0.3 mM dNTPs (0.075 mM each), 0.015 mM MnCl_2_, 0.1 ng/μL template, 1.5 mM MgCl_2_, and 0.05 U/μL of *Taq* DNA polymerase. PCR reaction was performed on a gradient thermocycler (Mycycler, BioRad, USA) using the following parameters: 95°C for 2 min (1 cycle); 94°C for 45 s, 50°C for 30 s, 72°C for 90 s (30 cycles) and 72°C for 10 min (1 cycle). Afterward, PCR products were purified and transformed into yeast as described above. Around 1,000 clones were screened for activity/secretion using the high-throughput screening for DMP described below.

##### Second round

This round of directed evolution was divided into two approaches; i) *ep*PCR of the whole gene and ii) site-saturation mutagenesis (SSM) at position 210. For the first approach, an *ep*PCR was performed using the best two winners of the previous round as DNA template (9G3 and 11G4) and the same conditions as in the first round of directed evolution. For the second approach, SSM on position 210, two PCR reactions were carried out to generate two fragments that contained engineering overlapping regions of 40–50 bp one each other and with the linear vector for *in vivo* recombination in yeast. The degenerated codon was located in the central region of the overlapping areas between fragments. The PCR reactions were performed in a final volume of 50 μL containing 3% DMSO, 0.3 mM dNTPs (0.075 mM each), 0.05 U/μL iProof DNA polymerase, and 2 ng/μL template, but each of them with different primers. For PCR 1, 0.25 μM of RMLN as forward primer and (5′–TATAATTTTGTATTAGCGC**MNN**AAACTTATCAAGGTCGTT) as reverse primer were used. For PCR 2, 0.25 μM of (5′–AACGACCTTGATAAGTTT**NNK**GCGCTAATACAAAATTATA) as forward primer and 0.25 μM of RMLC as reverse primer were used. Degenerate codons are highlighted in bold. PCR reactions were carried out on a gradient thermocycler using the following parameters: 95°C for 2 min (1 cycle); 95°C for 45 s, 50°C for 45 s, and 72°C for 60 s (28 cycles); and 72°C for 10 min (1 cycle). Both mutant libraries were cloned into *S. cerevisiae* as described above. Around 1,000 clones were screened against DMP activity, while for the SSM library 300 clones were screened using the HTS assay described below.

##### Third round of directed evolution

Mutations coming from 11G4, 9G3, 3H9 and 4B10 were combined and the corresponding genes were synthesized by IDT technologies (USA) and cloned into *S. cerevisiae* as described above.

##### Additional round of directed evolution for anti-Markovnikov oxidation activity

The AckBar gene was subjected to *ep*PCR using the same RMLN and RMLC primers with *Taq* DNA polymerase in presence of MnCl_2_ (0.015 mM) as described in the first round of directed evolution above. Then, 670 clones were screened using the high-througput screening for anti-Markovnikov oxidation method described below.

#### High-throughput screening assay (HTS)

Individual clones were picked and cultured in sterile 96-well plates containing 50 μL of SC minimal medium. In each plate, column 6 was inoculated with *Cgl*UPO_Sac_, or with the mutant winner of previous generation, as internal standard and well H1 was not inoculated (culture media control). Plates were sealed to prevent evaporation and incubated at 30 °C, 225 rpm, and 80% relative humidity in a humidity shaker (Minitron-INFORS, Biogen, Spain). After 48 h, 150 μL of expression medium were added to each well, followed by expression for an additional 48 h at 20 °C. The plates were centrifuged at 1,250 × *g* at 4 °C for 20 min (Centrifuge 5810R, Eppendorf, Germany). Aliquots of culture supernatants (20 μL) were transferred from the master plates to replica plates with the aid of a liquid handler robotic station Freedom EVO (Tecan, Switzerland). Then, 180 μL of a DMP reaction mixture (100 mM sodium phosphate buffer pH 6.0, 2 mM DMP and 2 mM H_2_O_2_) were added to plates using a multidispensing robot (Multidrop Combi Reagent Dispenser, Thermo Scientific, USA). The plates were briefly shaken and absorbance was measured at 469 nm for DMP oxidation (Ɛ_DMP_ = 27,500 M^−1^ cm^−1^) with a plate reader in kinetic mode (SPECTRAMax Plus 384, Molecular Devices, USA). The plasmids from positive wells were recovered with Zymoprep yeast plasmid miniprep kit I, following manufacturer's specifications. As the product of the Zymoprep was impure and the DNA concentration very low, the shuttle vectors were transformed into supercompetent *E. coli* XL2-Blue cells and plated onto LB-ampicillin plates. Single colonies were selected to inoculate 5 mL of LB-ampicillin medium and incubated overnight at 37 °C and 225 rpm (Minitron-INFORS, Biogen, Spain). The plasmids were extracted (NucleoSpin plasmid kit), sent for DNA sequencing (GATC Biotech-Eurofins, Luxembourg) and transformed into *S. cerevisiae* for flask production and re-screening.

##### Rescreening

A single colony from the *S. cerevisiae* clone containing the gene of interest was picked from an SC drop-out plate and inoculated in 10 mL of SC minimal medium in a 100 mL Erlenmeyer flask, and incubated for 48 h at 30 °C and 230 rpm (Minitron-INFORS, Biogen, Spain). An aliquot of cells was removed and used to inoculate fresh SC minimal medium (100 mL) in a 500 mL flask (OD_600_ = 0.25). Once the cells completed two growth phases (6–8 h, OD_600_ = 1), 900 mL of expression medium were added to the culture (OD_600_ of 0.1). After incubating for 72 h at 20°C and 230 rpm (maximal UPO activity), the cells were recovered by centrifugation at 2,000 × *g* at 4 °C (Centrifuge 5810R, Eppendorf, Germany). The supernatant was double-filtered (using both glass membrane (G-1, Glassco, India) and a nitrocellulose membrane of 0.45 μm pore size) and subjected to activity and thermostability (*T*_50_) assays as follows: Aliquots of the flask supernatants were transferred to two replica plates, i) 50 μL of crude extract to thermocycler plates (Multiply PCR plate, Sarstedt, Germany), and ii) 20 μL to bottom-flat 96-well plates (the initial activity plates, IA). Thermocycler plates were sealed with thermoresistant film (Deltalab, Spain) and incubated at different temperatures using a thermocycler (MyCycler, Biorad, USA) for 10 min. Afterward, thermocycler plates were chilled on ice for 10 min and further incubated for 5 min at room temperature, then they were spun down for 10 s in a PCR plate centrifuge (PCR Plate Centrifuge II, VWR International, USA). Next, 20 μL were transferred from thermocycler plate to new bottom-flat 96-well plates for testing residual activity (RA). Both, initial and residual activity were estimated by adding 180 μL of 100 mM sodium phosphate buffer pH 6.0 containing 2 mM DMP and 2 mM H_2_O_2_. Plates were shaken briefly and the absorption at 469 nm was followed in a plate reader in kinetic mode (SPECTRAMax Plus 384, Molecular Devices, USA). The values were normalized as a ratio between residual activities (RA) and initial activities (IA) values.

#### Anti-Markovnikov HTS assay

Plates containing *Cgl*UPO variants were prepared according to the microtiter plate expression method above. Fifty microliters of culture supernatants were transferred to new bottom-flat 96-well plates from master plates aided with a Freedom EVO liquid handler robotic station (Tecan, Switzerland). Next, 110 μL of potassium phosphate buffer pH 6.0, 20 μL of acetonitrile containing styrene, followed by 20 μL of H_2_O_2_ were added with the help of a multidispenser (Multidrop Combi Reagent Dispenser, Thermo Scientific, USA) to achieve a reaction mixture of 50 mM potassium phosphate buffer pH 6.0, 10 mM styrene, 5% acetonitrile and 2 mM H_2_O_2_. The reaction proceeded with shaking at 25 °C for 2 h, and then 50 μL of 50 mM purpald prepared in 2 M NaOH were added with a multichannel pipette. The color was allowed to develop for 30 min at room temperature, and the absorbance at 510 nm was recorded with a plate reader (SPECTRAMax Plus 384, Molecular Devices, USA).

#### Cloning, expression and purification in *P. pastoris*

##### *Cgl*UPO cloning in *P. pastoris*

*Cgl*UPO_Pic_ and AckBar variants were cloned into the vector pBSY5Z and produced using the carbon source repressed promoter PDF as previously described.^39^^,^^40^

##### Enzyme production in 30-L fed-batch bioreactor

*P. pastoris* clones containing *Cgl*UPO_Pic_ and AckBar were cultivated in a 30 L stainless steel bioreactor (F2, BIONET, Spain). After autoclaving the bioreactor with BMG media, 4.35 mL/L PTM_1_ trace salts were added. The pH was adjusted to 6.0 with ammonium hydroxide solution (28%) and was kept constant during cultivation. The fermentation process was started by adding 1.2 L of *P. pastoris* preculture grown on BMG medium in multiple 1,000 mL Erlenmeyer shaking flasks at 180 rpm and 30 °C for 18 h. The glycerol batch was run at 800 rpm and 25 °C. After complete consumption of glycerol (18–24 h), a methanol adaptation pulse of a final concentration of 0.5% (v/v) was conducted. After that, an exponential fed-batch phase on glycerol (50% (w/v)) with a specific growth rate of μ = 0.04 h^−1^ was performed at 25 °C until the end of fermentation (48 h). During whole fed-batch phase, methanol pulses to a final concentration of 1% (v/v) were supplemented while maintaining dissolved oxygen (DO) concentration above 30%. After the fed-batch medium was consumed or no increase of enzyme activity could be detected, the culture was harvested. The culture was clarified by centrifugation at 10,000 x *g* for 20 min at 4 °C. Subsequently, the supernatant was concentrated by ultrafiltration using by tangential ultrafiltration (Pellicon; Millipore, USA) through a 10 kDa-pore-size membrane (Millipore) by means of a peristaltic pump (Masterflex Easy Load; Cole-Parmer, Vernon Hills, USA).

#### Purification of *Cgl*UPO variants

*Cgl*UPO_Pic_ and AckBar produced in *P. pastoris* were subjected to anion exchange (AIX) and size-exclusion (SEC) chromatography using a ÄKTA pure FPLC (ÄKTA purifier, Cytiva, USA). The concentrated sample was filtered and dialyzed against 20 mM Bis–Tris buffer pH 7.0 (buffer A). The sample was loaded onto a strong AIX column (HiTraP QFF, GE healthcare, USA) pre-equilibrated with buffer A. The proteins were eluted with a linear gradient with buffer B (buffer A supplemented with 1 M NaCl) at a flow rate of 1 mL min^−1^: from 0 to 20% over 40 min and from 20 to 100% over 10 min. Fractions with *Cgl*UPO activity were combined, concentrated, and dialyzed against 50 mM potassium phosphate buffer pH 7.0 containing 150 mM NaCl. Purification by SEC was then carried out using a HiLoad 26/600 Superdex 200 pg column (GE Healthcare, USA) in a running buffer of 50 mM potassium phosphate at pH 7.0 containing 150 mM NaCl using a flow rate of 0.8 mL min^−1^. Fractions containing DMP activity were combined, concentrated and dialyzed back into Buffer A for final polishing purification. The sample was loaded onto a 10 μm high-resolution anion exchange Biosuite Q column (Waters, USA) pre-equilibrated with buffer A. The protein was eluted with a linear gradient with buffer B at a flow rate of 1 mL min^−1^: from 0 to 20% over 60 min and from 20 to 100% over 10 min. Concentration of the fractions containing DMP activity provided the purified enzyme used in all analyses. Purity was determined by SDS-PAGE (see Figure S1 for more details). Concentration of all purified proteins were determined using Bio-Rad Protein Assay Dye (Bio-Rad Laboratories, USA) using BSA as a standard.

#### Generic oxidation reaction method

Reactions were performed in 200 μL total volumes in microcentrifuge tubes at 2 mM substrate concentration with 0.1 or 1 μM purified enzyme in 50 mM potassium phosphate pH 6.0 buffer at 30 °C, 750 rpm (Thermomixer C, Eppendorf, Germany) with a reaction time of 1 h, in the presence of 2 mM H_2_O_2_. Prior to addition of hydrogen peroxide, substrates were dissolved in acetone and added to the reaction mixture to achieve a final acetone concentration of 5% (v/v). After the reaction was complete, 200 μL of ethyl acetate containing 2 mM dodecane (internal standard) was added and the mixture was vortexed for 10 s. The emulsion was centrifuged (Legend Micro 21R, Thermo Scientific, USA) at 10,000 x *g* for 1 min and the organic phase was removed by pipetting, filtered through a 0.20 μm syringe filter (Análisis Vínicos S.L, Spain) and analyzed by GC/MS (see below for more details). Peak areas in the GC/MS traces were calculated using automated peak detection methods. Total turnover numbers were calculated using calibration curves against the dodecane internal standard (see Calibration Curves section for more details).

#### GC chromatography

##### Achiral GC/MS for determination of TTNs

Chromatographic analyses were performed with a Shimadzu GC-MS QP2020 Ultra instrument, using a fused-silica Zebron-WAXPlus capillary column (30 m × 0.25 mm internal diameter, 0.25 μm film thickness) from Phenomenex (USA). A generic method was used for all achiral analyses: the oven was heated from 70 °C (1 min) to 240 °C (2 min) at 7 °C/min with a total flow of 13.2 mL/min. The injection volume was 2 μL with a split ratio of 1:10 and an injection temperature of 250 °C. Compounds were identified by comparison to authentic standards and mass fragmentation libraries (NIST).

##### Chiral GC analyses for determination of enantioselectivites

Chromatographic analyses were performed using the same instrument configured with a CP-Chirasil Dex CB capillary column (25 m × 0.32 mm internal diameter, 0.25 μm film thickness) from Agilent Technologies (USA). A generic method was used for all chiral analyses except tetrahydronaphthalene: the oven was heated from 50 °C (1 min) to 200 °C (2 min) at 6 °C/min with a total flow of 39.8 mL/min. The injection volume was 4 μL with a split ratio of 1:5 and an injection temperature of 250 °C. Compounds were identified by comparison to authentic standards and mass fragmentation libraries (NIST).

Tetrahydronaphthalene method: the oven was heated from 50 °C(1 min) to 145 °C (17 min) at 20 °C/min then to 200 °C (2 min) at 20 °C/min. The injection volume was 4 μL with a split ratio of 1:5 and an injection temperature of 250 °C.

#### Computational methods

##### System setup

The initial structural model of the *Cgl*UPO protein, based on its known amino acid sequence, was generated using the AlphaFold2 program,^27^ because of the lack of solved crystallographic 3D structures. This model (242 amino acids) was subsequently aligned using the DALI server^28^ with the crystal structure of a similarly folded unspecific peroxygenase from *Hypoxylon* sp. EC38, *Hsp*UPO (PDB ID: 7O1R, 261 amino acids),^29^ as shown in Figure S8. Despite sharing 46.24% sequence identity and 59.14% sequence similarity, as illustrated in Figure S9, *Cgl*UPO and *Hsp*UPO exhibit a nearly perfect structural overlay. The *Hsp*UPO structure was used to position the heme cofactor, along with one Mg^2+^ ion, in the active site of *Cgl*UPO. Further, this cofactor served as a template for the construction of the radical complex (+·Heme-Fe^4+^ = O), Compound I (Cpd I). Additionally, the imidazole (IMD) molecule present in the active site of the reference structure was used to guide the construction of tetrahydronaphthalene (THN). Missing force field parameters for the THN molecule were generated using General Amber Force Field (GAFF),^41^ and the partial atomic charges were computed at the AM1 method^42^ with bond charge corrections (AM1-BCC)^43^ using the Antechamber^44^ software. FF parameters for THN are provided in Table S2. The parameters for the radical complex, Cpd I, were adopted from the work of Cheatham III and co-workers.^45^ These parameters have already been validated for Cpd I through our previous computational simulations performed on the fungal peroxygenase *Cyclocybe (Agrocybe)* *aegerita* (*Aae*UPO) and its engineered Fett variants, demonstrating their reliability and effectiveness.^46^ Finally, parameters for the Mg^2+^ ion were obtained from the study by Villa and co-workers.^47^ The protonation states of ionizable amino acids were assigned based on pKa values from the empirical PROPKA 3.1 program^48^ (Figure S10; Table S3). In making these assignments, we considered the absence of disulfide bonds, the involvement of Cys17 in coordinating the Fe atom of Cpd I, and the role of Glu87 in coordinating the Mg^2+^ ion. All amino acids, except for glutamic acid, Glu158, and seven histidines, His31, His50, His83, His88, His99, His107, and His197, were found in their standard protonation states at pH 6.0, the condition used in the experiments. Regarding Glu158, according to the mechanism of Cpd I formation (Figure S11),^49^^,^^50^ this residue is proposed to operate as a base that abstracts a proton from hydrogen peroxide, afterward facilitating water departure. Consequently, and despite Propka predicting a pKa slightly higher than 6.0 (equal to 7.0) thus suggesting a population protonated state, the deprotonated state of Glu158 in the *Cgl*UPO model complexed with Cpd I was employed. All previously listed histidines were protonated at both Nδ and Nε positions of their imidazole rings. The remaining two histidines, with pK_a_ values lower than 6.0, i.e., His26 and His191, were found to be neutral and therefore protonated at one Nδ and Nε, respectively, based on geometrical inspection.

Despite the high per-residue confidence scores (Local Distance Difference Test, pLDDT >90)^51^ predicted for most amino acids in the generated *Cgl*UPO model (Figure S8), indicating strong accuracy in both backbone and side-chain positioning, a region spanning residues 222–242, corresponding to the C-terminus, exhibited reduced reliability. In this segment, pLDDT scores ranged from 50 to 70 (low confidence) and dropped below 50 (very low confidence), suggesting limited structural accuracy in that part of the model. Therefore, *prior to* proper simulations, the fragment spanning residues 222–242 was equilibrated during 10 ns of NVT molecular dynamic (MD) simulations at 303 K employing the Generalized Born implicit solvent (GBIS)^52^ model of water (with dielectric constant of 78.5) and the NAMD (ver. 2.0)^53^ engine. The AMBER ff03.r1^54^ classical force field was used to treat the protein. During this equilibration step, only the regions with low and very low pLDDT confidence scores were allowed to move. As shown in Figure S12, the time evolution of the RMSD for the simulated fragment indicates that the region reached equilibrium within this time frame. Consequently, the final structure from this simulation was used for subsequent studies.

Based on the final *Cgl*UPO conformation, a new AckBar variant of protein was constructed with BIOVIA Discovery Studio Visualizer (DSV, v21.1.0.20298, Dassault Systèmes, 2024) by exchanging phenylalanine to leucine in position 154 (F154L), and methionine to valine in position 210 (M210V) located in the heme access channel of the enzyme. Both models, complexed with Cpd I and the THN substrate, were subjected to MD simulations (see details in the following section). After 100 ns of initial preliminary equilibration, the final structure of AckBar was used to prepare a model with styrene (STR) as a substrate. Missing FF parameters for STR were generated as described for THN, and are provided in Table S4. Hydrogen atoms were added to all systems using the tLEAP module of AmberTools program.^55^ All models were subsequently neutralized by adding six sodium ions at electrostatically most favorable positions and solvated in a water box with 10 Å set as the buffering distance between the edges of the box and the protein. The parameters for sodium ions and water molecules were adapted from the TIP3P^56^ FF.

##### MD simulations

After energy minimization using 10^5^ steps of conjugate-gradient minimization, systems were gradually heated to 303 K with 0.1 K increments and underwent 500 ps of NPT equilibration. The last snapshot obtained after the equilibration step was then used as an initial geometry for three independent MD simulations for each model, with the temperature being controlled using the Langevin thermostat^57^ and the pressure with the Nosé-Hoover Langevin piston.^58^ Periodic boundary conditions (PBC) were used, and the non-bonded interactions were treated with a smooth switching function applied between 14.5 and 16 Å using Particle-Mesh Ewald (PME) summation. In all cases, classical MD simulations were conducted using NAMD (ver. 3.0),^59^ and a 2 fs time step was used through the SHAKE algorithm.^60^ Time evolution of the root-mean-square deviation (RMSD) computed for the backbone atoms (C, O, Cα, and N) of the protein and for the substrate heavy atoms was analyzed to confirm the stability of the reactant-complex structures, as shown in Figure S13. All data processing and analysis were done using cpptraj package.^61^

### Quantification and statistical analysis

There are no quantification or statistical analyses to include in this study.
