# Supplementary material for: Promiscuous fungal peroxygenase from Chaetomium globosum by directed evolution
Source: iScience. 2026 Sep 18;29(10):117534. doi: 10.1016/j.isci.2026.117534 (PMC13663891; doi:10.1016/j.isci.2026.117534)
Supplement: Supplementary file 1 [file mmc1.pdf]

iScience, Volume *29*

Supplemental information

Promiscuous fungal peroxygenase

from *Chaetomium globosum* by directed evolution

David M. Upp, Ivan Mateljak, Daniel Mendez-Sanchez, Patricia Gomez de Santos, Israel Sanchez-Moreno, Morgane Valles, Katarzyna Świderek, Vicent Moliner, Katrin Scheibner, Martin Hofrichter, David Gonzalez-Perez, and Miguel Alcalde

SUPPLEMENTAL FIGURES

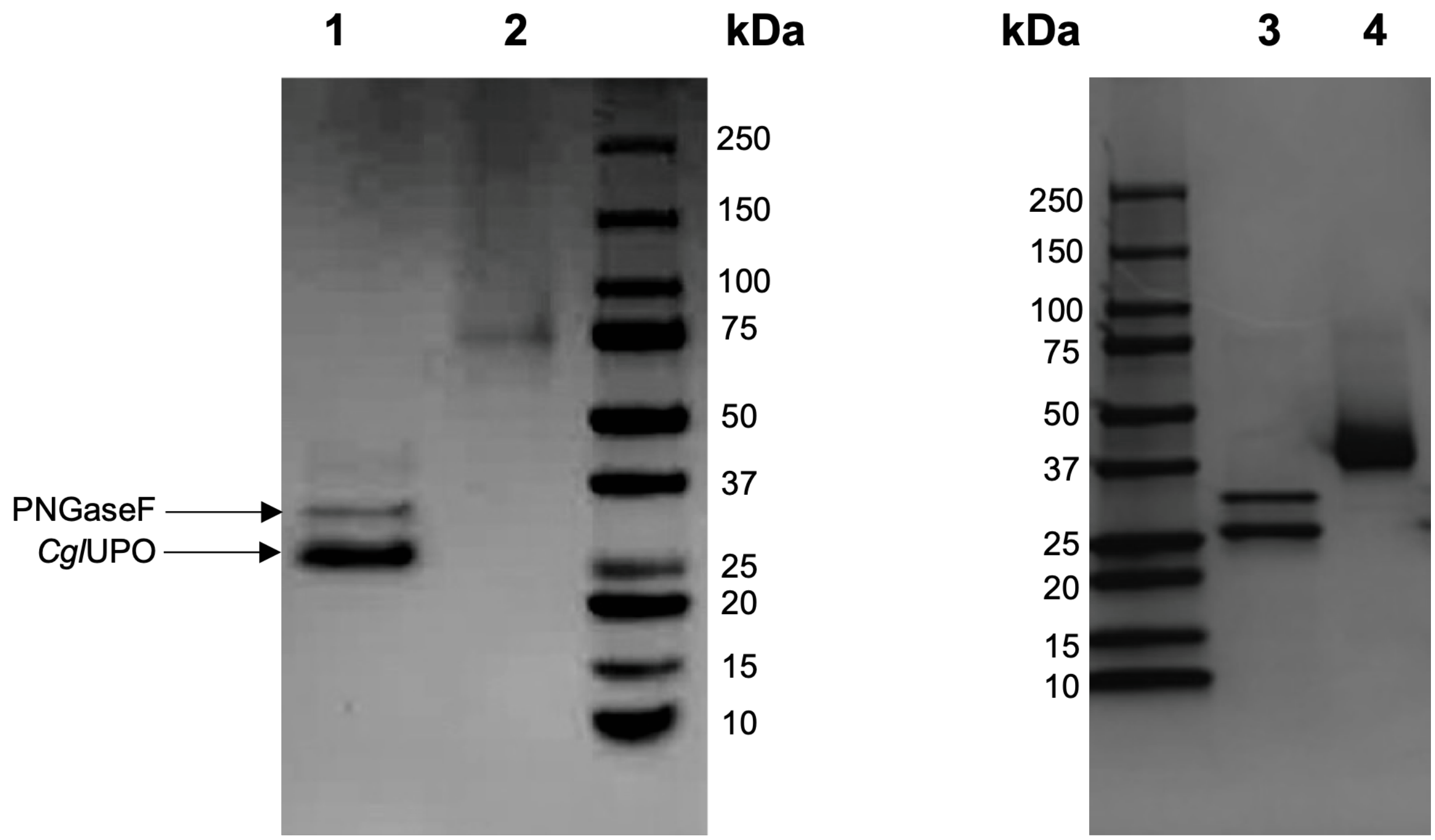

Figure S1. SDS-PAGE of recombinant *Cgl*UPO$_{Sac}$ and *Cgl*UPO$_{Pic}$ resolved on 4-20% precast polyacrylamide gels. Recombinant *Cgl*UPO$_{Sac}$ (left) after (1) and before (2) treatment with PNGaseF. Recombinant *Cgl*UPO$_{Pic}$ (right) after (3) and before (4) treatment with PNGaseF resolved on 4-20% precast polyacrylamide gels. The enzyme produced in *S. cerevisiae* (2) showed a much stronger glycosylation degree than in *P. pastoris* (4).

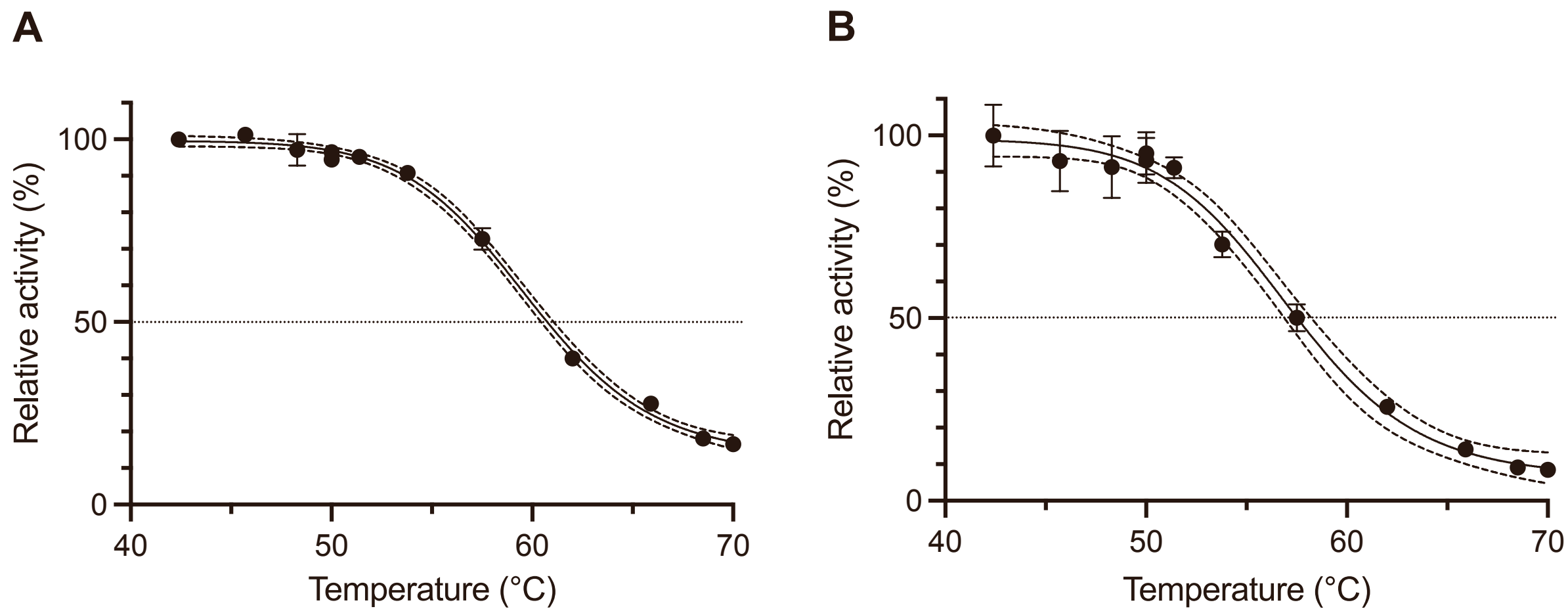

Sigmoidal curve
R^2: 0.984
T_{50} (°C): 59.9 ± .3

Sigmoidal curve
R^2 : 0.951
T_{50} (°C): 57.9 ± 1.5

Figure S2. Kinetic thermostability (T_{50}) of *Cgl*UPO$_{Pic}$ (**A**) and AckBar mutant (**B**). Kinetic thermostability was estimated by assessing the T_{50} values of the enzymes using 96-well gradient thermocyclers. Appropriate dilutions of purified enzymes were prepared in such a way that 20 µL aliquots gave rise to a linear response in the kinetic mode. Then, 50 µL were incubated for each point in a temperature gradient ranging from 42 to 70 ºC. After a 10 min incubation, samples were chilled out on ice for 10 min and further incubated at room temperature for 5 min. Afterwards, 20 µL of samples were assayed in 100 mM phosphate buffer pH 6.0, containing 2 mM H_2O_2 and 2 mM DMP. Substrate oxidations were followed through spectrophotometric changes at 469 nm. The thermostability values were deduced from the ratio between the residual activities and the initial activity. Each point represents the mean and standard deviation from 3 independent experiments.

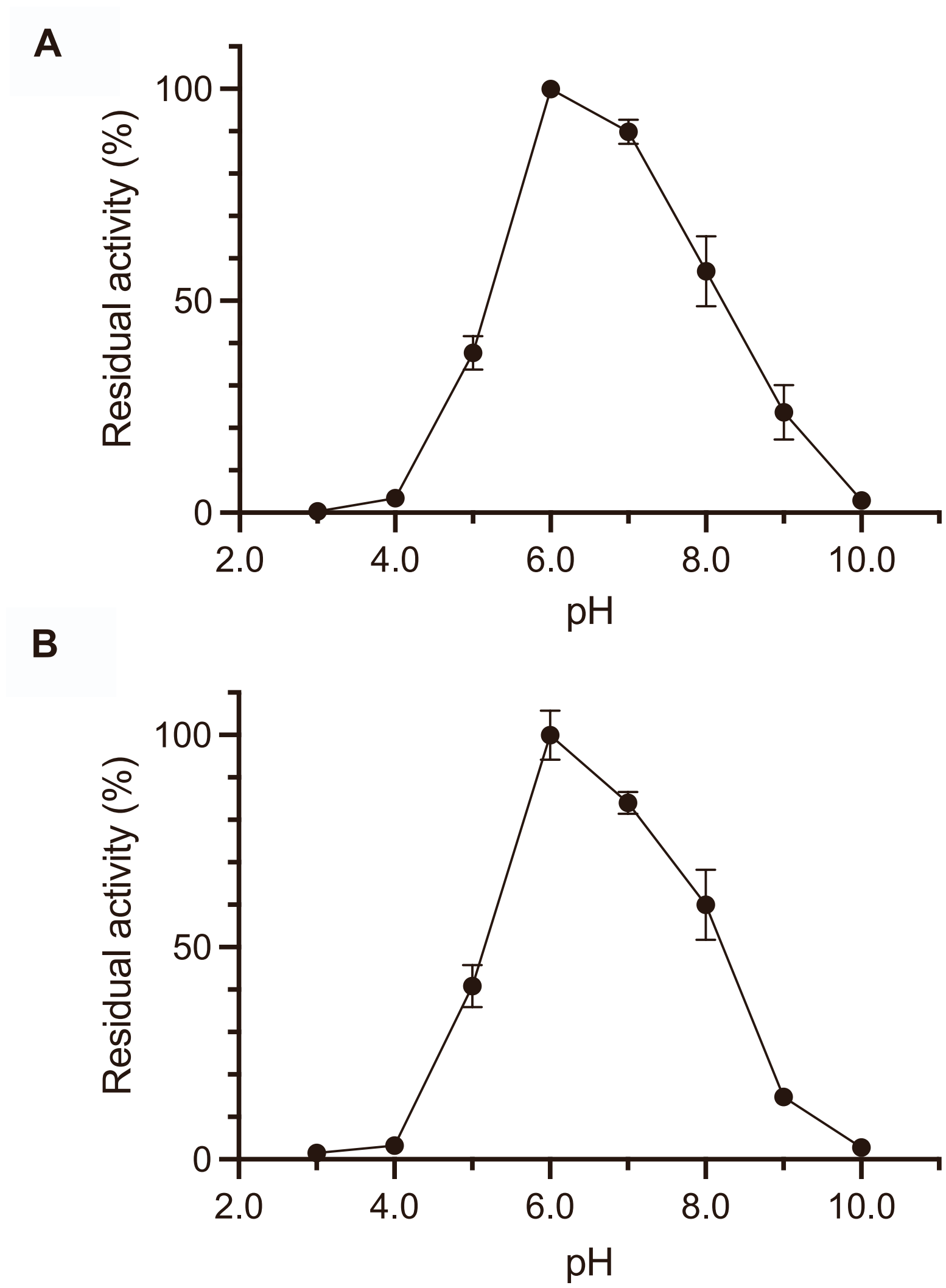

Figure S3. pH activity profiles for *Cgl*UPO$_{Pic}$ (**A**) and AckBar mutant (**B**) measured with 2 mM DMP and 2 mM H_2O_2 in 100 mM Britton and Robinson buffer at different pHs from 3.0 to 10.0. Reactions were performed in plates measuring absorbance at 469 nm. Each point represents the mean and standard deviation from 3 independent experiments.

A

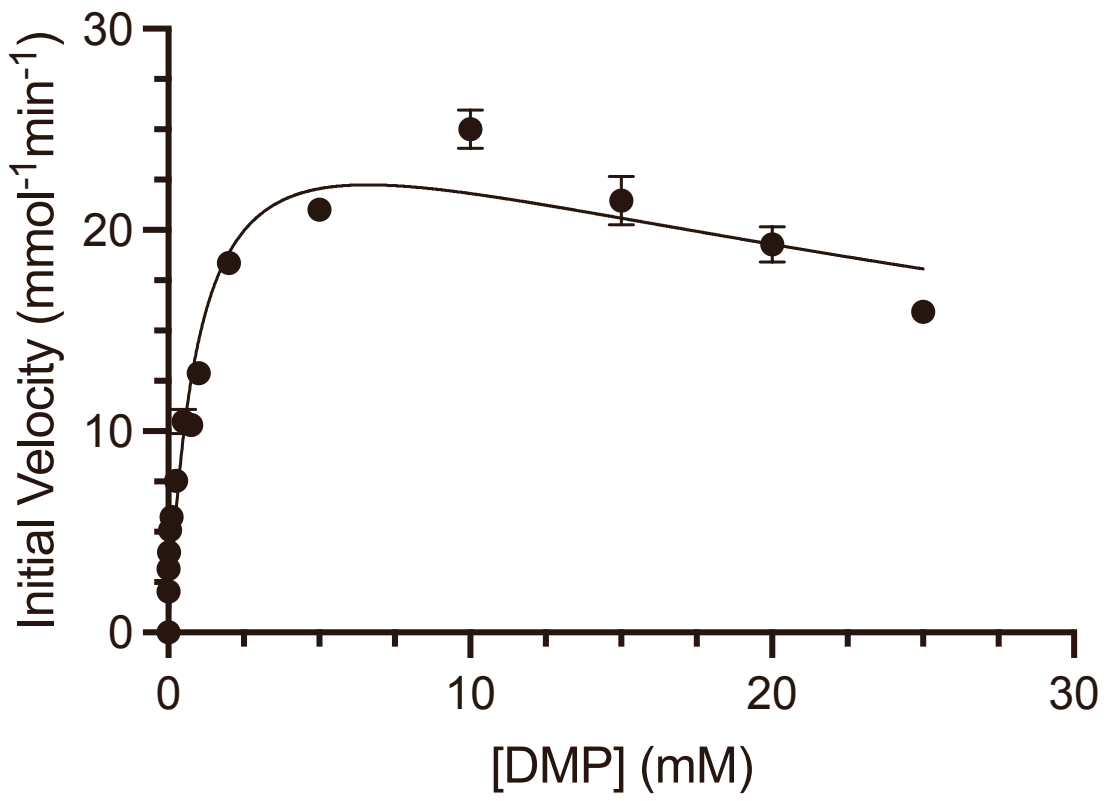

B

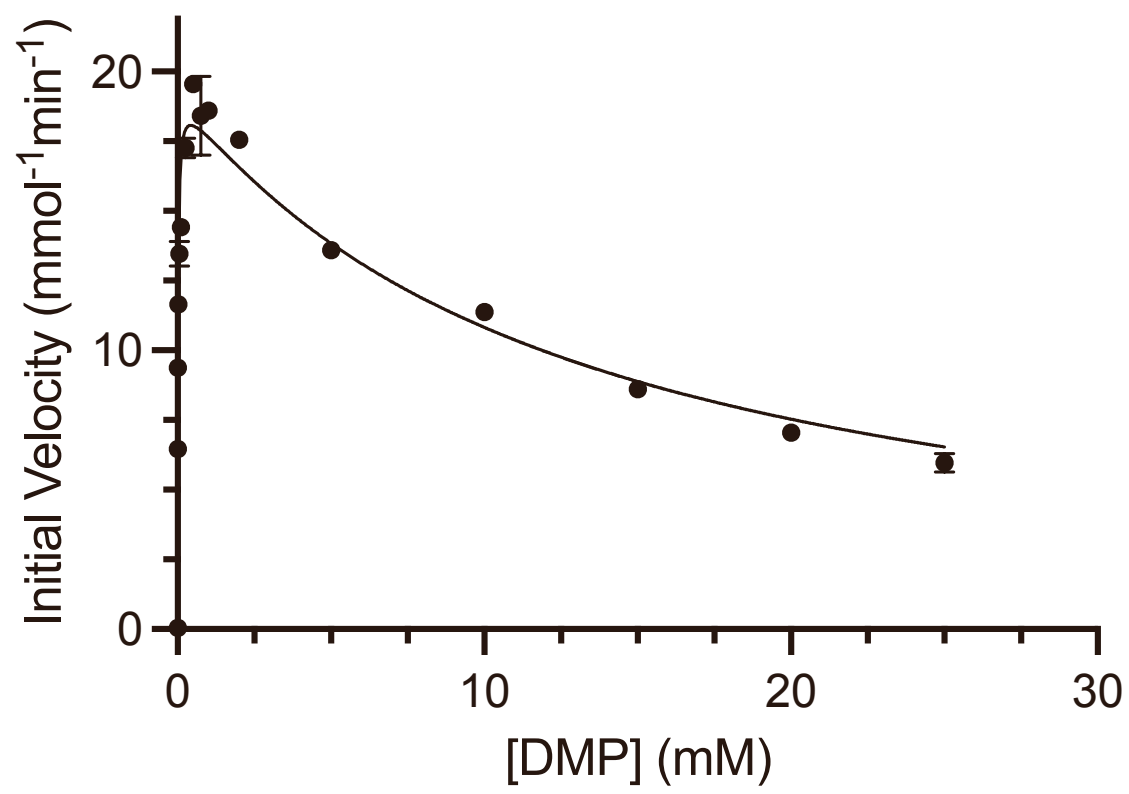

R^2 : 0.921
K_m (mM): 0.959 ± 0.199
k_{cat} (s^{-1}): 74.58 ± 5.06
k_{cat} /K_m ($mM^{-1}s^{-1}$): 78 ± 17
K_i (mM): 45.41 ± 18.88

R^2: 0.960
K_m (mM): 0.0142 ± 0.0013
k_{cat} (s): 68.63 ± 1.11
k_{cat} /K_m ($mM^{-1}s^{-1}$): 4833 ± 449
K_i (mM): 12.84 ± 0.86

C

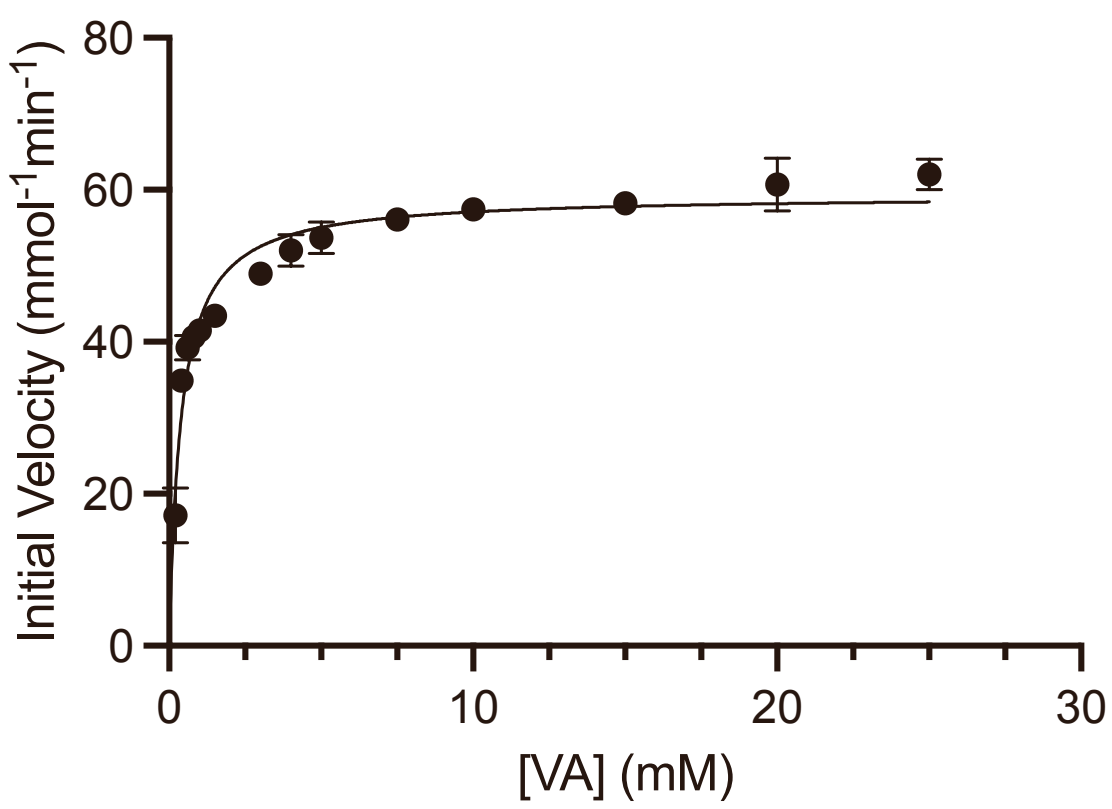

D

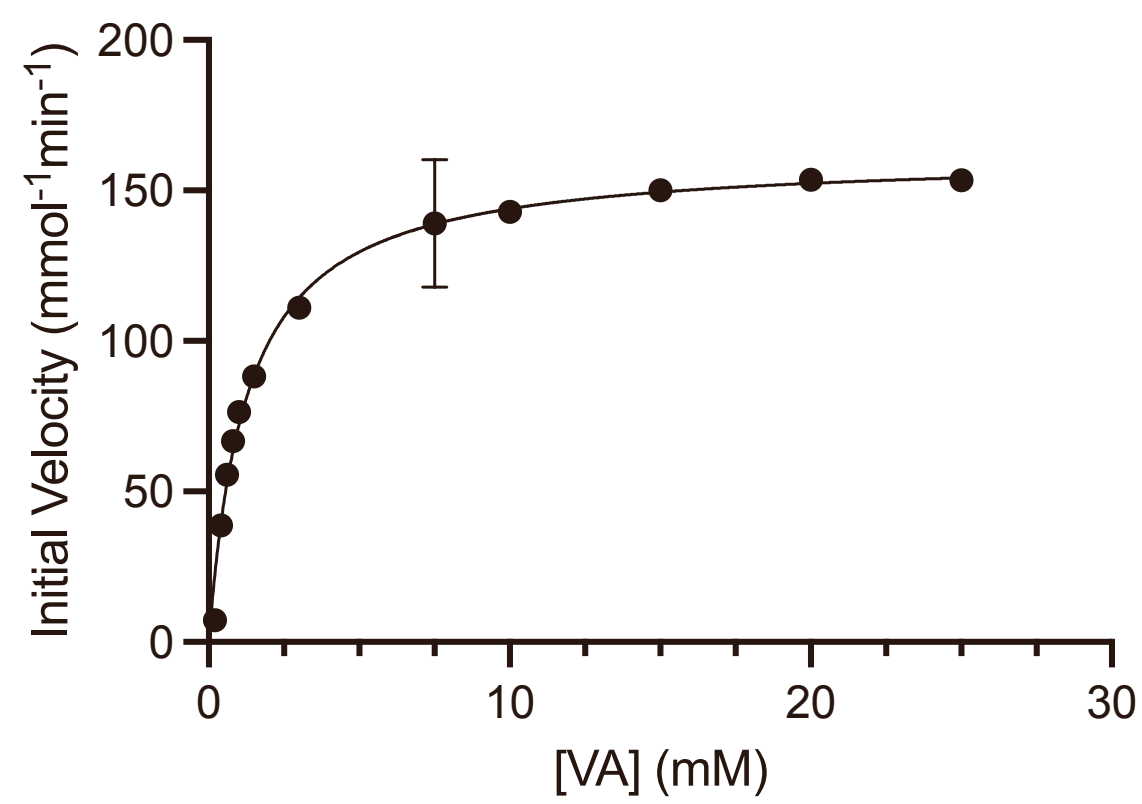

R^2: 0.936
K_m (mM): 0.3858 ± 0.023
k_{cat} (s^{-1}): 6.14 ± 0.06
k_{cat}/K_m ($mM^{-1}s^{-1}$): 15.92 ± 0.97

R^2: 0.980
K_m (mM): 1.283 ± 0.061
k_{cat} (s): 11.67 ± .13
k_{cat}/K_m ($mM^{-1}s^{-1}$): 9.09 ± 0.44

Figure S4. Determination of kinetic constants for *Cgl*UPO_{Pic} and AckBar. Kinetic values with DMP for *Cgl*UPO_{Pic} (**A**) and AckBar mutant (**B**); and with veratryl alcohol (VA), for *Cgl*UPO_{Pic} (**C**) and AckBar (**D**). Kinetic constants were estimated in 100 mM phosphate buffer pH 6.0 for DMP and 100 mM phosphate buffer pH 7.0 for veratryl alcohol. Reactions were performed in triplicate, and substrate oxidations were followed spectrophotometrically (ε_{469} coerulignone = 27,500 M^{-1} cm^{-1} and ε_{310} veratraldehyde = 9,300 M^{-1} cm^{-1}). To calculate the K_m and k_{cat} values, the average V_{max} was represented against substrate concentration and fitted to a single rectangular hyperbola function using GraphPad Prism 10 software, where parameter a was equal to k_{cat} and parameter b was equal to K_m. For DMP, an inhibition model was found to more accurately model the data. Each point represents the mean and standard deviation from 3 independent experiments.

A

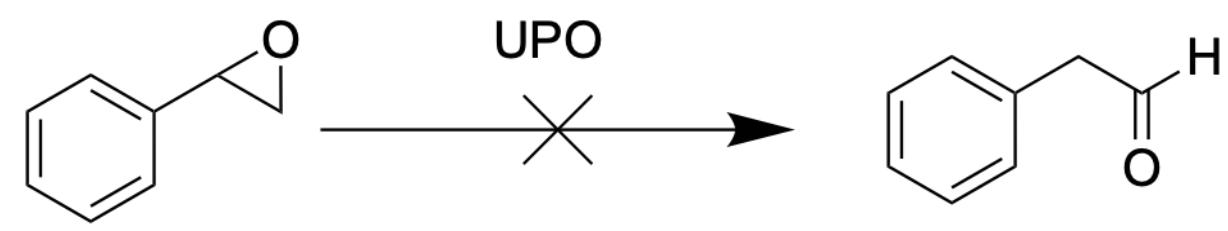

Entry	H_2O_2	Phenylacetaldehyde (% yield)	Styrene oxide (% recovery)
1	0 mM	trace	93
2	2 mM	trace	71

B

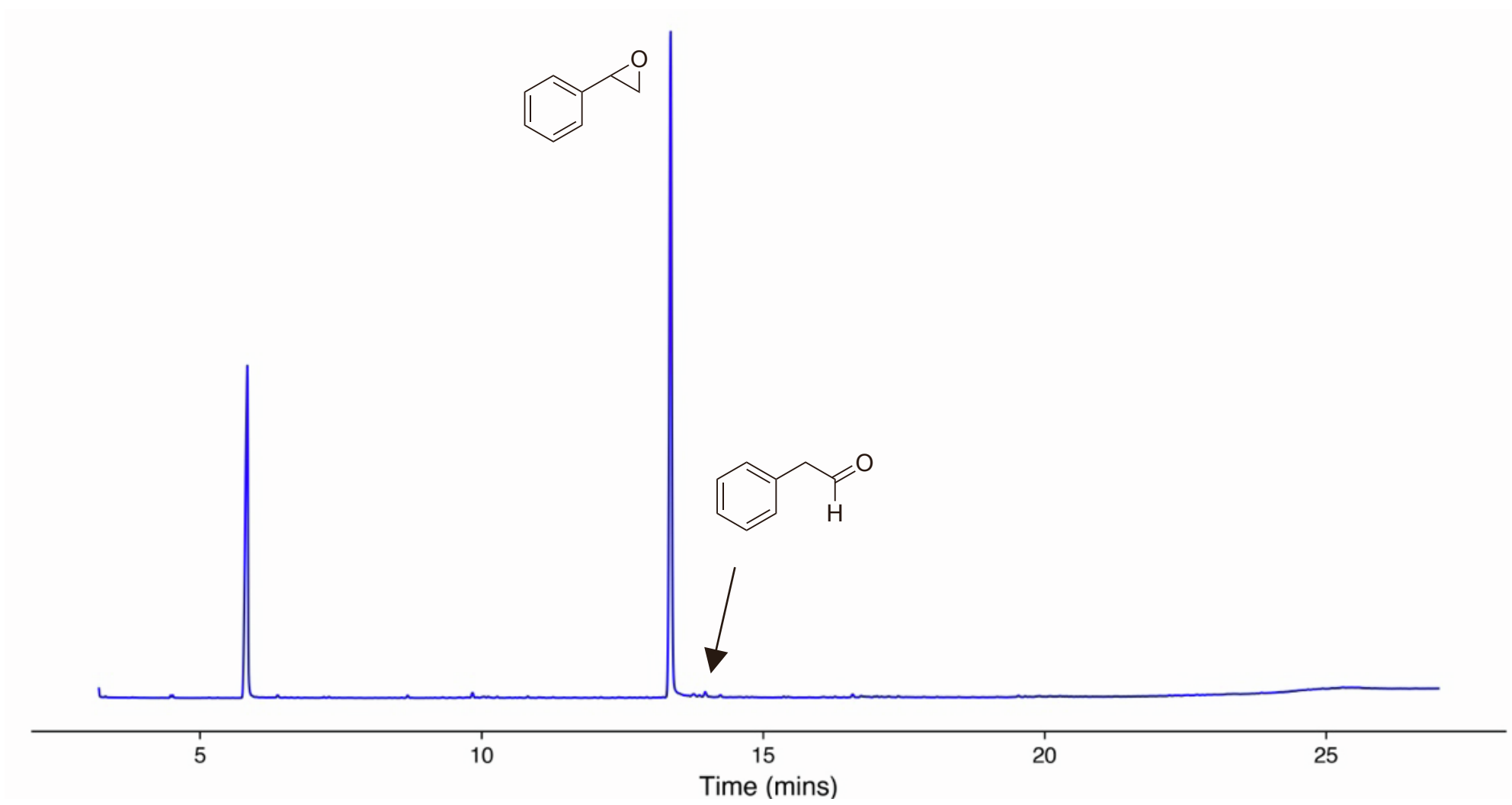

C

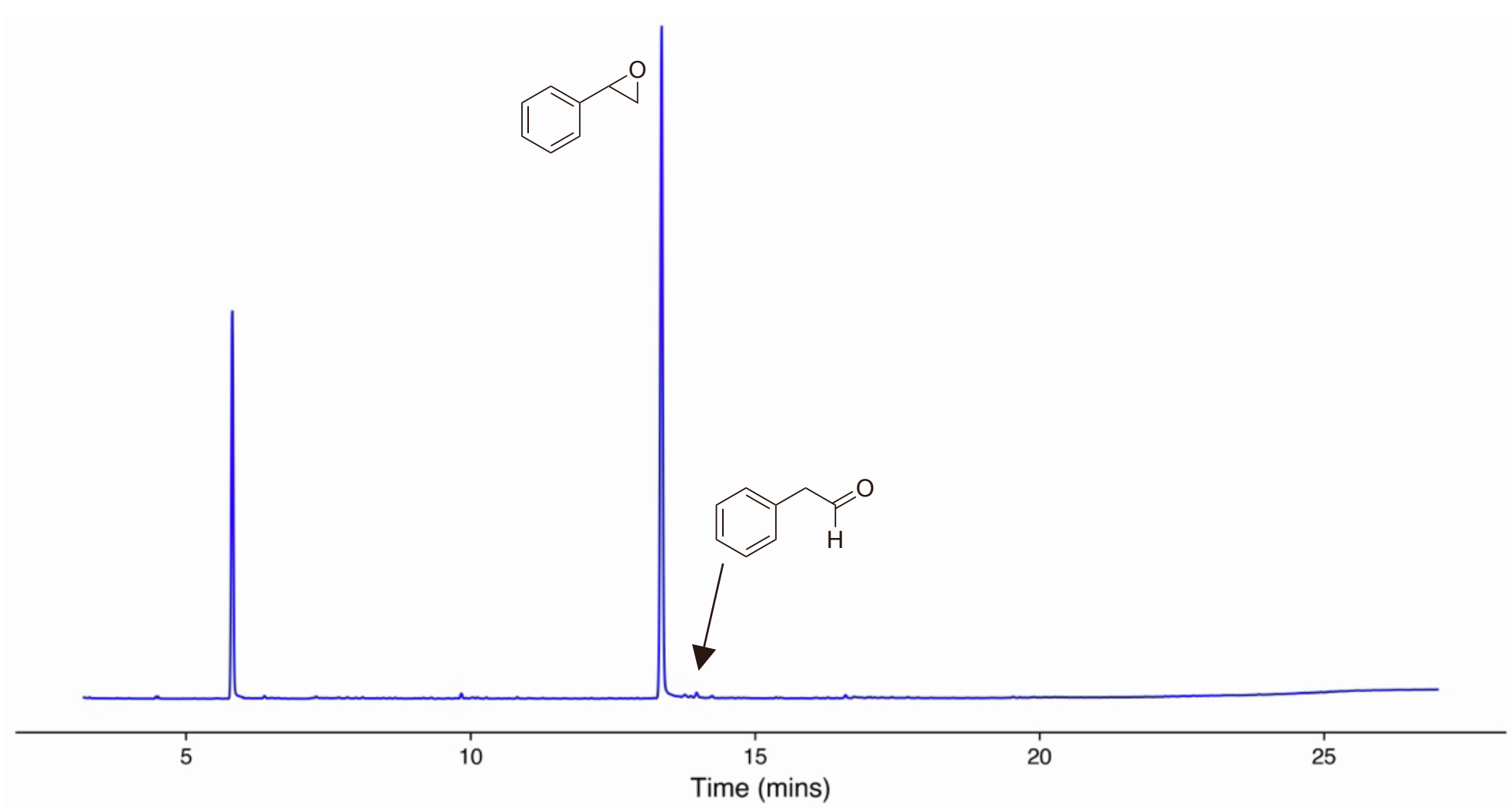

Figure S5. Control reactions (**A**) of the epoxide without (**B**) and with (**C**) the addition of H_2O_2. These reactions demonstrate that acetaldehyde is not produced through a styrene oxide intermediate and must be produced from direct oxidation of styrene in standard conditions. Reactions were run with 0.1 μM AckBar mutant, 2 mM styrene epoxide, 0 or 2 mM H_2O_2 in 50 mM phosphate pH 6.0 containing 5% acetone (v/v). Trace phenylacetaldehyde observed in both reactions are attributed to slight (<2%) impurities in the commercial authentic standard.

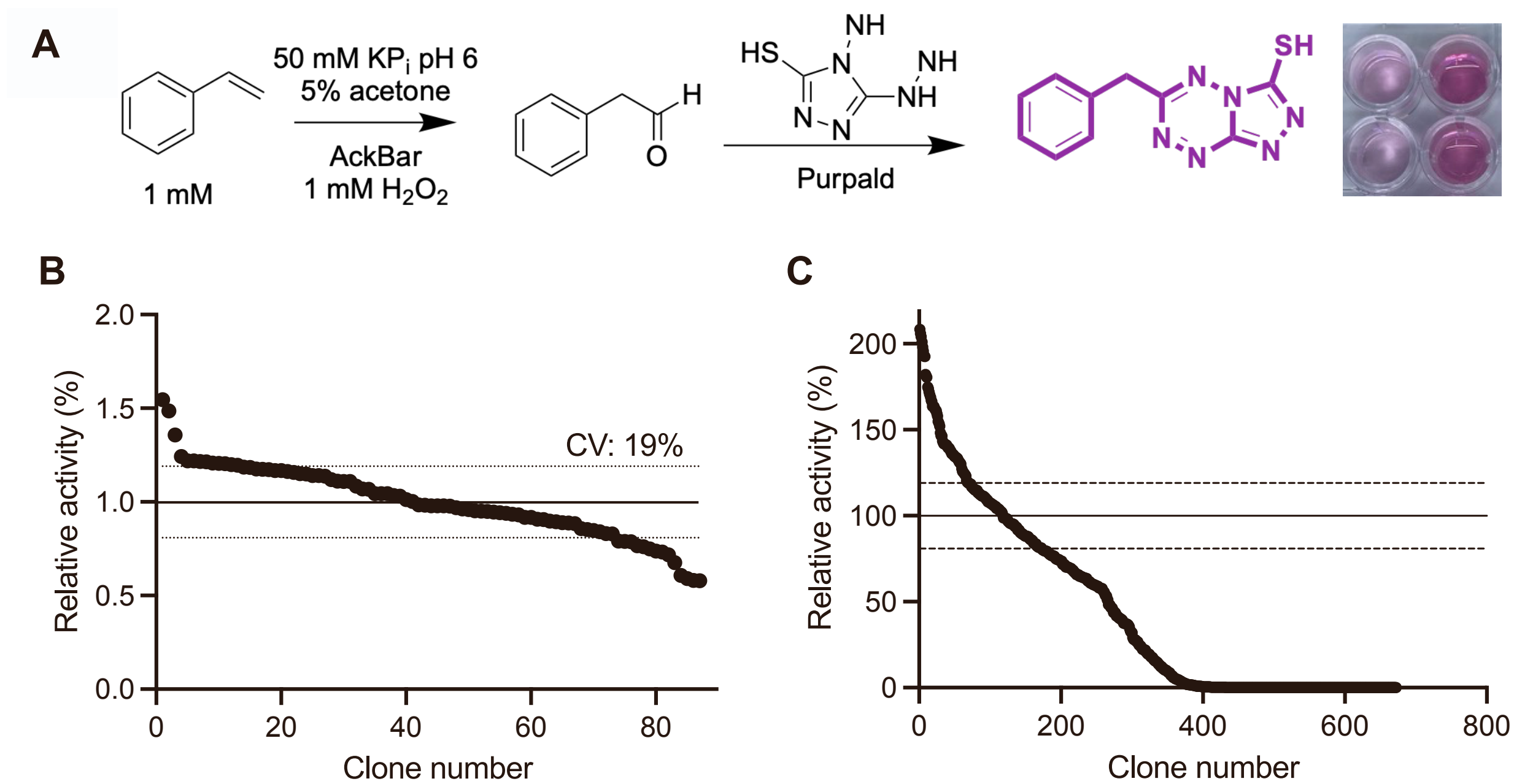

Figure S6. HTS assay for anti-Markovnikov reaction and directed evolution of AckBar mutant. (**A**) HTS assay for the detection of phenylaceltaldehyde produced with AckBar from stryrene. The phenylacetaldehyde produced reacts with Purpald leading to a purple-colored product. (**B**) Coefficient of variance of the HTS for anti-Markovnikov reaction using AckBar expressed in 96-well plates. A small *ep*PCR library of 679 clones was examined using the method described in (**A**) and (**B**), resulting in the mutagentic landscape shown in (**C**).

>*Cgl*UPO
MRTSLLPALAAVSPVLAGFDTWAPPGPYDVRGPCPMLNTLTNHGFFPHDGQDIDRETTENALF
DALHVNKTLASFLFDFALTTNPIANSTTFSLNDLGNHNVLEHDASLSRADAYHGSVLAFNHTIFE
ETKSYWTDETVTLKMAADARYYRIKSSQATNPTYQMSELGDAFTYGESAAYVVLFGDKESQTV
PRSWVEWLFEKEQLPQHLGWKRPATSFELNDLDKFMALIQNYTQEIEEPSCESRKQRRKPRG
PSHFGF*

>AckBar
MRTSLLPALAAVSPVLAGFDTWAPPGPYDVRGPCPMLNTLTNHGFFPHDGQDIDRETTENALF
DALHVNKTLASFLFDFALTTNPIANSTTFSLNDLGNHNVLEHDASLSRADAYHGSVLAFNHTIFE
ETKSYWTDETVTLKMAADARYYRIKSSQATNPTYQMSELGDALTYGESAAYVVLFGDKESQTV
PRSWVEWLFEKEQLPQHLGWKRPATSFELNDLDKFVALIQNYTQEIEEPSCESRKQRRKPRG
PSHFGF*

Figure S7. FASTA sequences of *Cgl*UPO and AckBar proteins. Highlighted in cyan are the native *Cgl*UPO signal peptides and in green are the mutations introduced into AckBar.

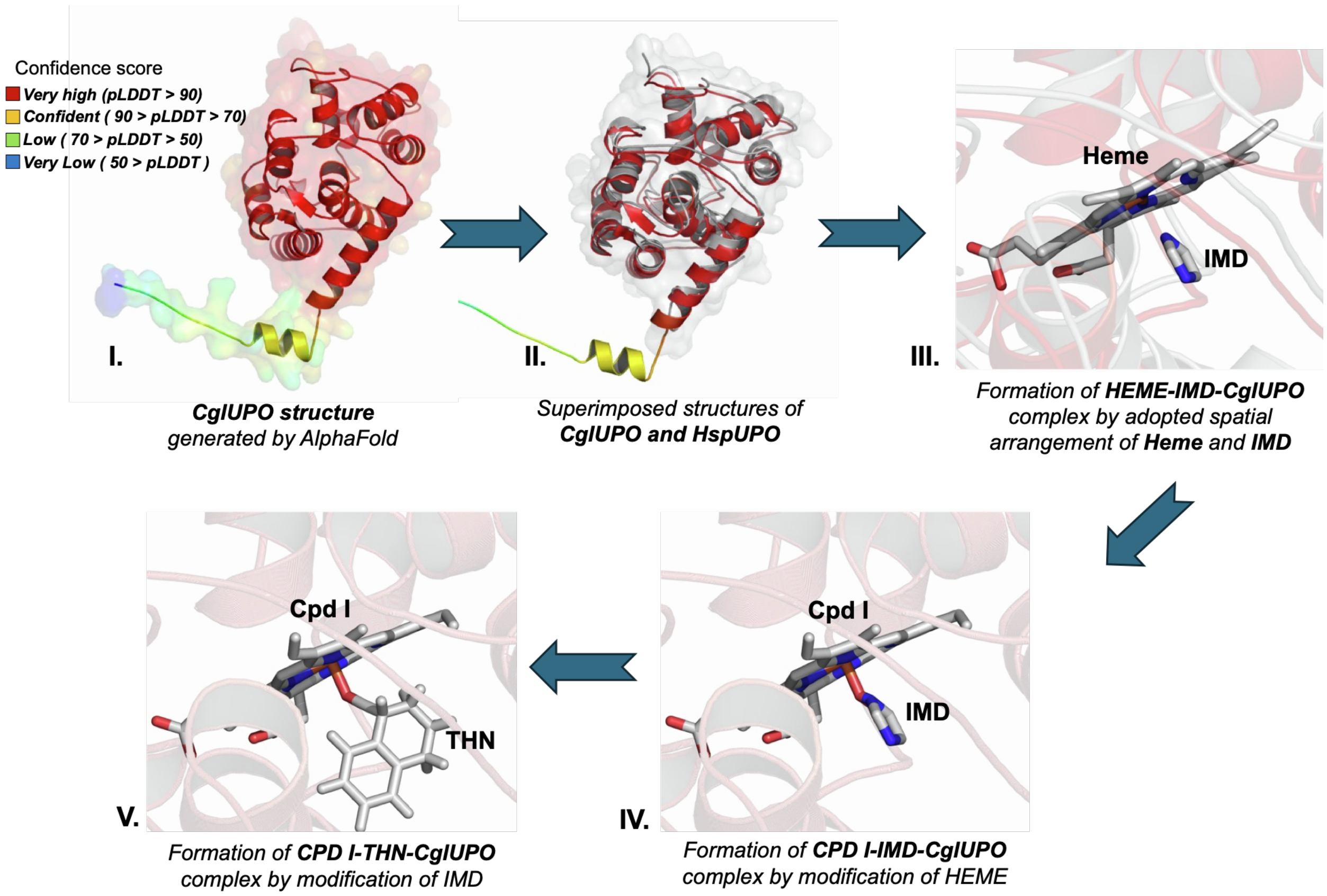

Figure S8. Detailed procedure for constructing the *Cgl*UPO complex with Compound I (Cpd I), and the tetrahydronaphthalene (THN) substrate. **I.** Generation of the *Cgl*UPO protein structure using AlphaFold2. **II.** Structural overlay of the generated model with the crystal structure of unspecific peroxygenase from *Hypoxylon* sp. EC38 (*Hsp*UPO) complexed with protoporphyrin IX (HEME) and imidazole (IMD). **III.** Formation of HEME-IMD-*Cgl*UPO complex. **IV.** Construction of Cpd I based on the Heme cofactor scaffold. **V.** Placement of the THN molecule according to the position of IMD.

```
CglUPO      ----------------------GFDTWAPPGPYDVRGPCPMLNTLTNHGFFPHDGQDIDR        38
HspUPO      MKSLSFSLALGFGSTLVYSAPSPSSGWQAPGPNDVRAPCPMLNTLANHGFLPHDGKGITV        60
                                    . *  *** ***.********:****:****:.*

CglUPO      ETTENALFDALHVNKTLASFLFDFALTTNPIANSTTFSLNDLGNHNVLEHDASLSRADAY        98
HspUPO      NKTIDALGSALNIDANLSTLLFGFAATTNPQPNATFFDLDHLSRHNILEHDASLSRQDSY        120
            :.* :** .**::: .*:::**.** ****  *:* *.*:.*..**:********* *:*

CglUPO      HGSVLAFNHTIFEETKSYWTDETVTLKMAADARYYRIKSSQATNPTYQMSELGDAFTYGE        158
HspUPO      FGPADVFNEAVFNQTKSFWTGDIIDVQMAANARIVRLLTSNLTNPEYSLSDLGSAFSIGE        180
            .* . .**.::*::***:**.: : ::***:**  *: :*: *** *.:*:**.**: **

CglUPO      SAAYVVLFGDKESQTVPRSWVEWLFEKEQLPQHLGWKRPATSFELNDLDKFMALIQNYTQ        218
HspUPO      SAAYIGILGDKKSATVPKSWVEYLFENERLPYELGFKRPNDPFTTDDLGDLSTQIINAQH        240
            ****: ::***:* ***:****:***:*:** .**:***   *  :**..: : * *  :

CglUPO      EIEEPS-CESRKQRRKPRGPSHFGF-  242
HspUPO      FPQSPGKVEKRGDTRC-----PYGYH  261
              :.*.  *.* : *       :*:
```

Figure S9. Comparative sequence alignment of *Cgl*UPO and *Hsp*UPO enzymes obtained with CLUSTAL Omega.[1]

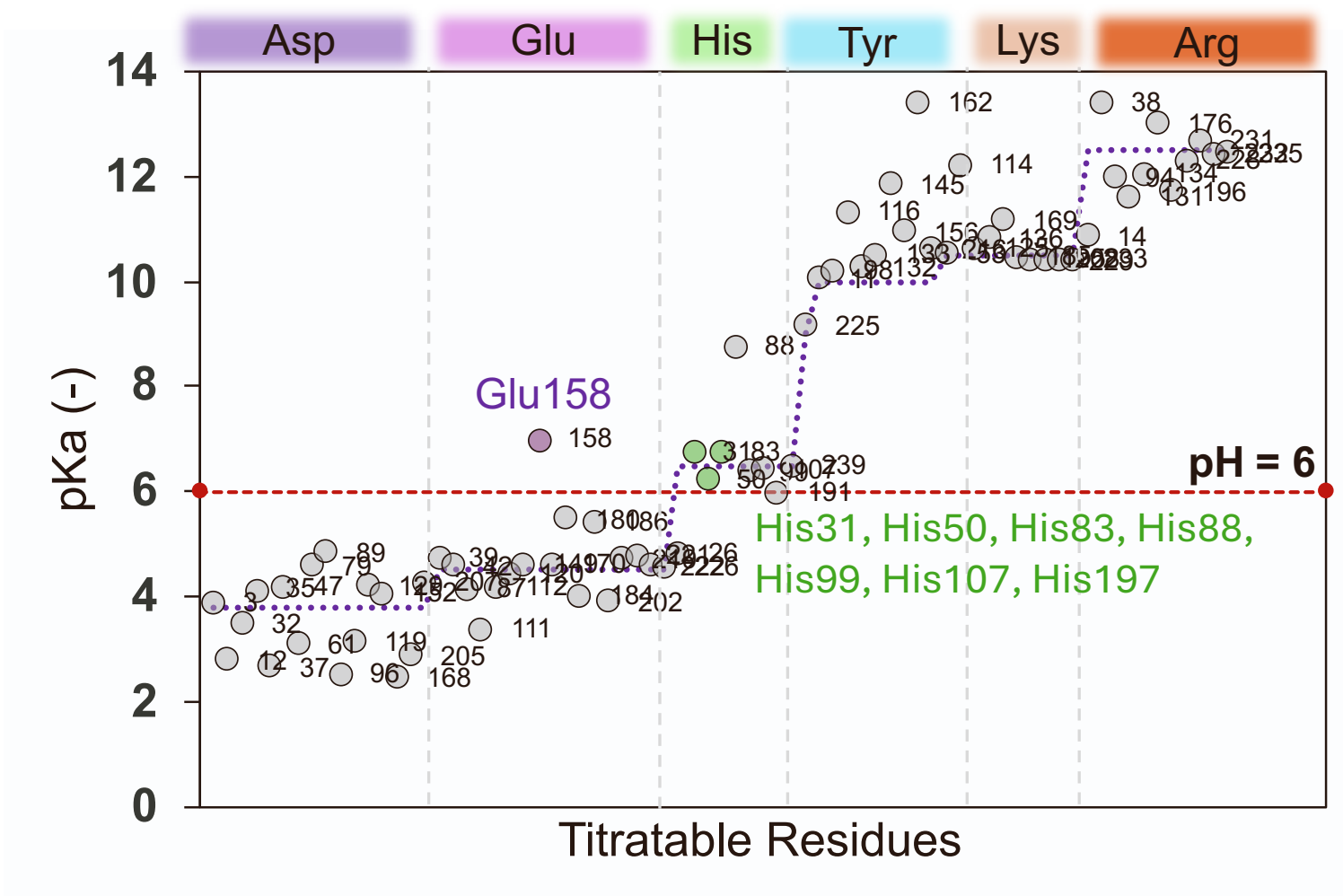

Figure S10. pKa values of titratable residues of *Cgl*UPO computed with PROPKA 3.1.

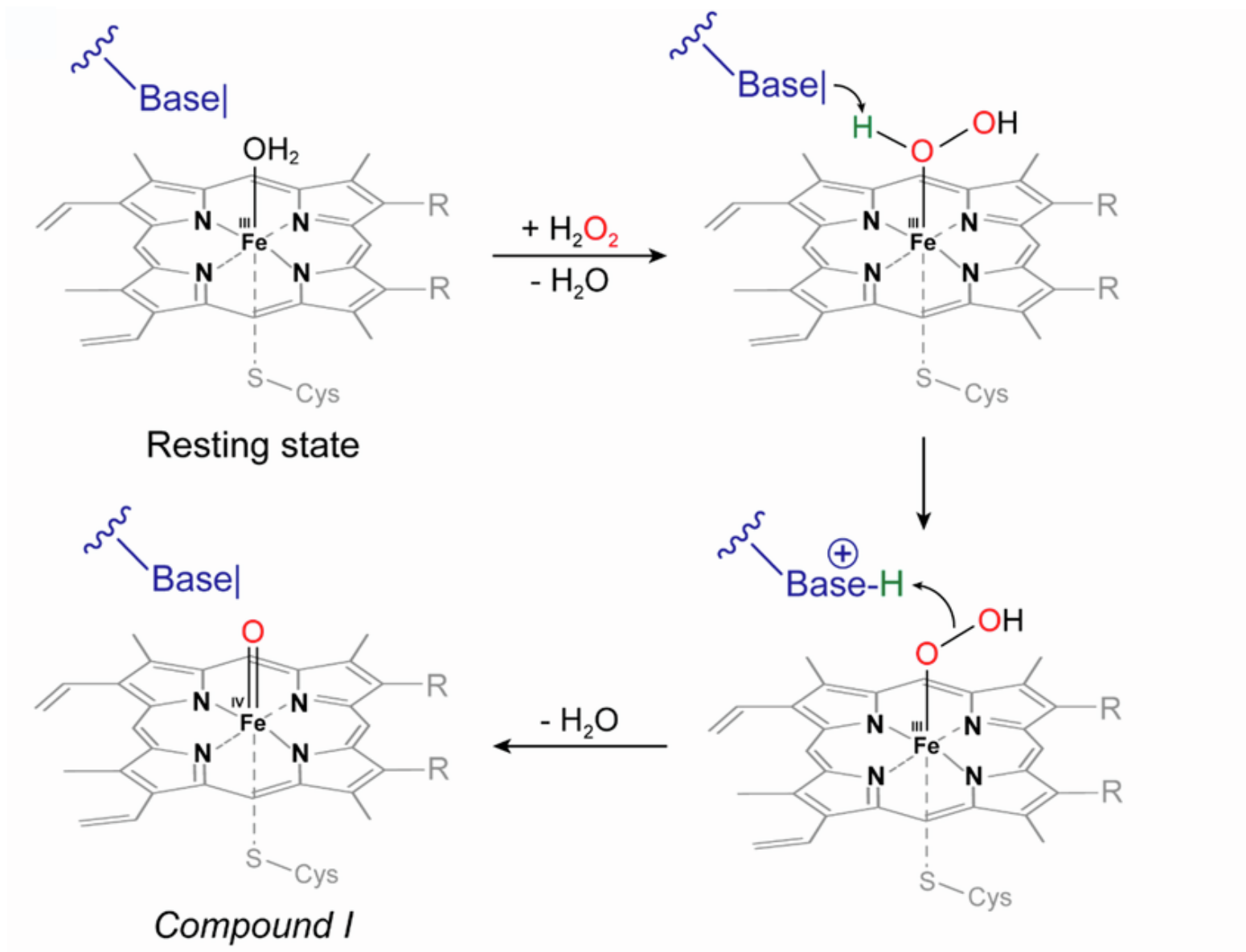

Figure S11. General mechanism of Compound I (Cpd I) formation. In the *Cgl*UPO and AckBar, the base that abstracts a proton from hydrogen peroxide and afterward facilitates water departure is Glu158; therefore, this residue is deprotonated in both systems.

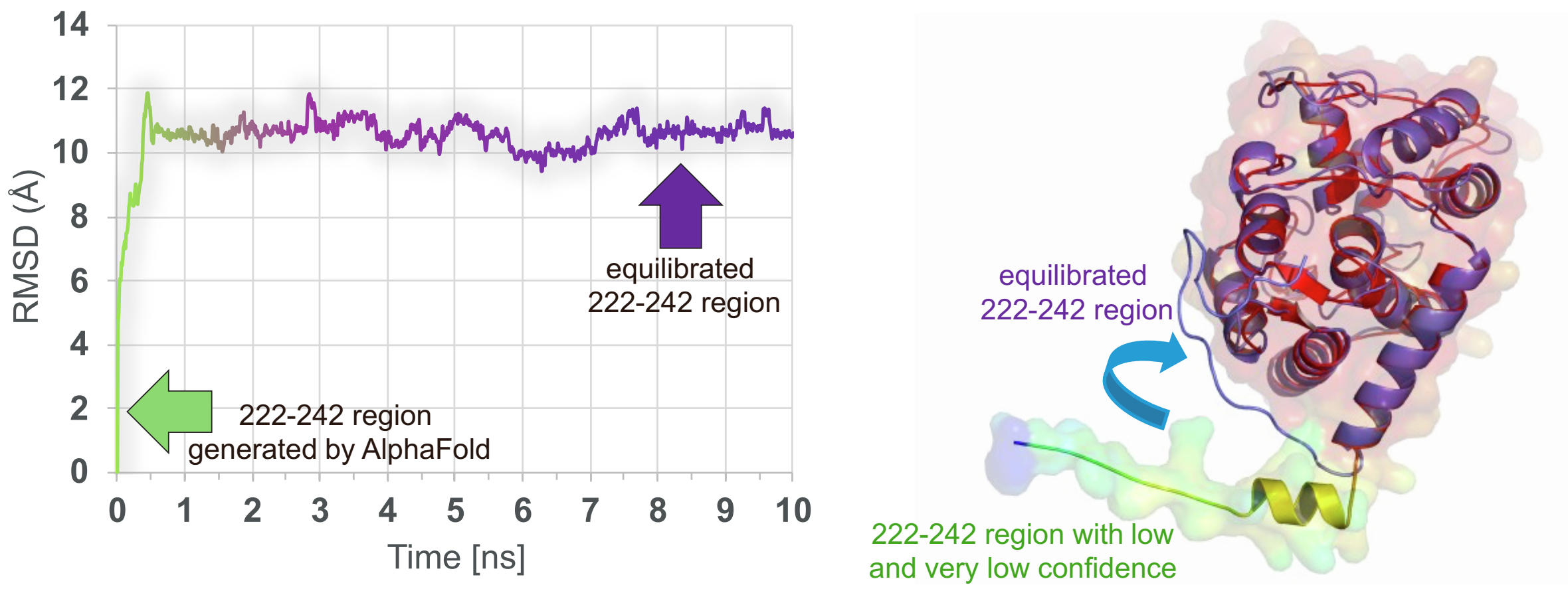

Figure S12. Root-mean-squared-deviation (in Å) along 10 ns of NVT GBIS-MD simulations for atoms of *Cgl*UPO fragment with originally established low and very low confidence (70>pLDDT>50 and 50>pLDDT, respectively)

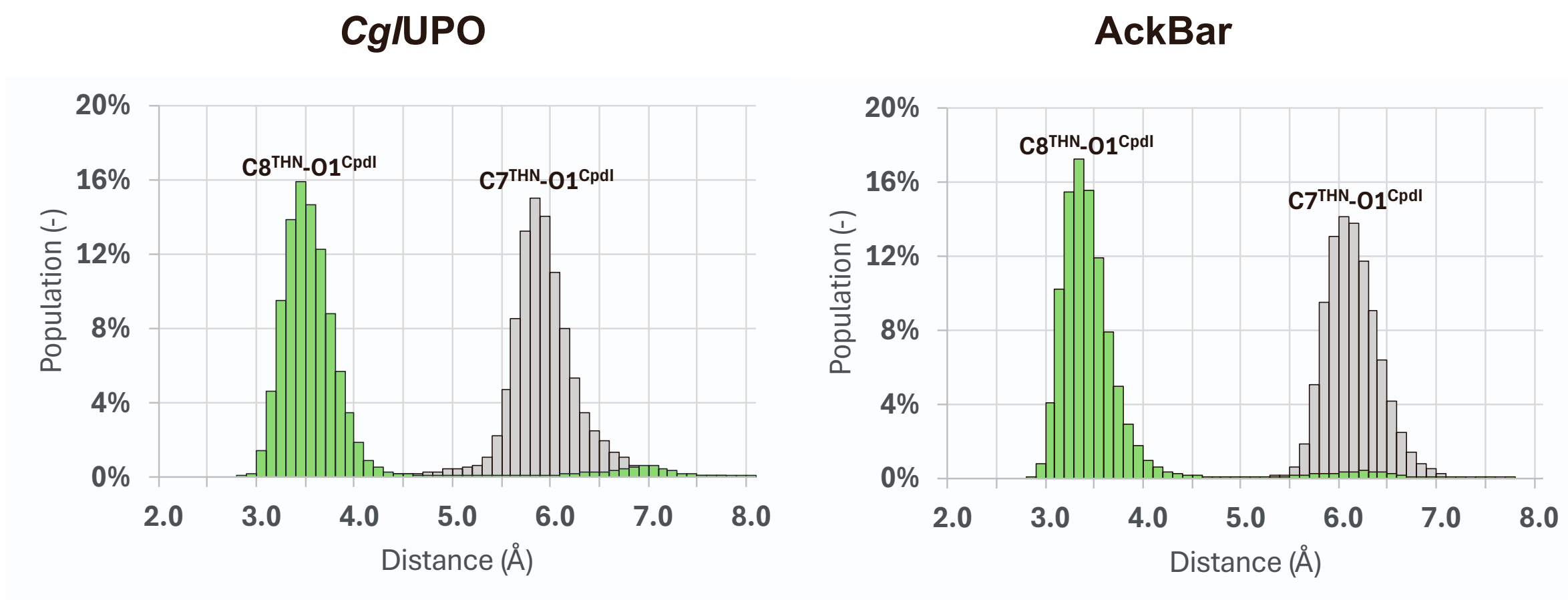

Figure S13. Distribution of O1^{CpdI}····C7^{THN} (in grey) and O1^{CpdI}····C8^{THN} (in green) distances in *Cgl*UPO and AckBar in all three replicas of MD simulations (3 x 0.5 μs) computed for 75,000 structures.

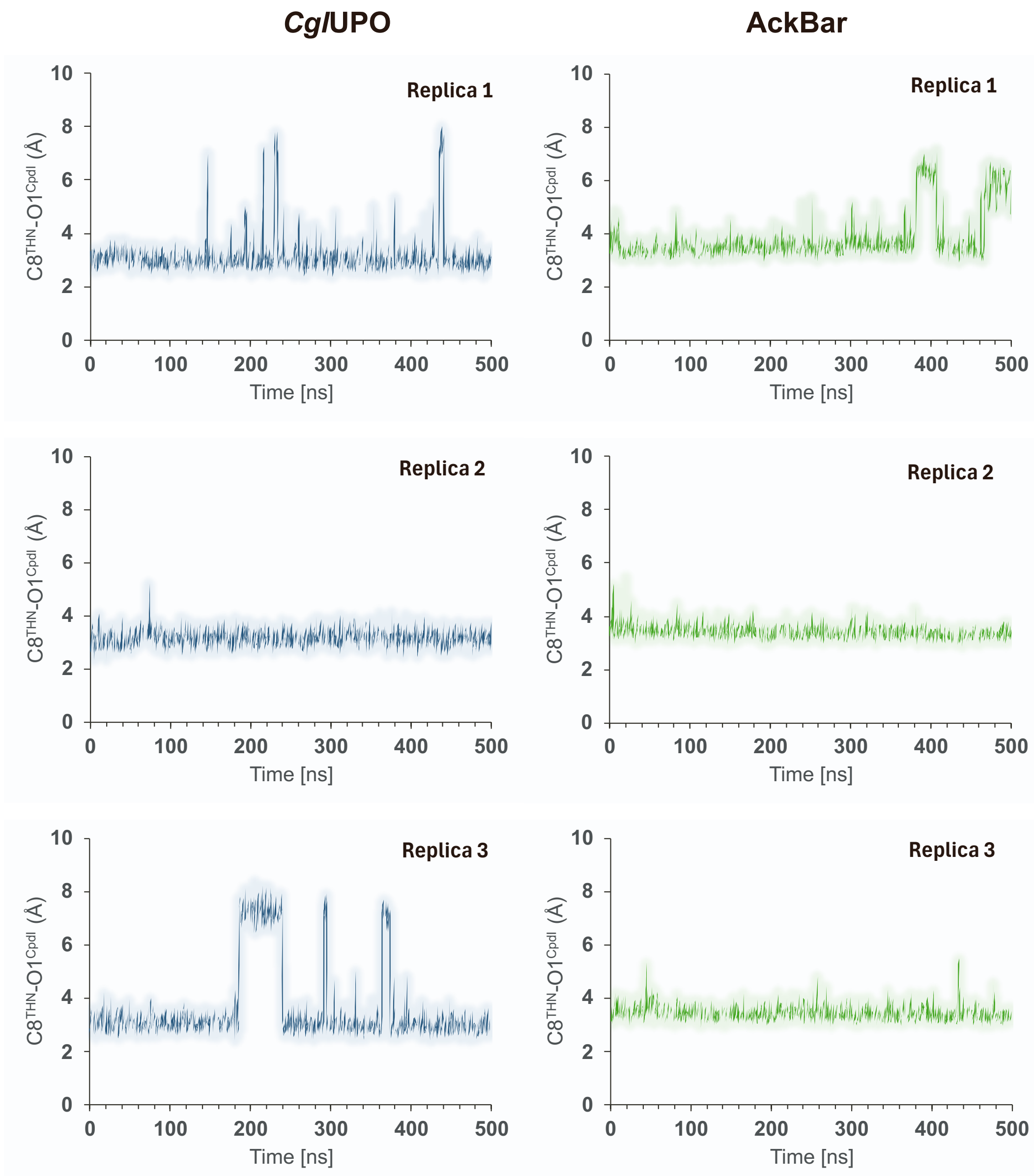

Figure S14. Evolution of the distance between O1 of Cpd I and C8 of THN during MD simulations explored in three independent replicas in *Cgl*UPO (on the left) and AckBar (on the right).

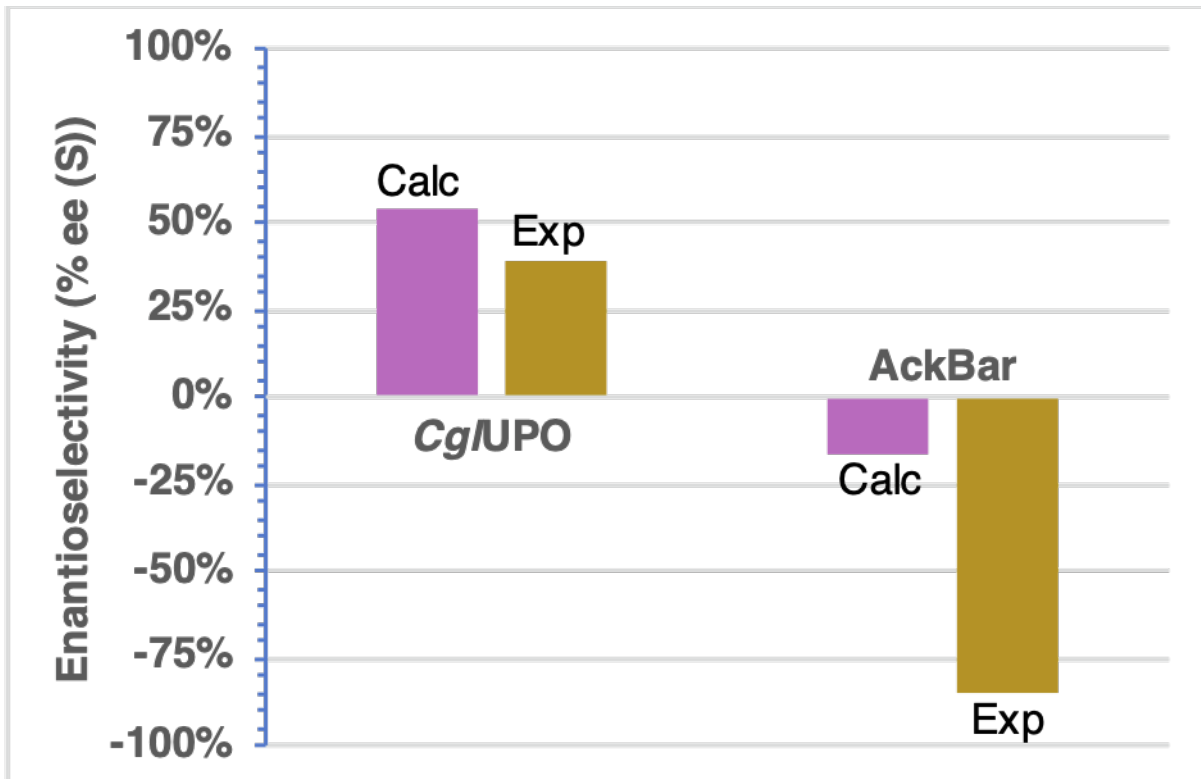

Conditions	*ee CglUPO*	ee AckBar
Calculated	54%	-16%
Experimental	39%	-85%

Figure S15. Bar charts showing the computed and experimentally determined enantiomeric excess (ee (*R*)) of THN oxidation products for *Cgl*UPO and AckBar. Products were computed based on distance, $d_{O1\text{-}H,}$ between O1 oxygen atom of Cpd I and H7 or H8 of THN, taking into account all 75,000 configurations where $d_{O1\text{-}H}$ < 2.8, compared to experimentally determined values.

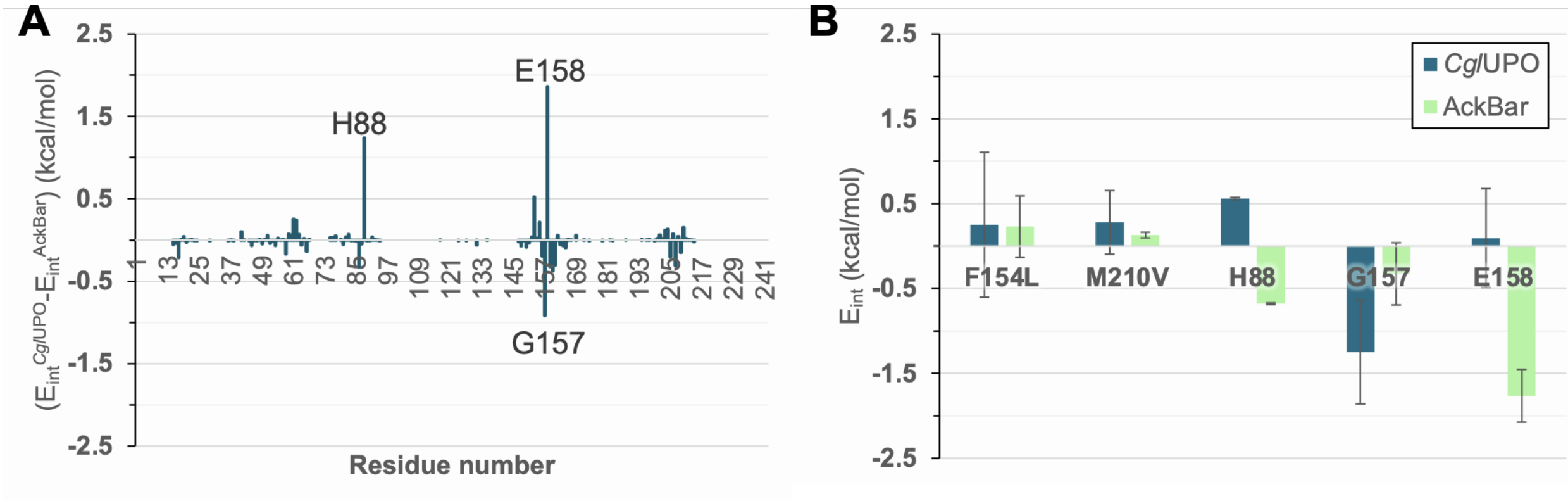

Figure S16. (**A**) Per-residue interaction energy (E_{int}) differences between THN and the amino acids of *Cgl*UPO and AckBar. (**B**) E_{int} values for selected mutated residues and those showing the most significant differences between the *Cgl*UPO and AckBar variants. All E_{int} and their standard deviations were computed for 125,000 snapshots generated in 5 replicas of 0.5 μs MD simulations.

SUPPLEMENTAL TABLES

Table S1. Benchmarking of evolved variants with model compounds.

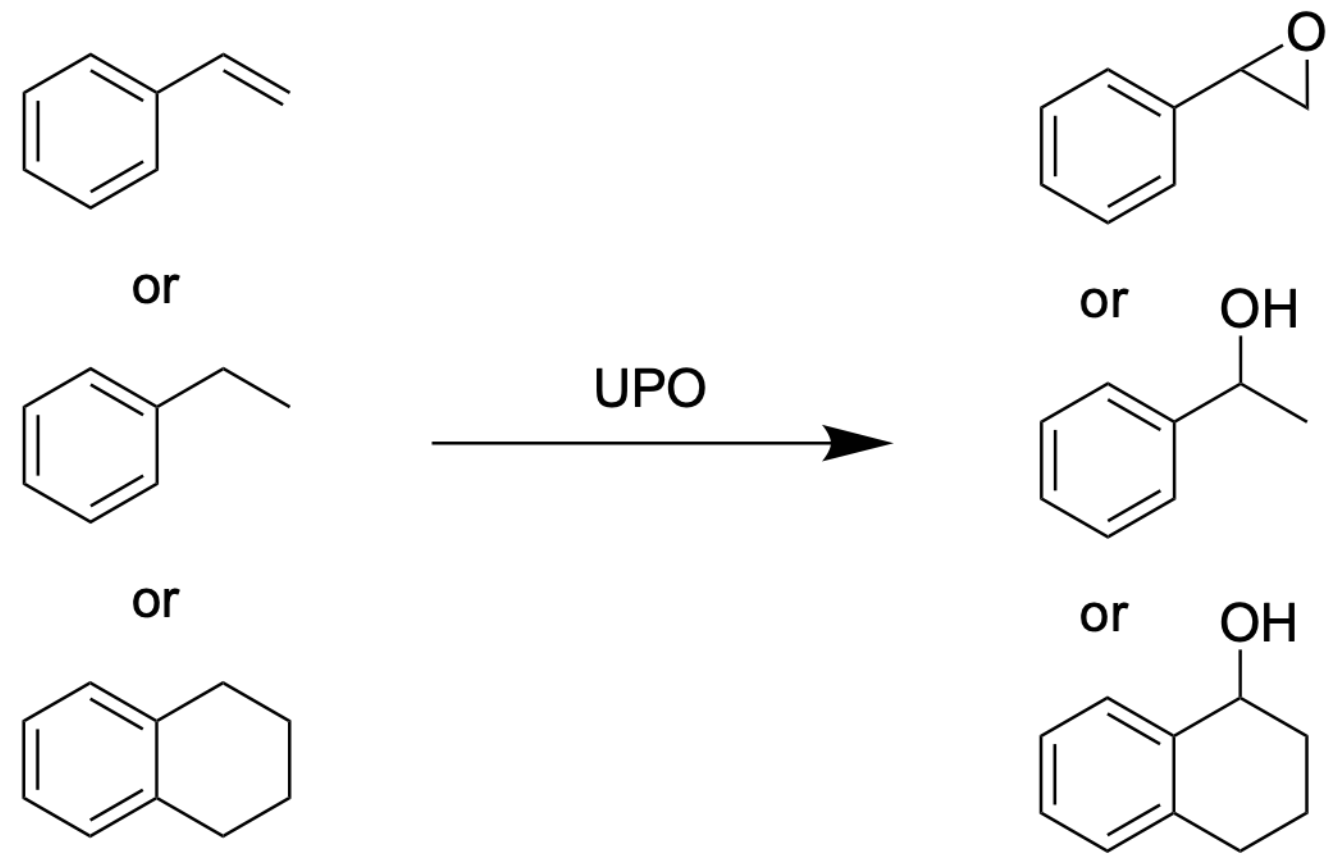

	Substrate		
Variant	Ethylbenzene (e.r.)	Styrene (epoxide e.r.)	Tetrahydronaphthalene (e.r.)
*Cgl*UPO$_{wt}$	66:34	31:69	60:40
*Cgl*UPO$_{Sac}$	68:32	30:70	63:37
11G4	69:31	28:72	64:36
9G3	72:28	28:72	72:28
4B10	n.d.	26:74	57:43
3H9	69:31	28:72	70:30
AckBar	n.d.	20:80	7:93

Reactions were performed with enzyme supernatants from expression in *S. cerevisiae* in 100 mM phosphate buffer pH 7.0 containing 5% acetonitrile (v/v), 1 mM substrates and 1 mM H_2O_2 in 300 µL final volume. The reactions were performed in 1.5 mL tubes, shaken at 30 °C for 1 hour, before they were extracted with 1 volume of ethyl acetate and analyzed by GC/MS using the chiral methods described in the chromatography methods section. E.r., enantiomeric ratio (*R*:*S*), n.d. not determined.

Table S2. Missing force field parameters for the tetrahydronaphthalene (THN) generated using GAFF with the AM1-BCC partial atomic charges.

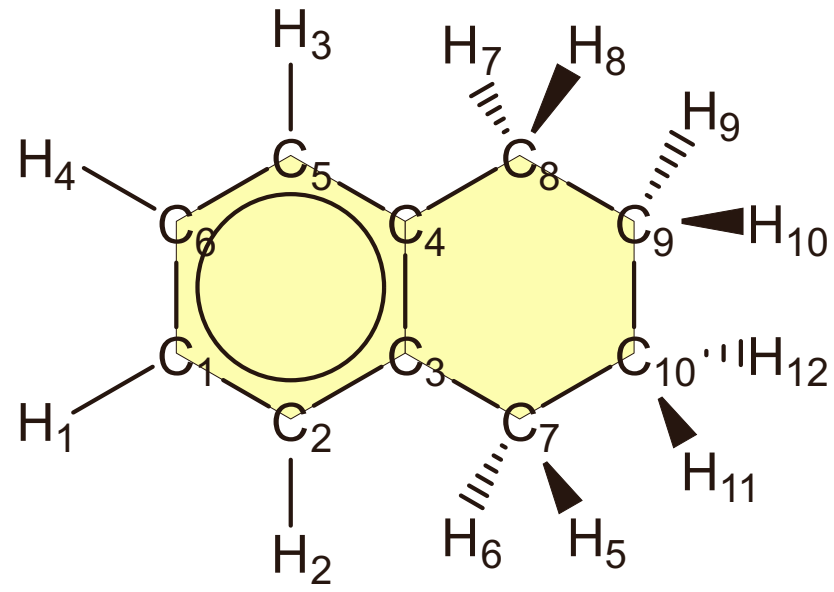

Atom name	Atom type		Atomic charge		Atom name	Atom type		Atomic charge	
C2	ca		-0.1300		H5	hc		0.0492	
H2	ha		0.1290		H6	hc		0.0492	
C1	ca		-0.1310		C10	c3		-0.0754	
H1	ha		0.1290		H11	hc		0.0427	
C6	ca		-0.1310		H12	hc		0.0427	
H4	ha		0.1290		C9	c3		-0.0754	
C5	ca		-0.1300		H9	hc		0.0427	
H3	ha		0.1290		H10	hc		0.0427	
C4	ca		-0.0703		C8	c3		-0.0341	
C3	ca		-0.0703		H7	hc		0.0492	
C7	c3		-0.0341		H8	hc		0.0492	
FF Parameters									
BOND					ANGLE				
ca-ha	345.8		1.086		ca-ca-ha	48.2		119.88	
ca-ca	461.1		1.398		ca-ca-ca	66.6		120.02	
c3-ca	321		1.516		c3-ca-ca	63.5		120.77	
c3-hc	330.6		1.097		c3-c3-ca	63.1		112.07	
c3-c3	300.9		1.538		ca-c3-hc	46.8		110.47	
					c3-c3-hc	46.3		109.8	
					c3-c3-c3	62.9		111.51	
					hc-c3-hc	39.4		107.58	
DIHEDRAL									
ca-ca-ca-ha	4	14.5000	180	2	c3-c3-c3-ca	9	1.4	0	3
ca-ca-ca-ca	4	14.5000	180	2	c3-c3-c3-hc	1	0.16	0	3
hc-c3-ca-ca	6	0.0000	0	2	c3-c3-c3-c3	1	0.18	0	-3
c3-c3-ca-ca	6	0.0000	0	2	c3-c3-c3-c3	1	0.25	180	-2
ha-ca-ca-ha	4	14.5000	180	2	c3-c3-c3-c3	1	0.2	180	1
c3-ca-ca-ha	4	14.5000	180	2	hc-c3-c3-hc	1	0.15	0	3
c3-ca-ca-ca	4	14.5000	180	2	c3-ca-ca-c3	4	14.5	180	2
ca-c3-c3-hc	9	1.4	0	3					
IMPROPER					NONBON				
ca-ca-ca-ha	1.1		180	2	ca	1.908		0.086	
c3-ca-ca-ca	1.1		180	2	ha	1.459		0.015	
					c3	1.908		0.1094	
					hc	1.487		0.0157	

Table S3. Standard (pK_a^{model}) and computed (pK_a^{calc}) pKa values of titratable residues of *Cgl*UPO.

Residue	Number	pK_a^{calc}	pK_a^{model}	Residue	Number	pK_a^{calc}	pK_a^{model}
ASP	3	3.89	3.8	HIS	88	8.75	6.5
ASP	12	2.81	3.8	HIS	99	6.39	6.5
ASP	32	3.48	3.8	HIS	107	6.43	6.5
ASP	35	4.15	3.8	HIS	191	5.99	6.5
ASP	37	2.67	3.8	HIS	239	6.49	6.5
ASP	47	4.19	3.8	CYS	225	9.15	9
ASP	61	3.1	3.8	TYR	11	10.05	10
ASP	79	4.59	3.8	TYR	98	10.21	10
ASP	89	4.84	3.8	TYR	116	11.31	10
ASP	96	2.53	3.8	TYR	132	10.28	10
ASP	119	3.14	3.8	TYR	133	10.51	10
ASP	129	4.22	3.8	TYR	145	11.85	10
ASP	152	4.06	3.8	TYR	156	10.96	10
ASP	168	2.47	3.8	TYR	162	13.41	10
ASP	205	2.9	3.8	TYR	216	10.61	10
ASP	207	4.28	3.8	LYS	53	10.52	10.5
GLU	39	4.75	4.5	LYS	114	12.22	10.5
GLU	42	4.6	4.5	LYS	125	10.64	10.5
GLU	87	4.15	4.5	LYS	136	10.83	10.5
GLU	111	3.38	4.5	LYS	169	11.18	10.5
GLU	112	4.19	4.5	LYS	185	10.47	10.5
GLU	120	4.44	4.5	LYS	195	10.42	10.5
GLU	149	4.61	4.5	LYS	208	10.42	10.5
GLU	158	7.00	4.5	LYS	229	10.41	10.5
GLU	170	4.62	4.5	LYS	233	10.42	10.5
GLU	180	5.5	4.5	ARG	14	10.88	12.5
GLU	184	4.02	4.5	ARG	38	13.41	12.5
GLU	186	5.42	4.5	ARG	94	11.98	12.5
GLU	202	3.9	4.5	ARG	131	11.62	12.5
GLU	219	4.73	4.5	ARG	134	12.04	12.5
GLU	221	4.79	4.5	ARG	176	13	12.5
GLU	222	4.6	4.5	ARG	196	11.72	12.5
GLU	226	4.57	4.5	ARG	228	12.3	12.5
HIS	26	4.83	6.5	ARG	231	12.69	12.5
HIS	31	6.77	6.5	ARG	232	12.42	12.5
HIS	50	6.25	6.5	ARG	235	12.45	12.5
HIS	83	6.77	6.5				

Table S4. Missing force field parameters for the styrene (STR) generated using GAFF with the AM1-BCC partial atomic charges.

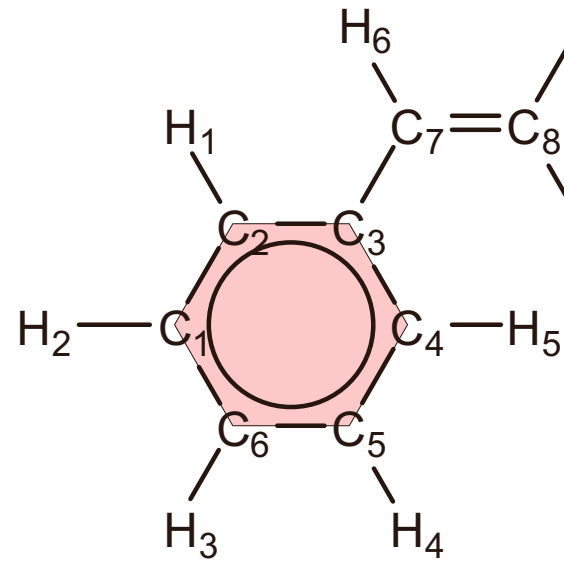

Atom name	Atom type	Atomic charge	Atom name	Atom type	Atomic charge
C4	ca	-0.1185	C2	ca	-0.1185
H5	ha	0.1315	H1	ha	0.1315
C5	ca	-0.1310	C3	ca	-0.0578
H4	ha	0.1310	C7	ce	-0.1132
C6	ca	-0.1270	H6	ha	0.1230
H3	ha	0.1310	C8	c2	-0.2090
C1	ca	-0.1310	H7	ha	0.1135
H2	ha	0.1310	H8	ha	0.1135
FF Parameters					
BOND			ANGLE		
ca-ha	345.8	1.086	ca-ca-ha	48.2	119.88
ca-ca	461.1	1.398	ca-ca-ca	66.6	120.02
ca-ce	361.3	1.476	ca-ca-ce	64.5	120.82
ce-ha	342.5	1.088	ca-ce-ha	47	115.13
c2-ce	547.3	1.346	c2-ce-ca	65.3	121.78
c2-ha	343.1	1.088	ce-c2-ha	49.5	120.45
			c2-ce-ha	49.6	119.94
			ha-c2-ha	38.1	116.9

DIHEDRAL									
ca-ca-ca-ha	4	14.5000	180	2	ce-ca-ca-ha	4	14.5	180	2
ca-ca-ca-ca	4	14.5000	180	2	ca-ca-ca-ce	4	14.5	180	2
ca-ca-ce-ha	4	2.8000	180	2	ha-c2-ce-ca	4	26.6	180	2
ca-ca-ce-c2	4	2.8000	180	2	ha-c2-ce-ha	4	26.6	180	2
ha-ca-ca-ha	4	14.5000	180	2					

IMPROPER				NONBON		
ca-ca-ca-ha	1.1	180	2	ca	1.908	0.086
ca-ca-ca-ce	1.1	180	2	ha	1.459	0.015
c2-ca-ce-ha	1.1	180	2	ce	1.908	0.086
ce-ha-c2-ha	1.1	180	2	c2	1.908	0.086

SUPPLEMENTAL DATA S1: Oxyfunctionalization Chromatograms

1) Oxidation of Styrene by AckBar

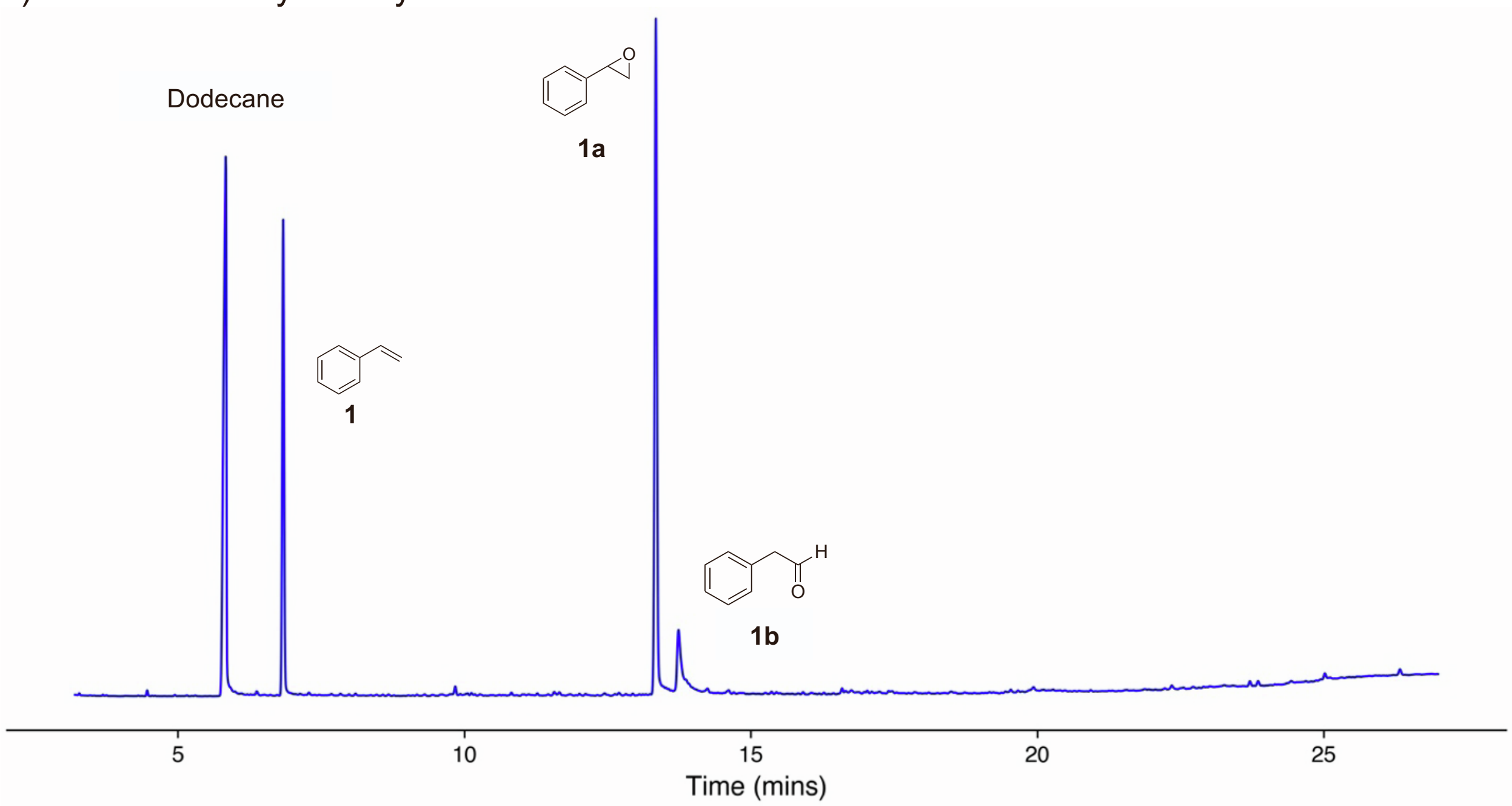

Reaction of styrene (**1**) with H_2O_2 catalyzed by AckBar to produce styrene oxide (**1a**) and phenylacetaldehyde (**1b**). Dodecane added as internal standard.

2) Oxidation of 4-Chlorostyrene by AckBar

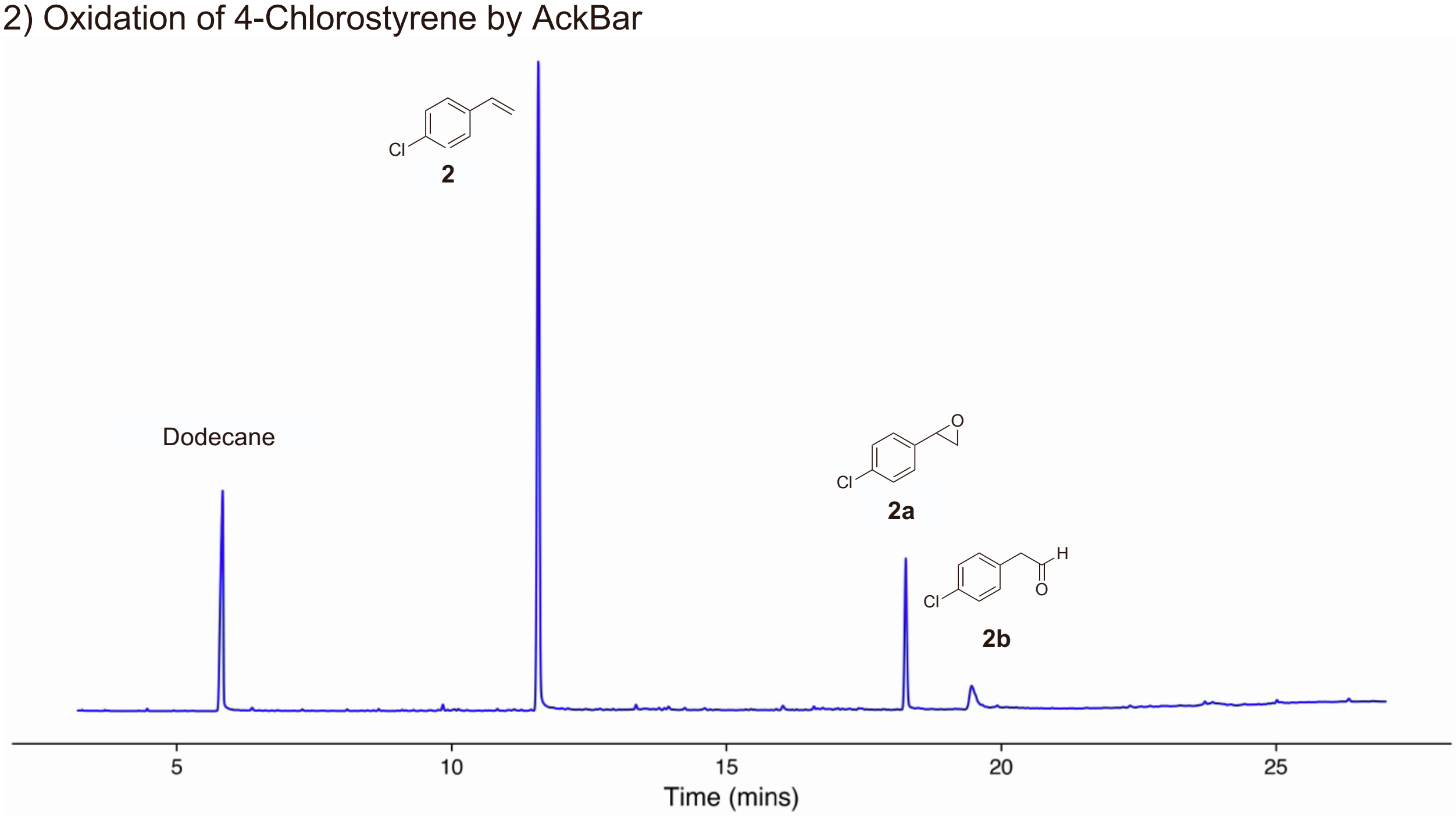

Reaction of 4-chlorostyrene (**2**) with H_2O_2 catalyzed by AckBar to produce 4-chlorostyrene oxide (**2a**) and 4-chlorophenylacetaldehyde (**2b**). Dodecane added as internal standard.

3) Oxidation of α-methylstyrene by AckBar

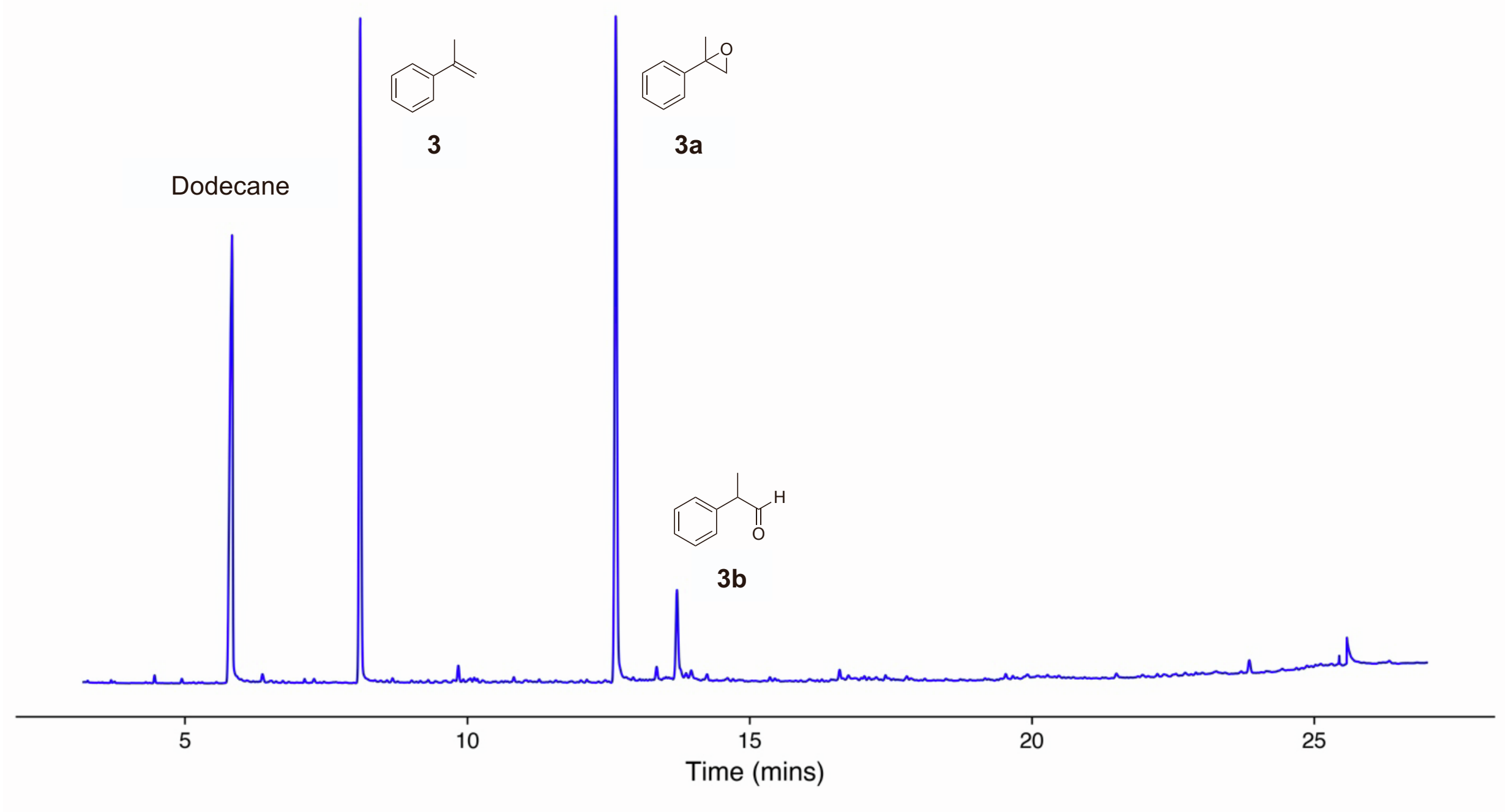

Reaction of α-methylstyrene (**3**) with H_2O_2 catalyzed by AckBar to produce 1-methyl-1-phenyloxirane (**3a**) and 2-phenylpropanaldehyde (**3b**). Dodecane added as internal standard.

4) Oxidation of ethylbenzene by AckBar

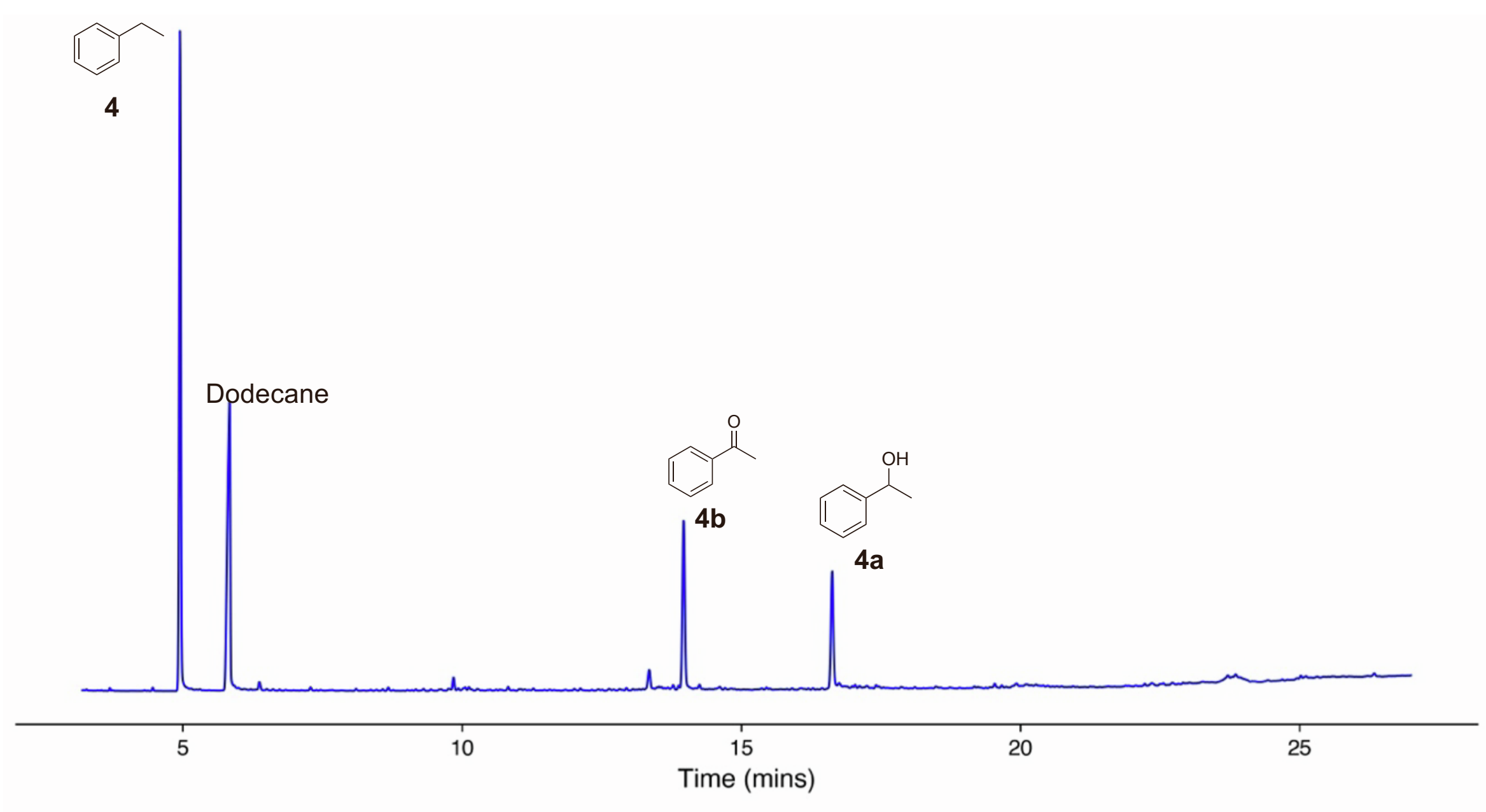

Reaction of ethylbenzene (**4**) with H_2O_2 catalyzed by AckBar to produce 1-phenylethanol (**4a**) and acetophenone (**4b**). Dodecane added as internal standard.

5) Oxidation of tetrahydronaphthalene by AckBar

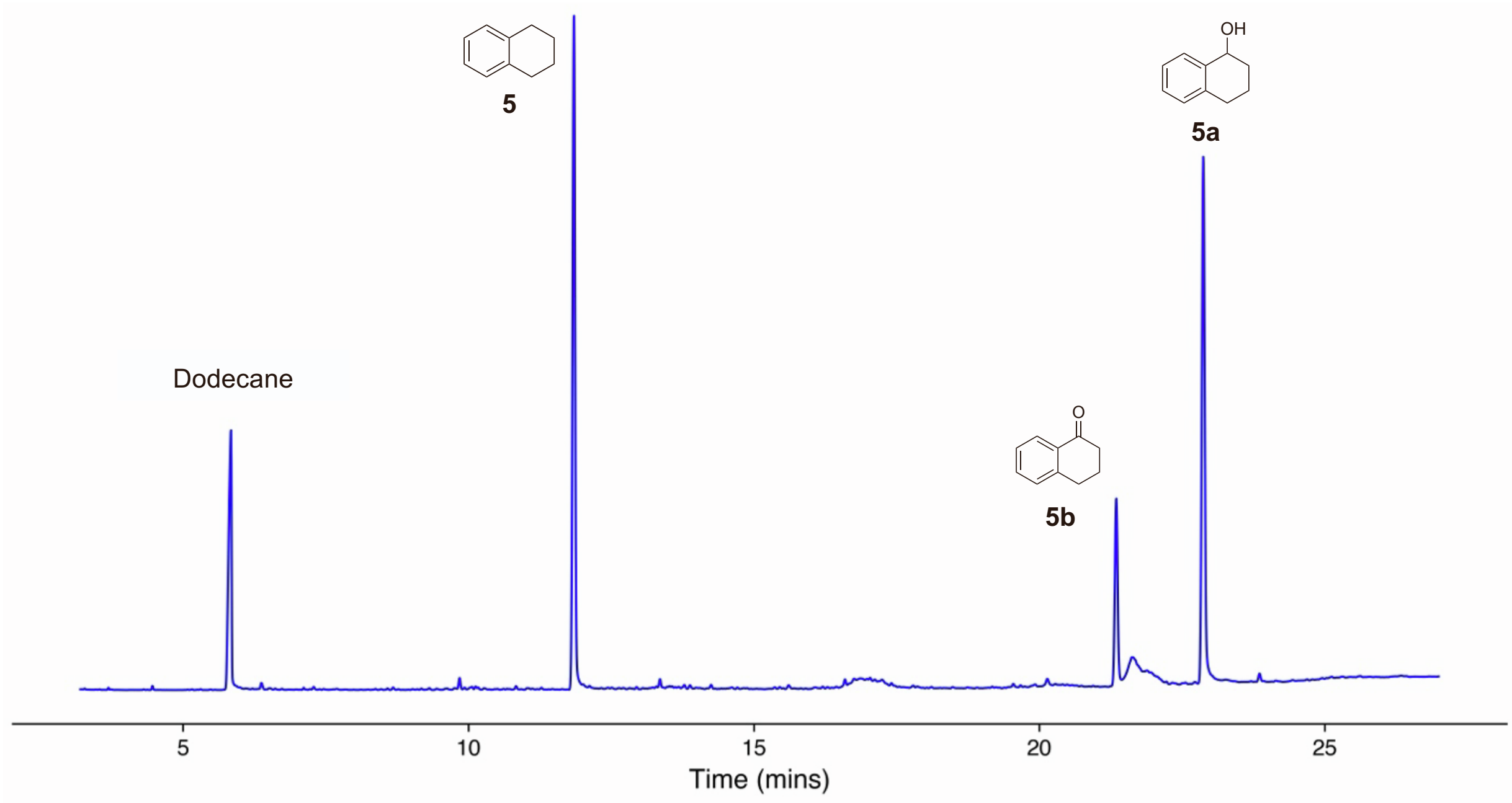

Reaction of tetrahydronaphthalene (**5**) with H_2O_2 catalyzed by AckBar to produce α-tetralol (**5a**) and α-tetralone (**5b**). Dodecane added as internal standard.

6) Oxidation of isophorone by AckBar

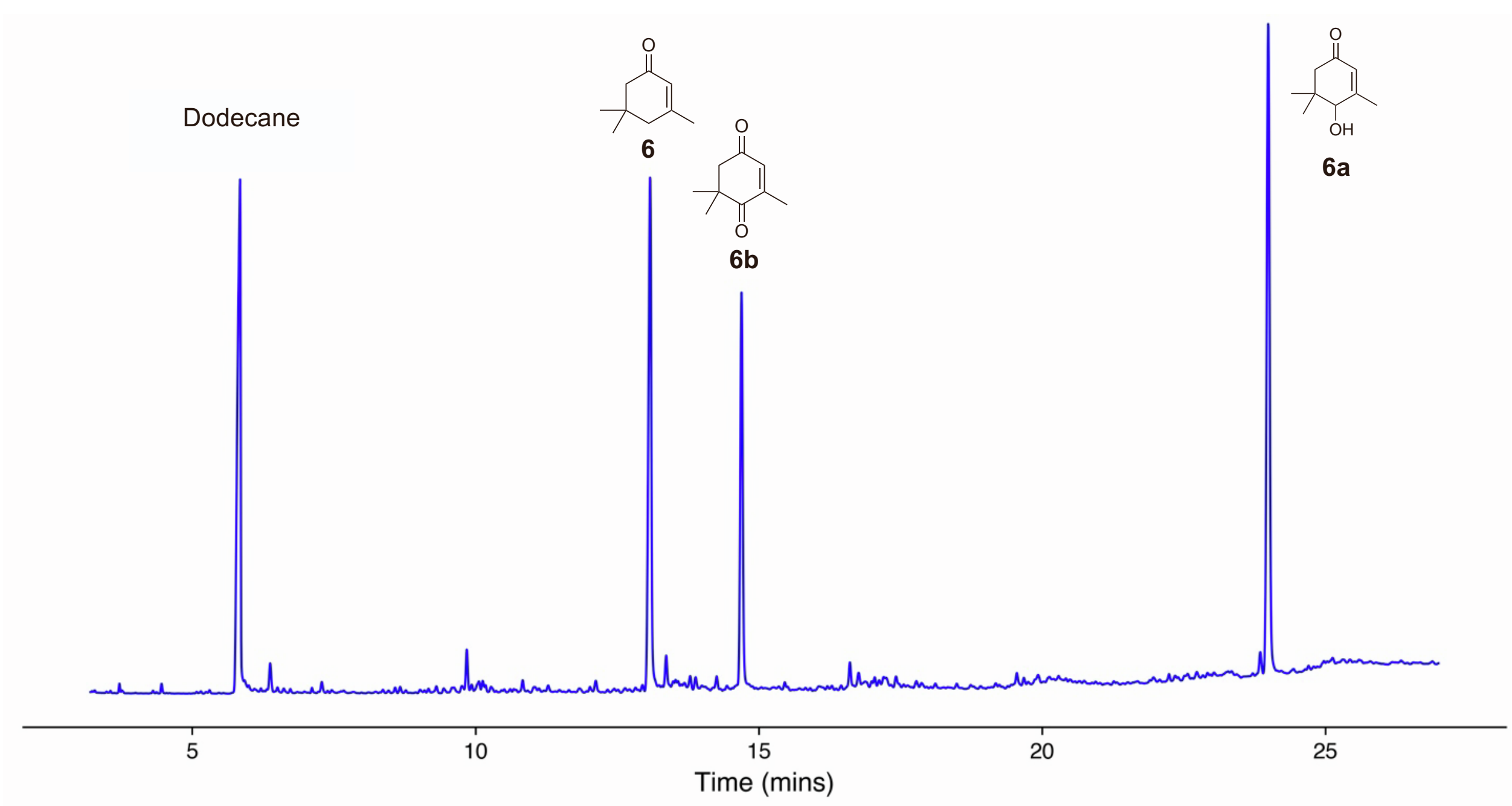

Reaction of isophorone (**6**) with H_2O_2 catalyzed by AckBar to produce 4-hydroxyisophorone (**6a**) and 4-ketoisophorone (**6b**). Dodecane added as internal standard.

7) Oxidation of (+)-α-Pinene by AckBar

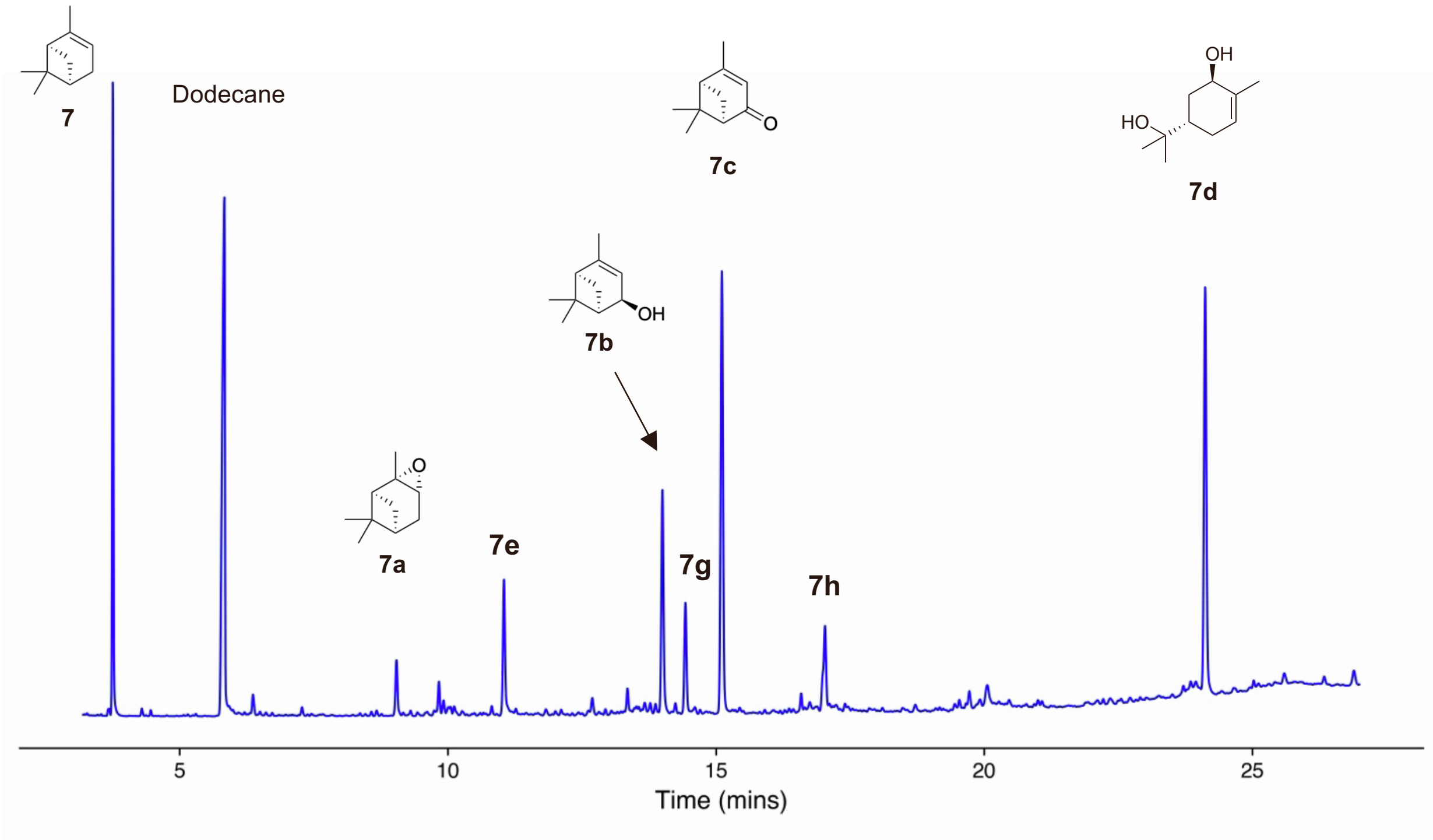

Reaction of (+)-α-pinene (**7**) with H_2O_2 catalyzed by AckBar to produce α-pinene oxide (**7a**) and (*R*)-*cis*-verbenol (**7b**), (*R*)-verbenone (**7c**), and *trans*-sobrerol (**7d**). Dodecane added as internal standard. Other side product peaks (**7e**, **7g** and **7h**) did not match known compounds (myrtenol, myrtenal) and were not be identified.

Mass spectra of highlighted peaks:

7a

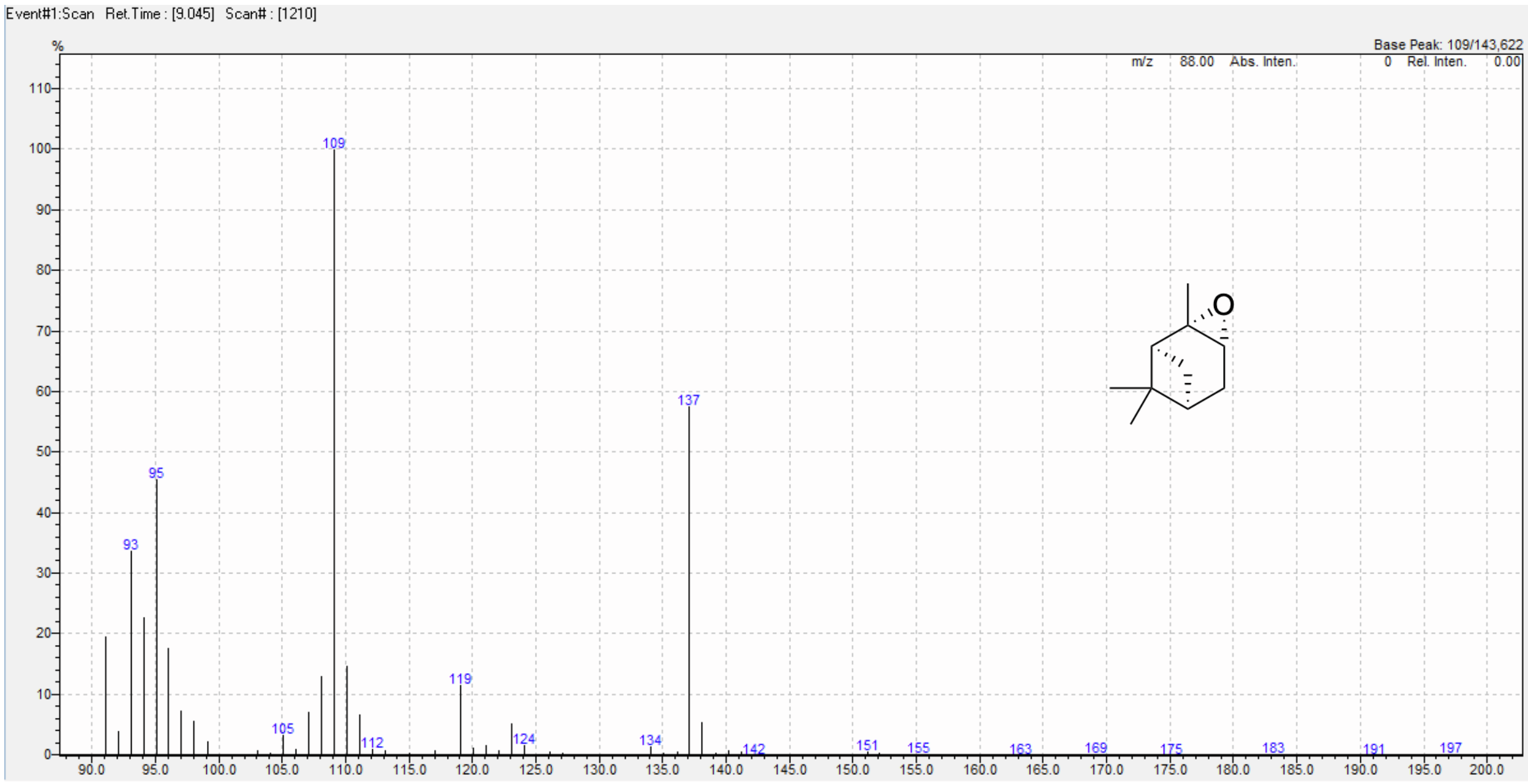

7b

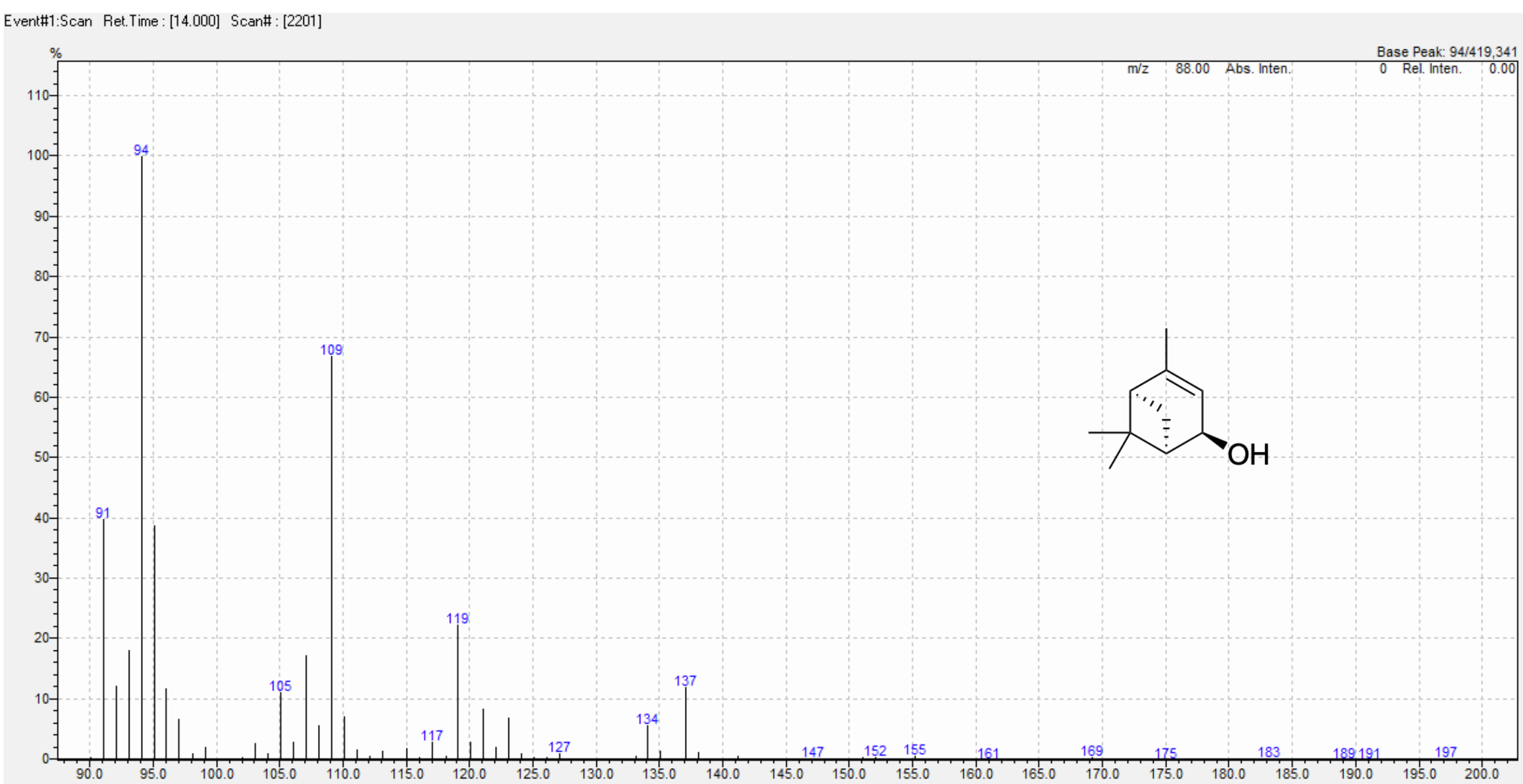

7c

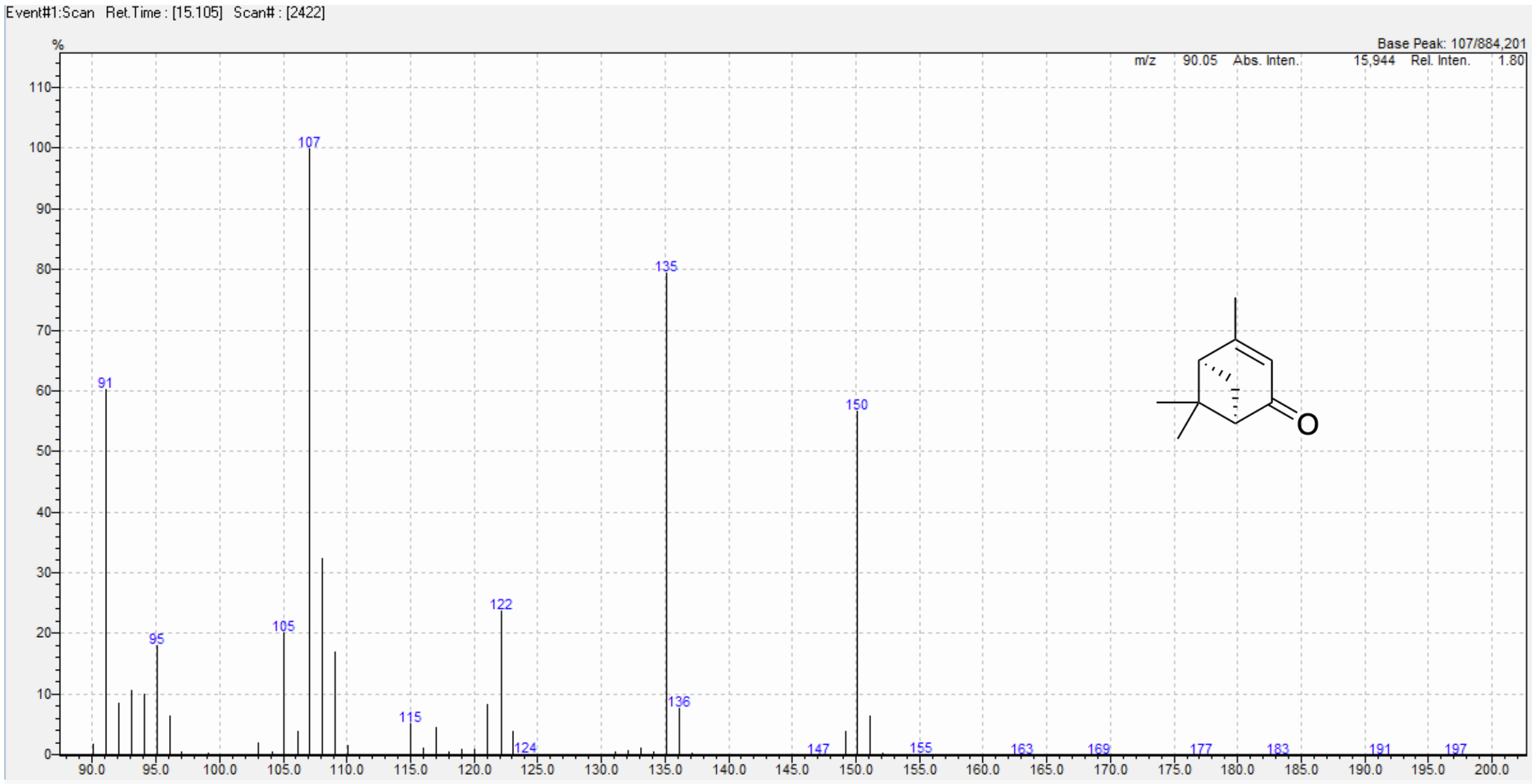
Event#1:Scan Ret.Time : [15.105] Scan# : [2422]
Base Peak: 107/884,201
m/z 90.05 Abs. Inten. 15,944 Rel. Inten. 1.80
O

7d

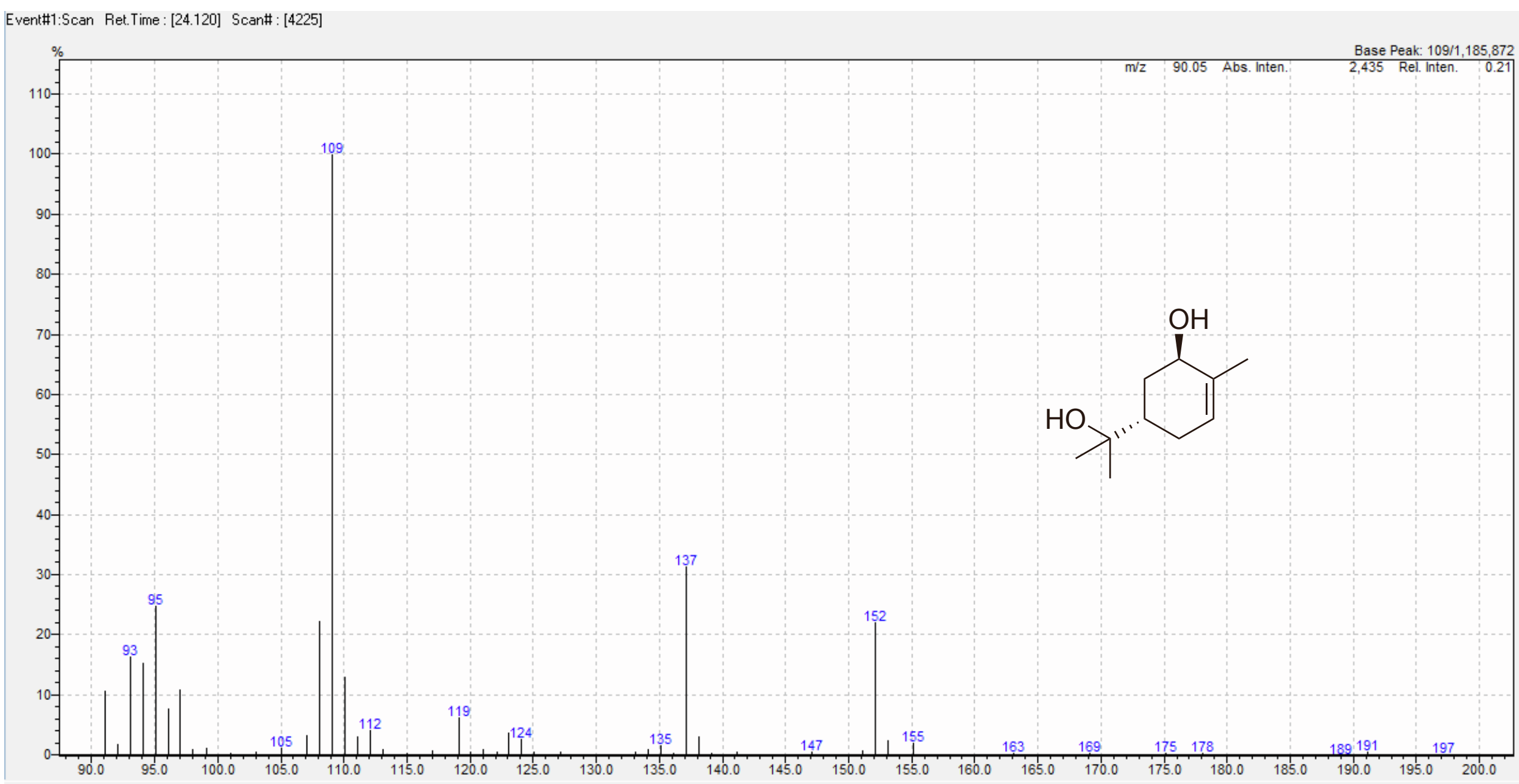
Event#1:Scan Ret.Time : [24.120] Scan# : [4225]
Base Peak: 109/1,185,872
m/z 90.05 Abs. Inten. 2,435 Rel. Inten. 0.21
OH
HO

7e

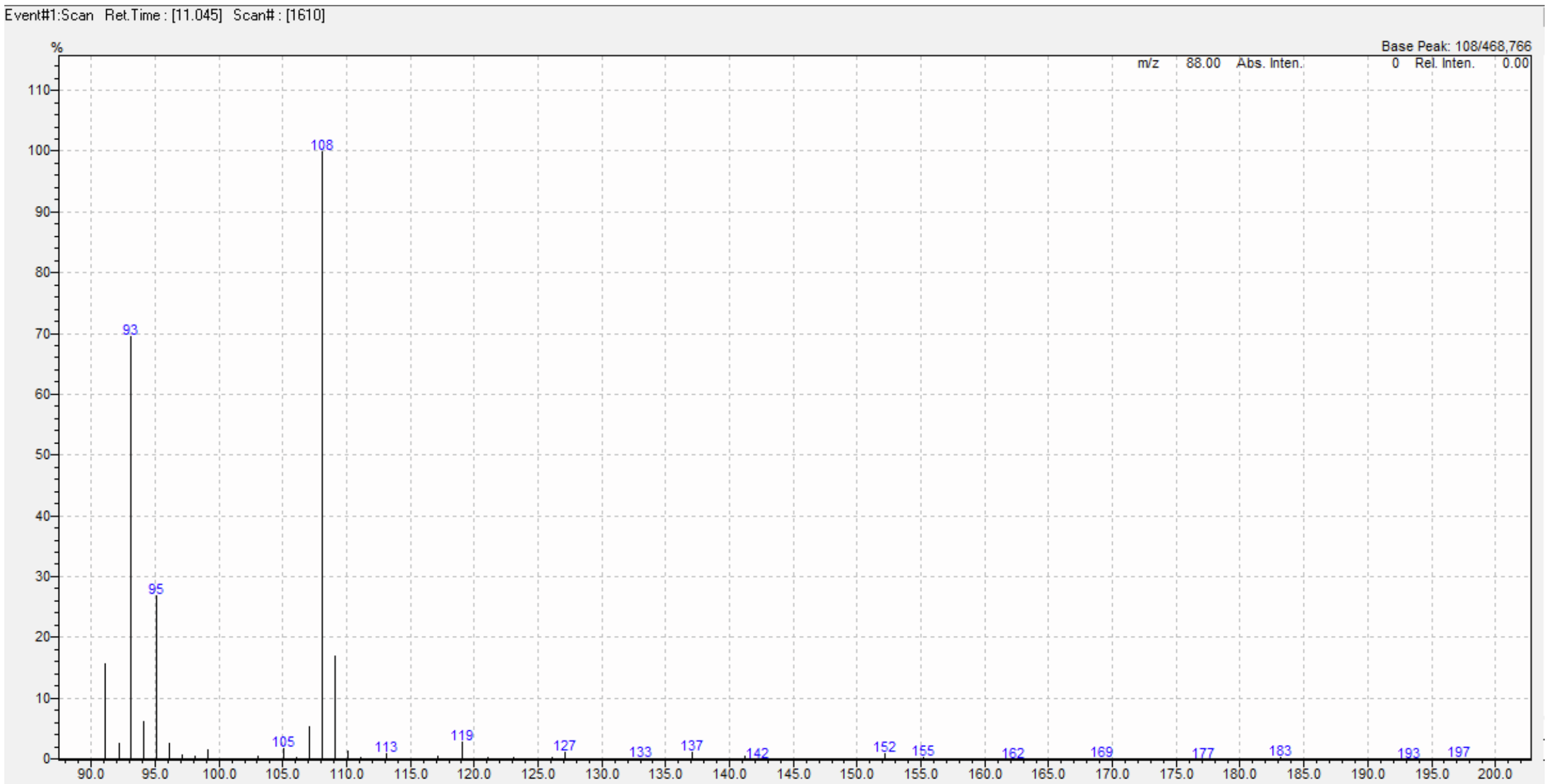

Event#1:Scan Ret.Time : [11.045] Scan# : [1610]
Base Peak: 108/468,766
m/z 88.00 Abs. Inten. 0 Rel. Inten. 0.00
%
108
93
95
105
113
119
127
133
137
142
152
155
162
169
177
183
193
197

7g

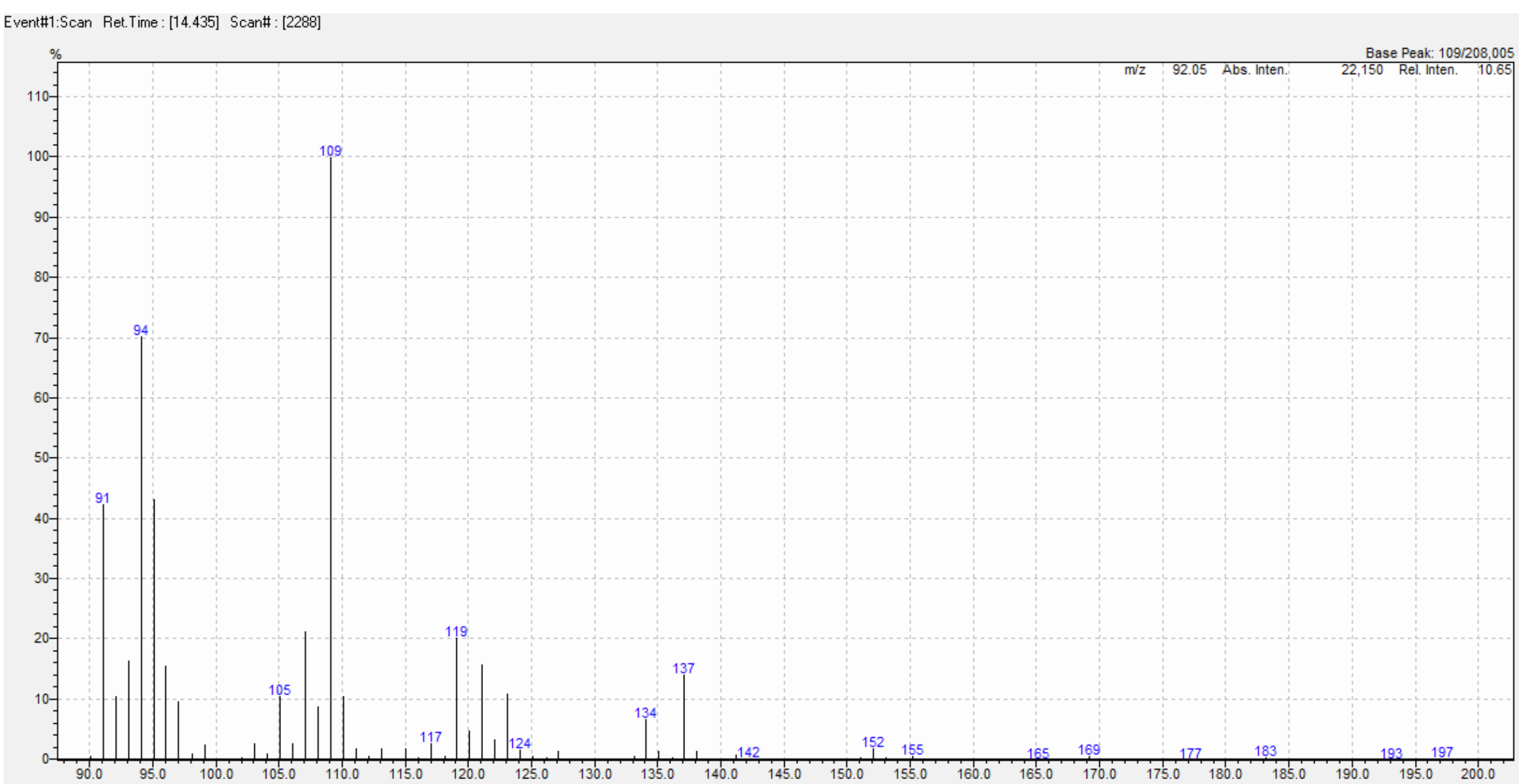

Event#1:Scan Ret.Time : [14.435] Scan# : [2288]
Base Peak: 109/208,005
m/z 92.05 Abs. Inten. 22,150 Rel. Inten. 10.65
%
109
94
91
119
137
105
134
117
124
142
152
155
165
169
177
183
193
197

7h

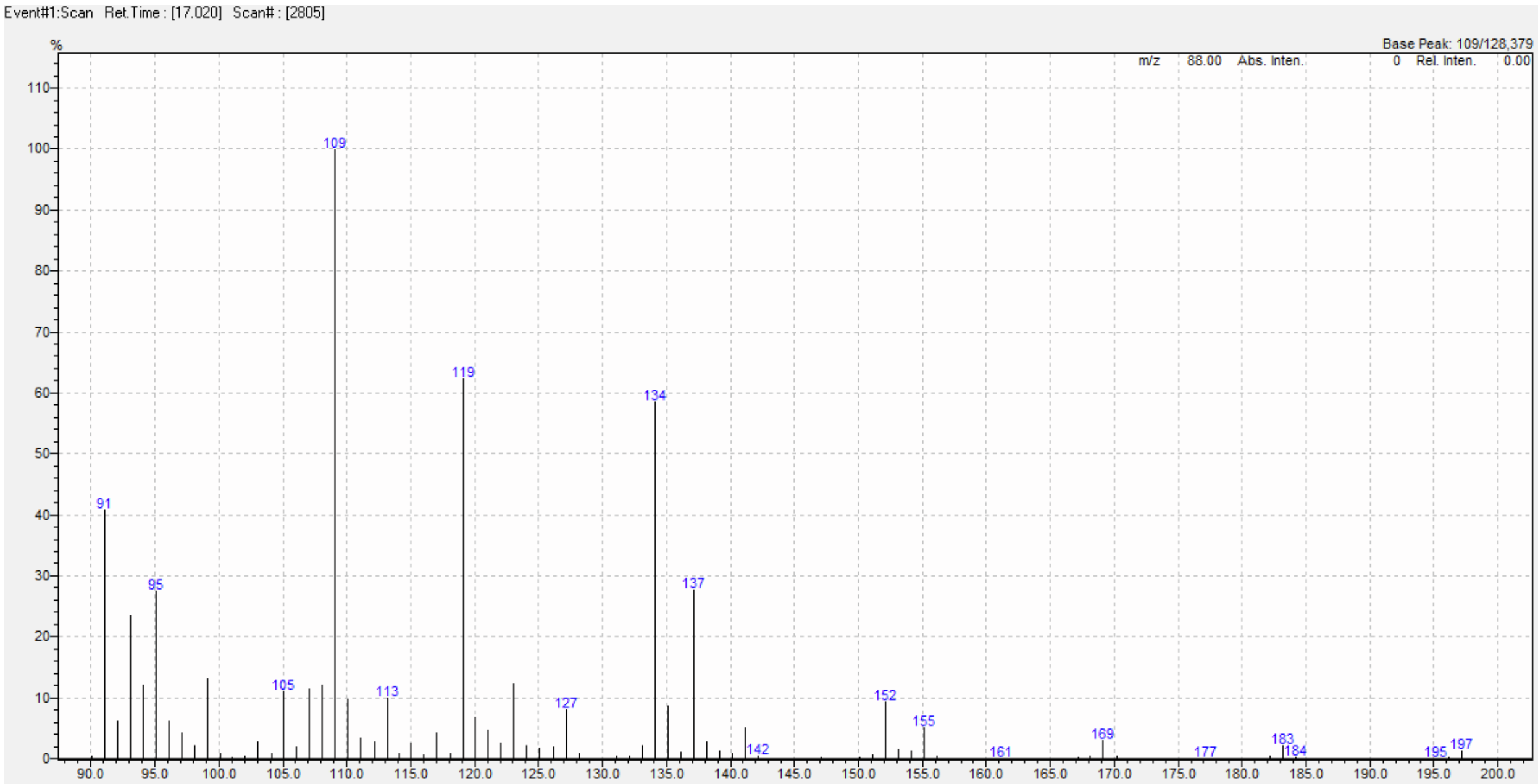
Event#1:Scan Ret.Time : [17.020] Scan# : [2805]
Base Peak: 109/128,379
m/z 88.00 Abs. Inten. 0 Rel. Inten. 0.00
%
91
95
105
109
113
119
127
134
137
142
152
155
161
169
177
183
184
195
197

8) Oxidation of (*R*)- and (*S*)-limonene by AckBar

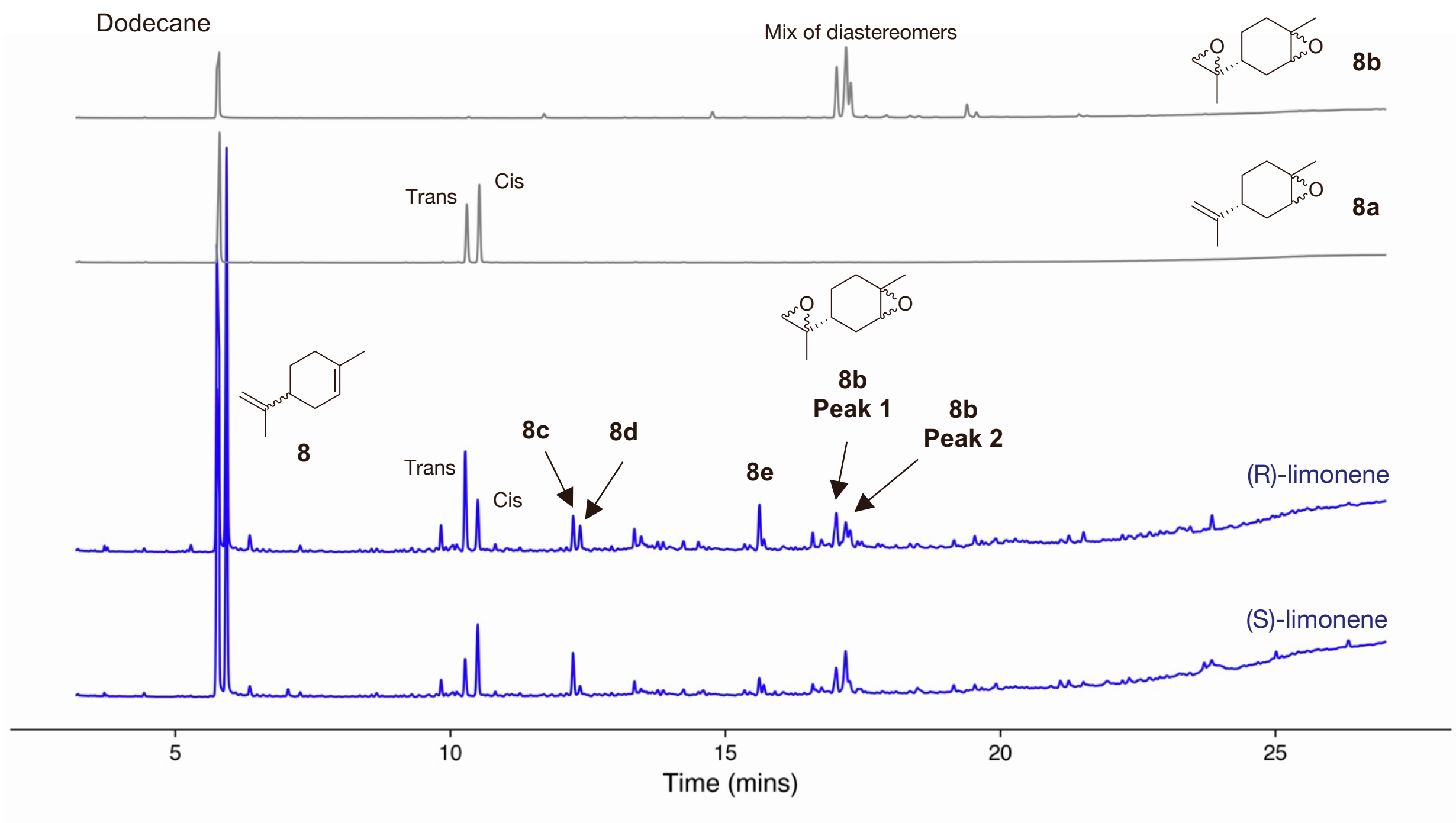

Reaction of (*R*)-limonene (blue trace, top) or (*S*)-limonene (blue trace, bottom) with H_2O_2 catalyzed by AckBar to produce limonene oxide (**8a**) and limonene diepoxide (**8b**) as a mixture of diastereomers. Dodecane added as internal standard. Shown in grey traces above are the authentic standards: (*R*)-limonene diepoxide (grey, top, as a mixture of diastereomers) and (*R*)-limonene oxide (grey, bottom, both *trans*- and *cis*-epoxides). While other side product peaks (**8c**, **8d** and **8e**) contained mass spectra indicative of limonene oxidation, the peaks did not align with authentic standards of known hydroxylation products, such as perillyl alcohol, carveol or carvone.

Mass spectra of highlighted peaks:

***trans*-8a**

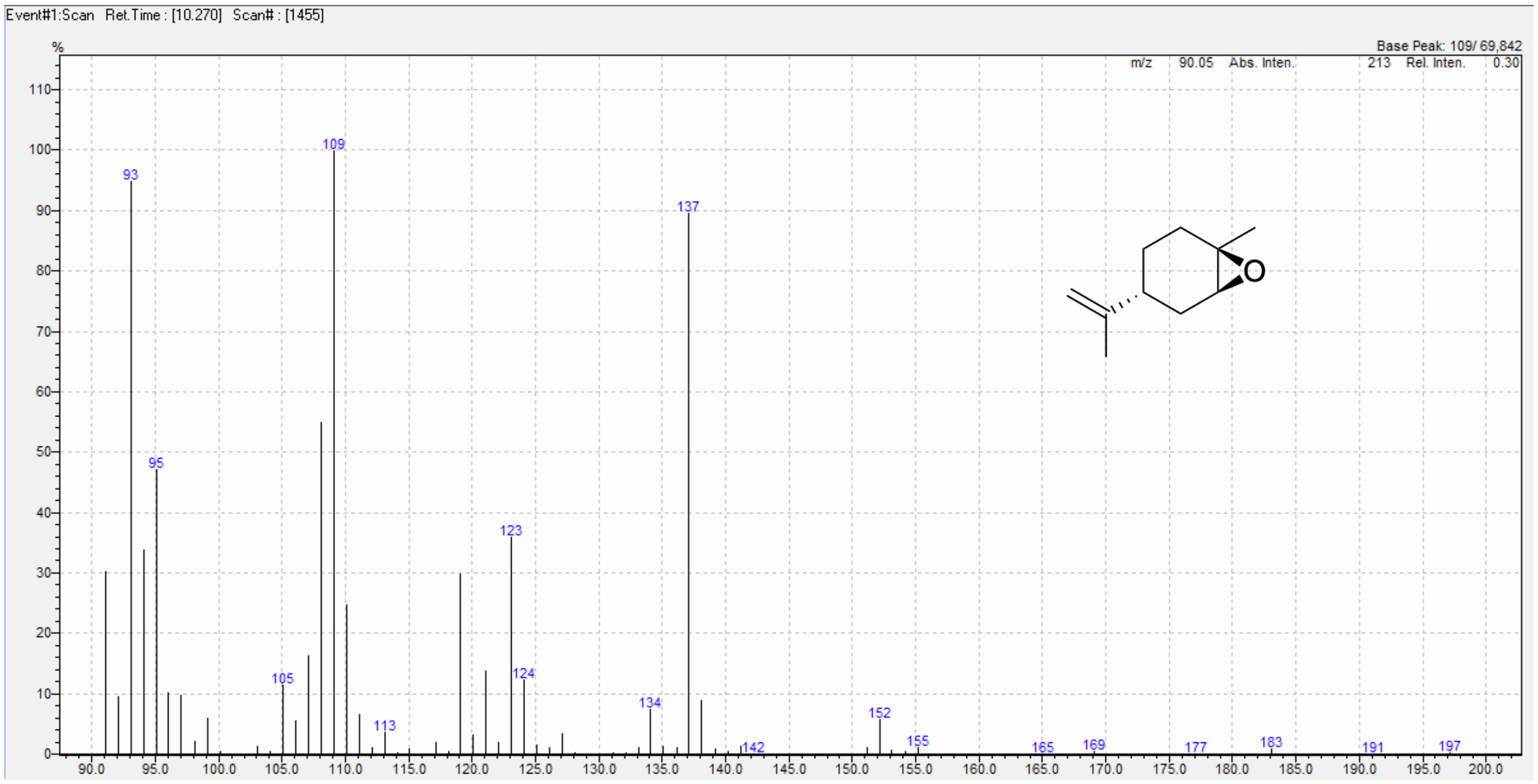

***cis*-8a**

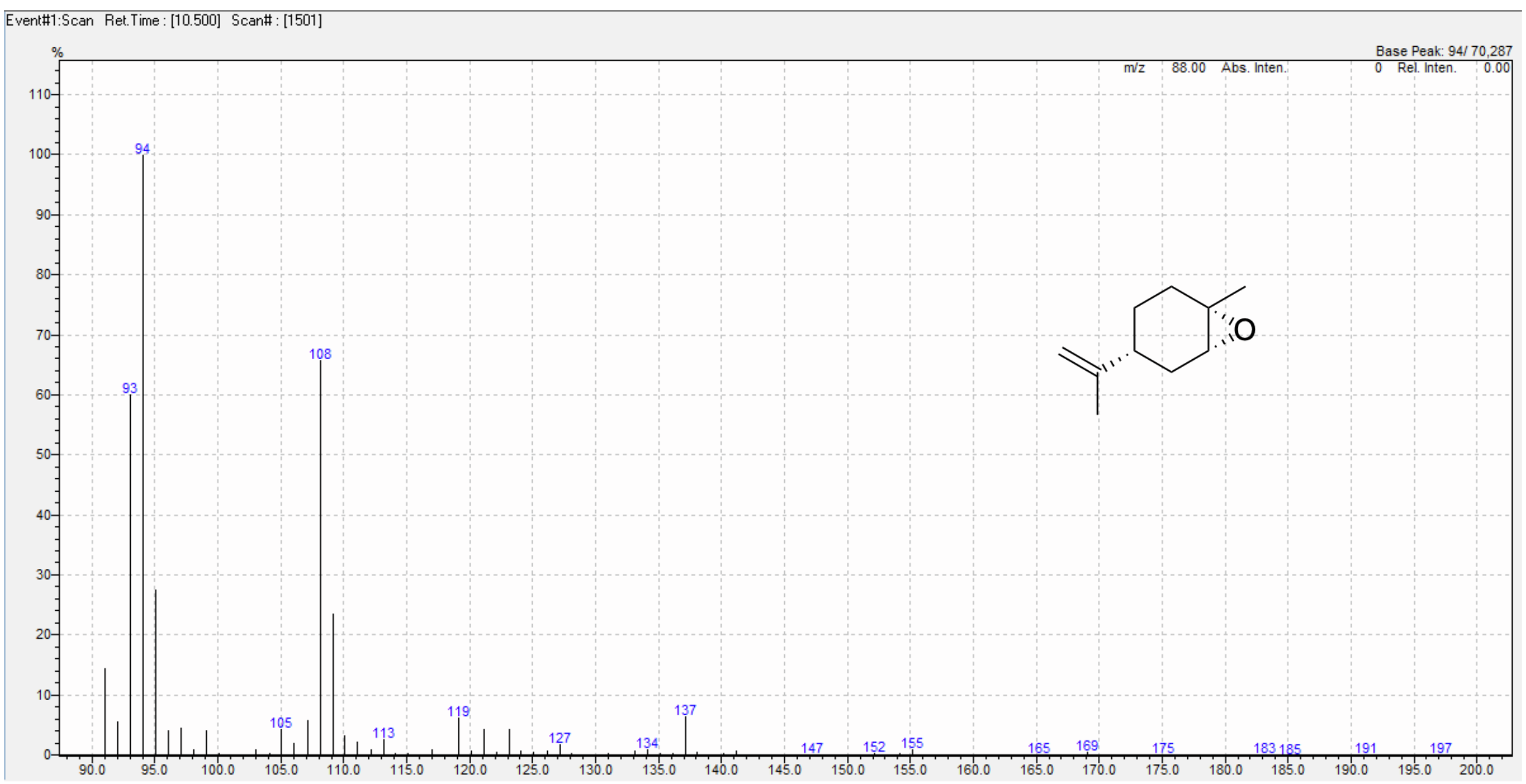

8b Peak 1

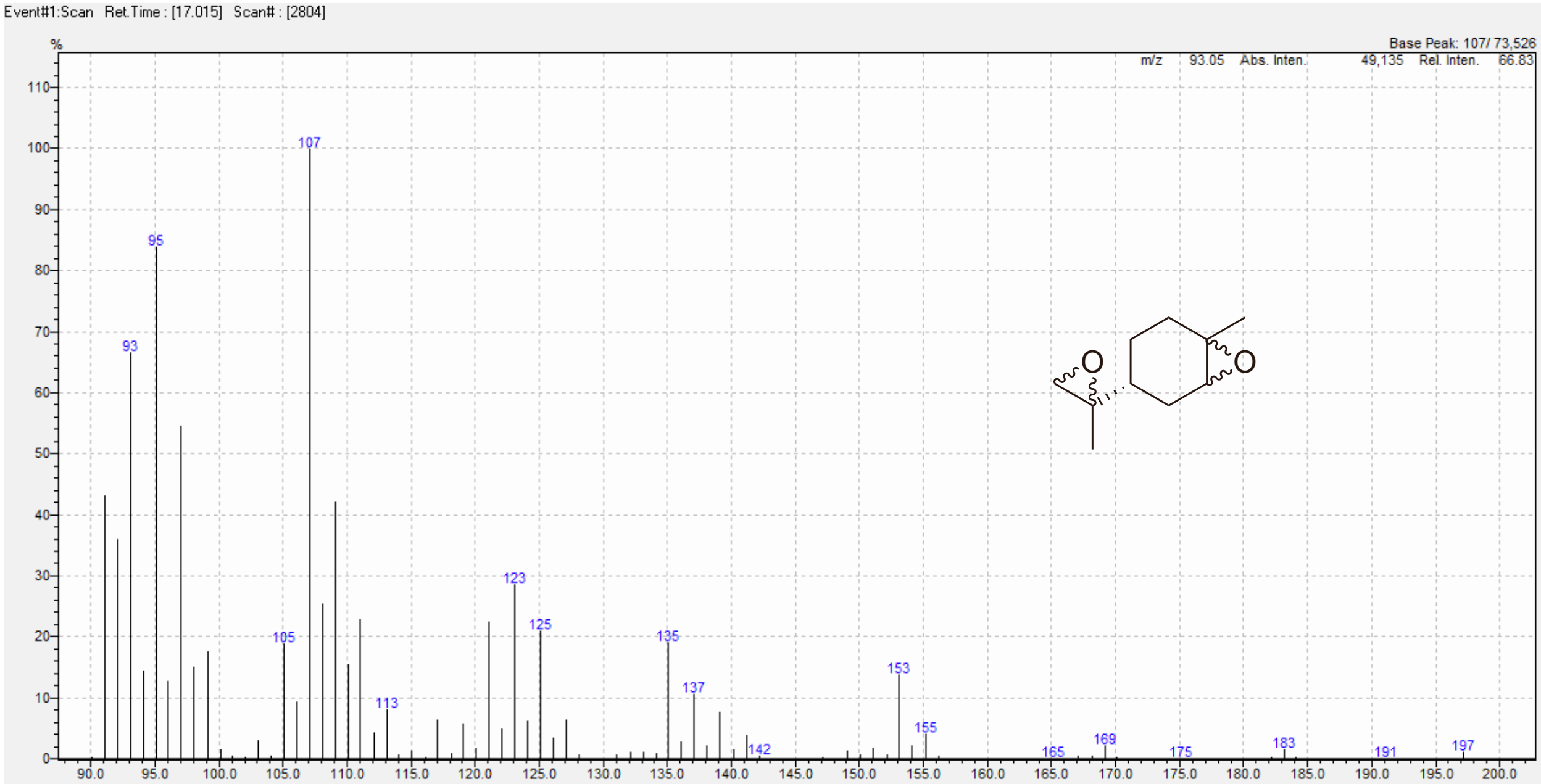

8b Peak 2

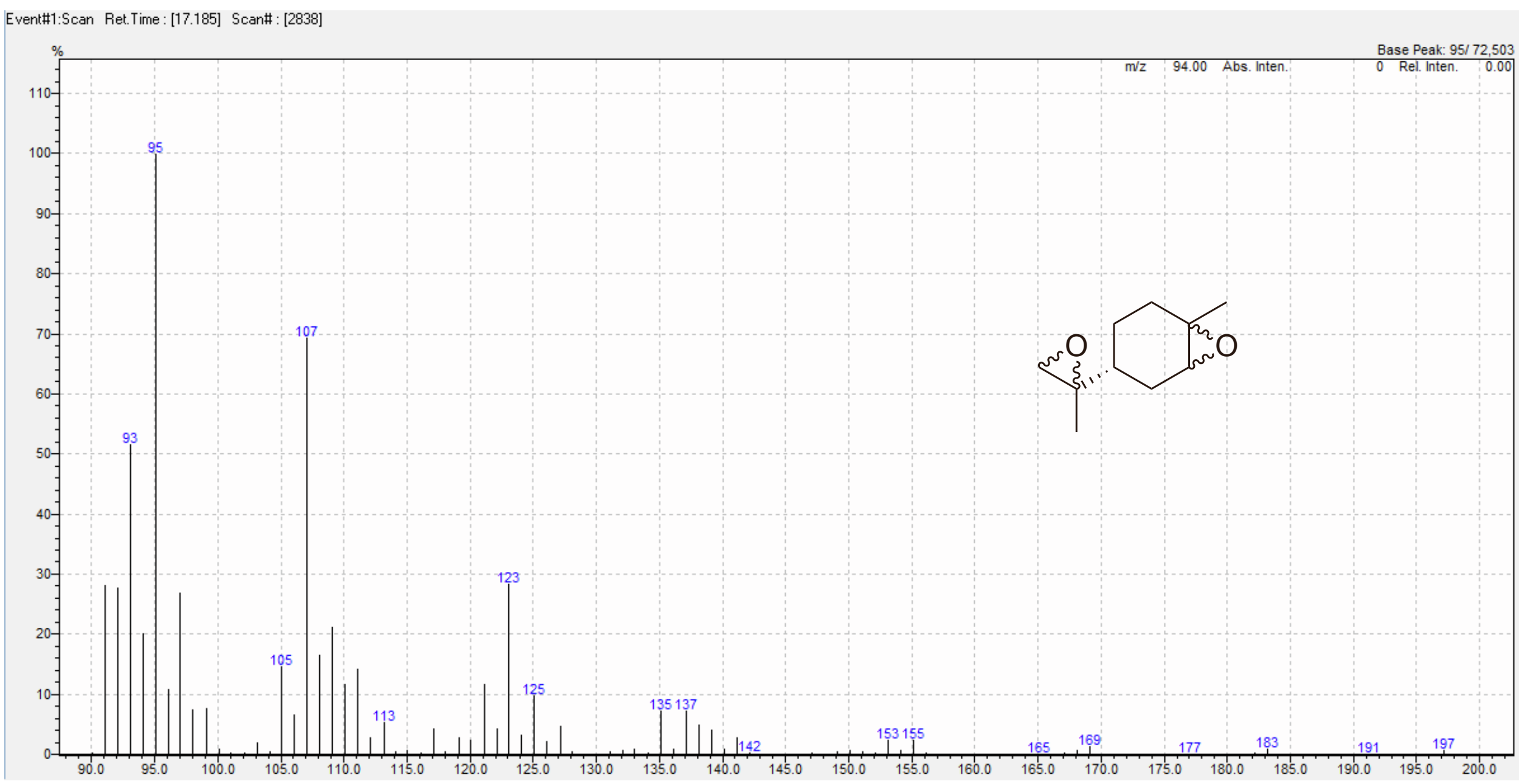

8c

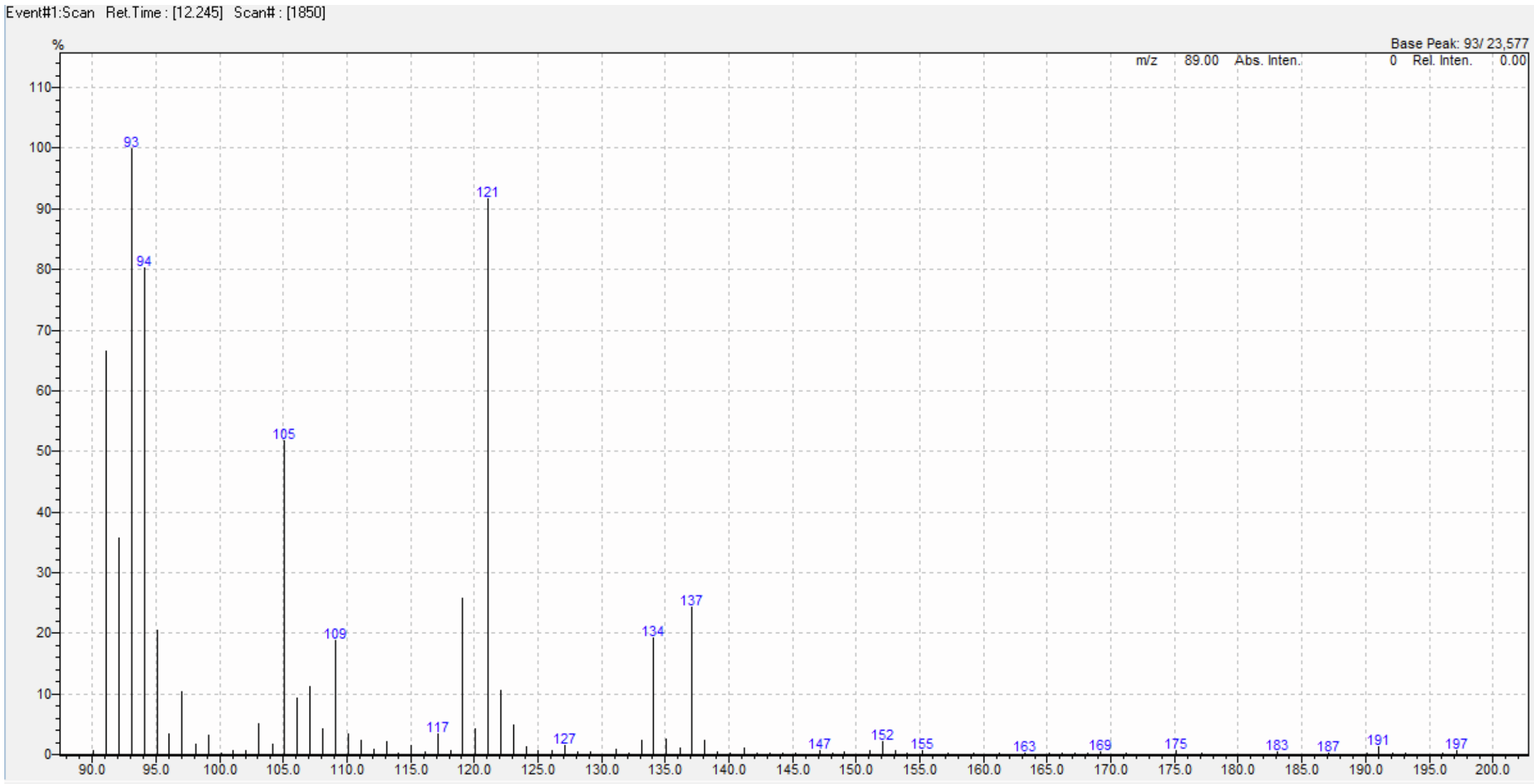
Event#1:Scan Ret.Time : [12.245] Scan# : [1850]
Base Peak: 93/ 23,577
m/z 89.00 Abs. Inten. 0 Rel. Inten. 0.00
%
93
94
105
121
109
117
127
134
137
147
152
155
163
169
175
183
187
191
197

8d

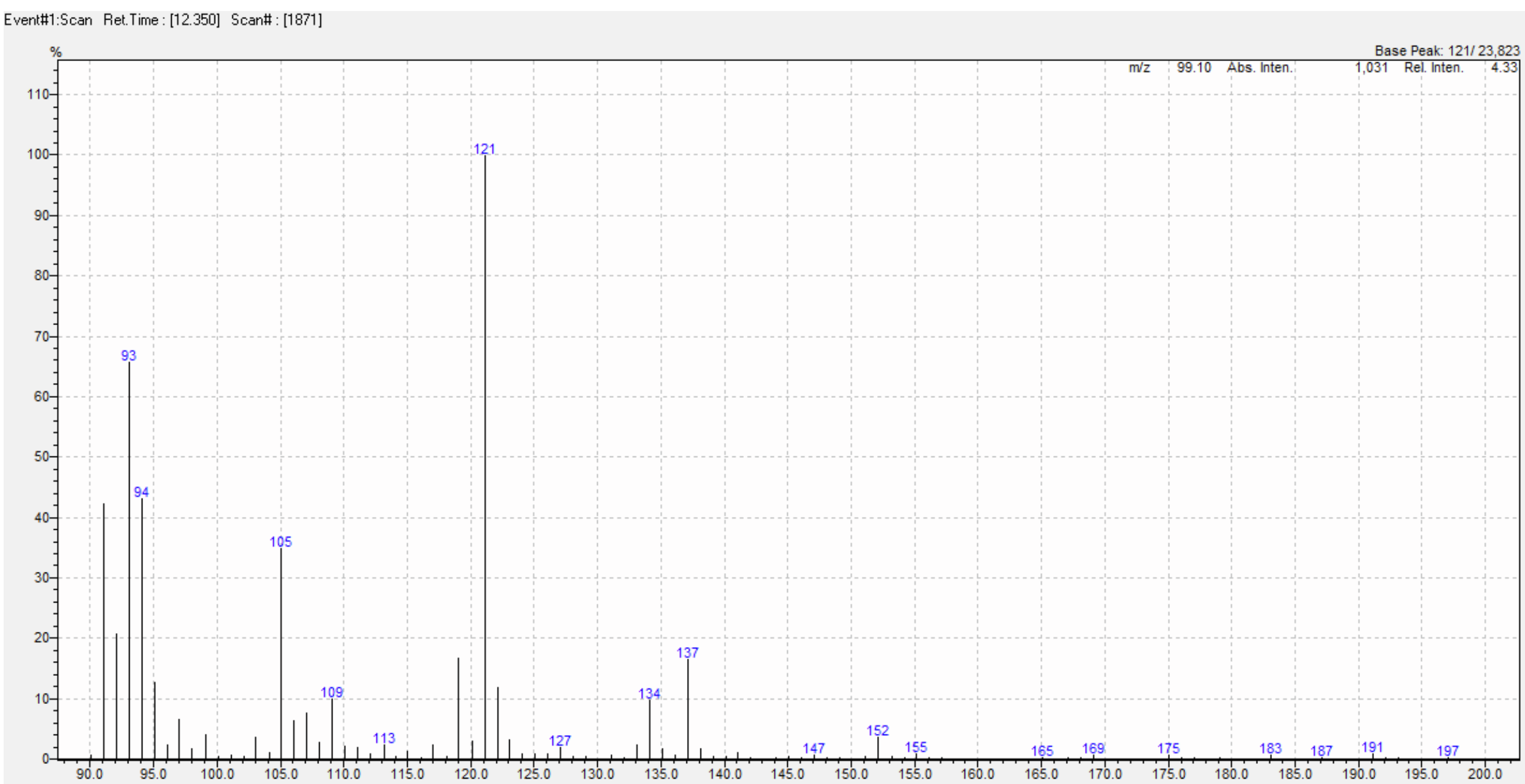
Event#1:Scan Ret.Time : [12.350] Scan# : [1871]
Base Peak: 121/ 23,823
m/z 99.10 Abs. Inten. 1,031 Rel. Inten. 4.33
%
121
93
94
105
109
113
127
134
137
147
152
155
165
169
175
183
187
191
197

8e

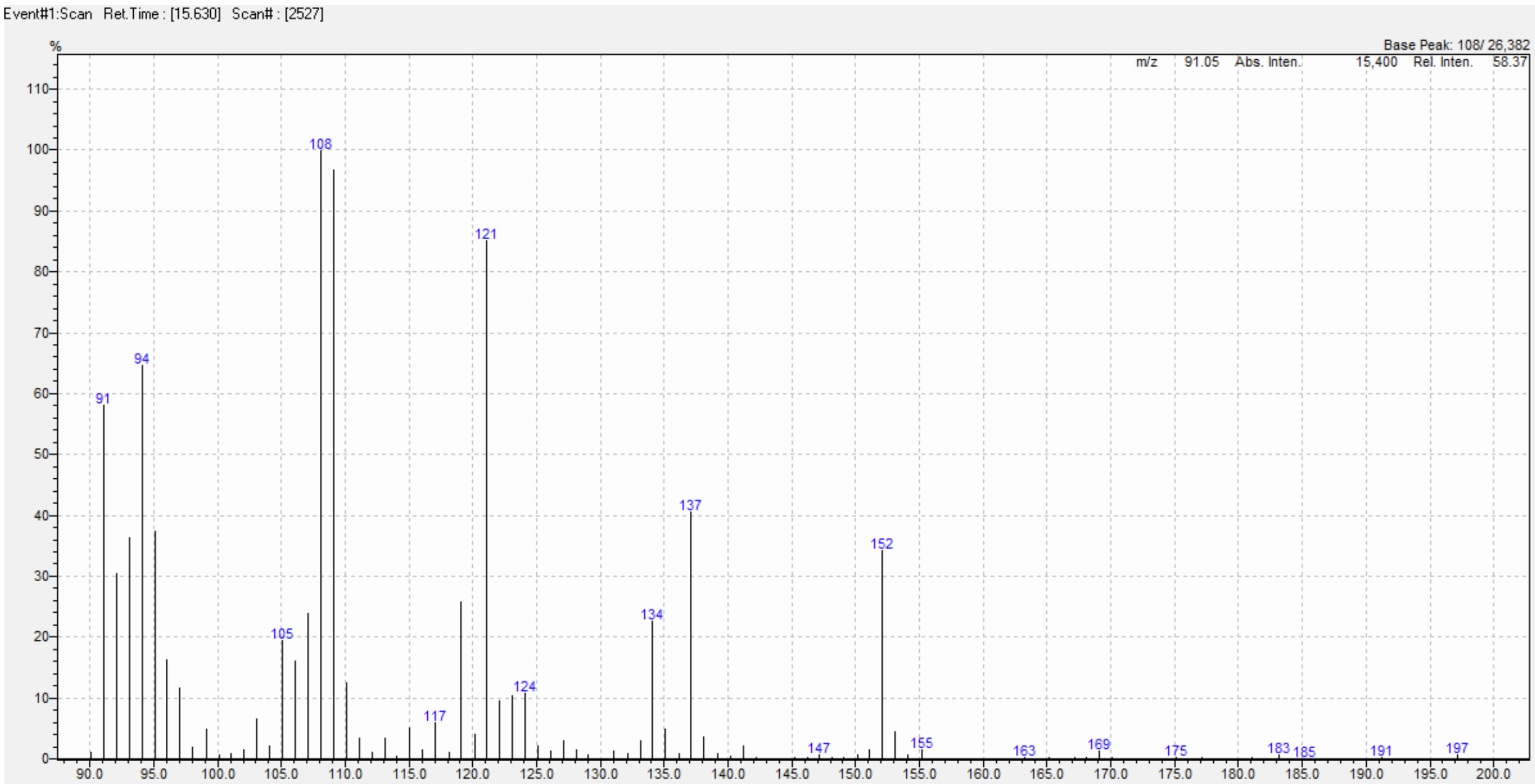
Event#1:Scan Ret.Time : [15.630] Scan# : [2527]
Base Peak: 108/ 26,382
m/z 91.05 Abs. Inten. 15,400 Rel. Inten. 58.37
%
91
94
105
108
117
121
124
134
137
147
152
155
163
169
175
183
185
191
197

Chiral GC/MS for calculation of enantioselectivities

For each chromatogram shown below, the grey chromatograms are HPLC-grade standard compounds and the blue is AckBar unless otherwise labeled.

1) Enantioselectivity of Styrene Oxide Synthesized by AckBar

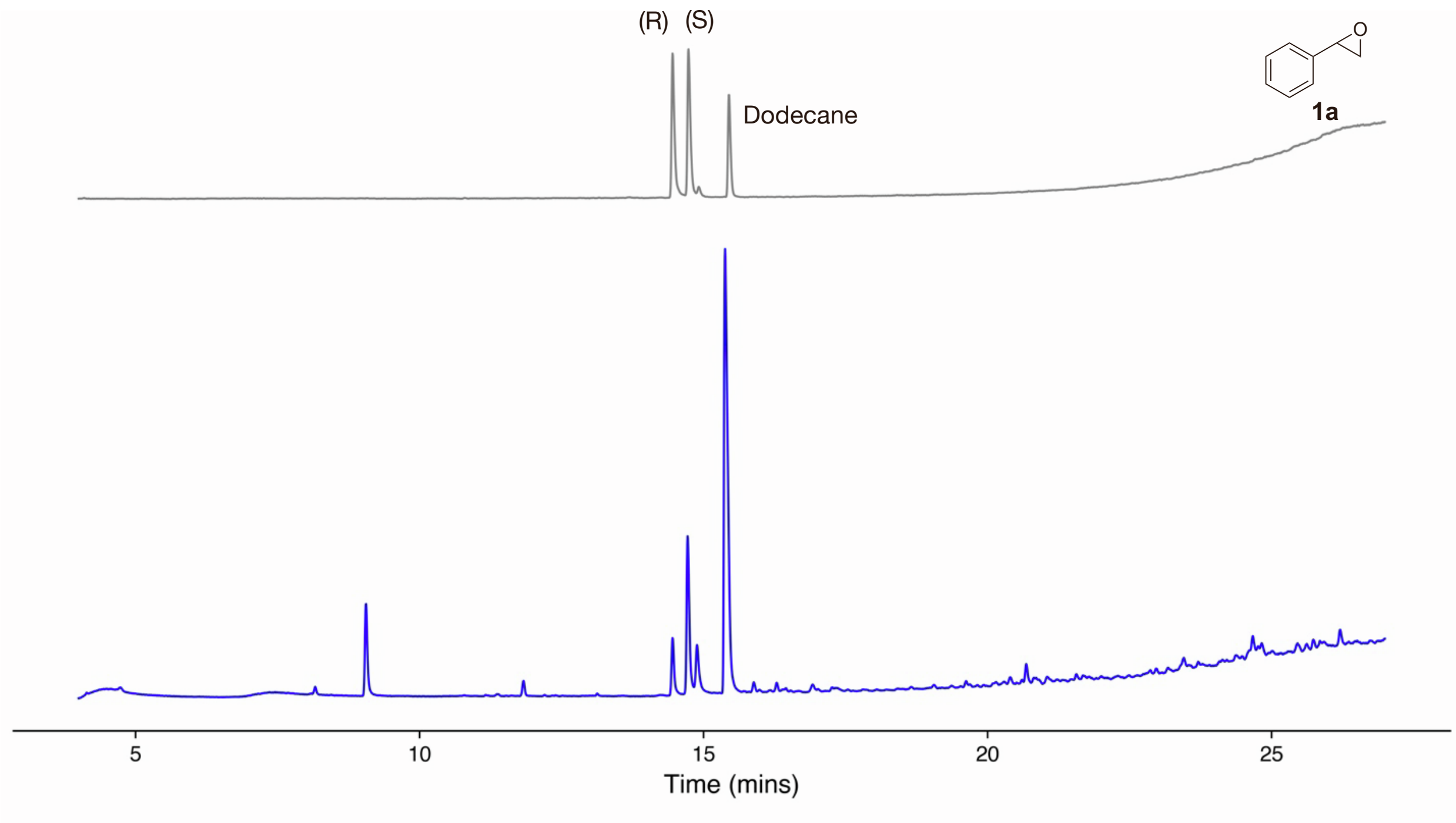

Reaction of styrene (**1**) with H_2O_2 catalyzed by AckBar (blue, bottom) to produce styrene oxide (**1a**) and phenylacetaldehyde (**1b**). The peak that shoulders (***S***)**-1a** is **1b**. On top in grey is the authentic standard with peaks labeled based on previous reports. Dodecane added as internal standard.

2) Enantioselectivity of α-Tetralol Synthesized by AckBar and *Cgl*UPO$_{Pic}$

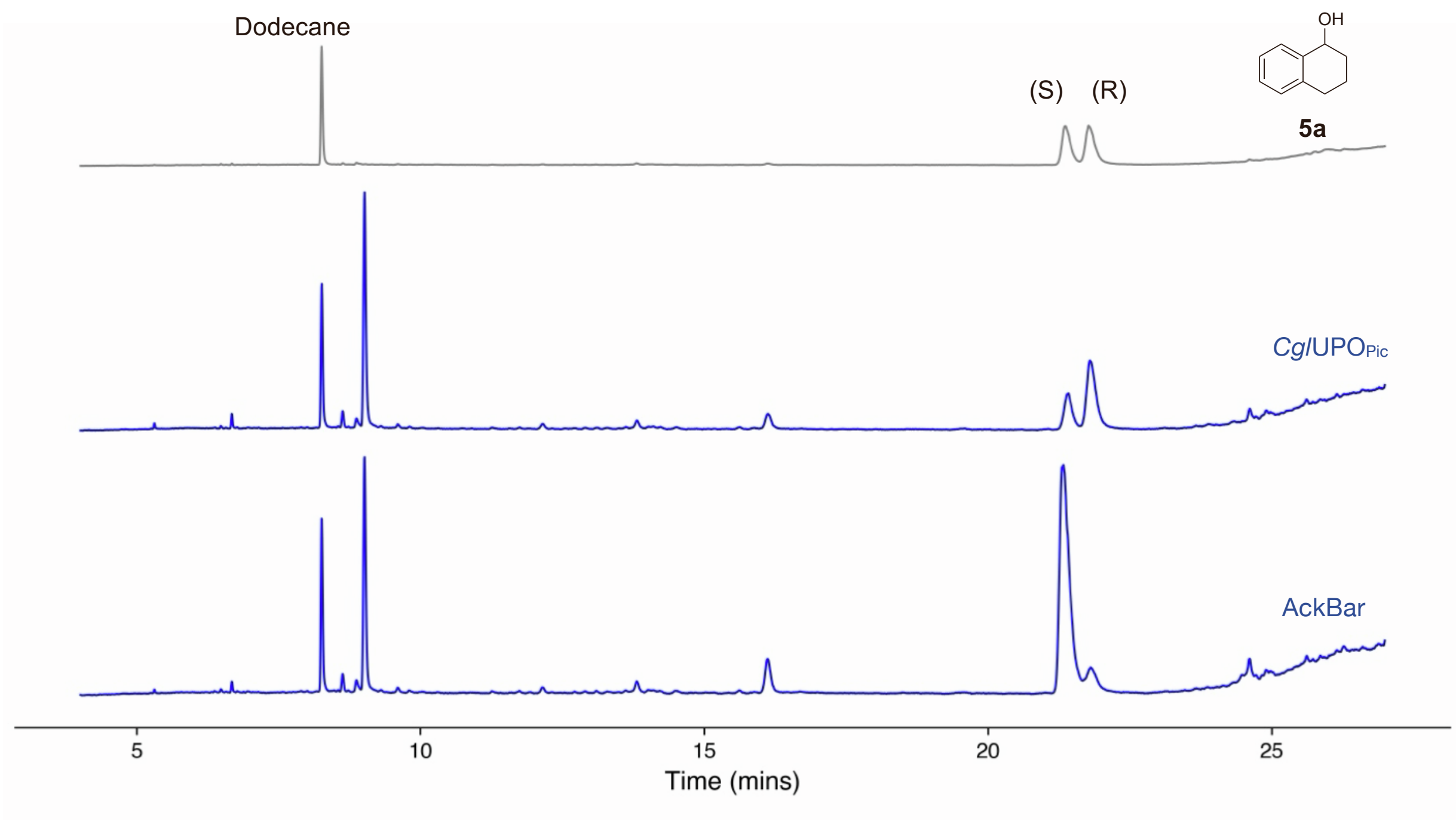

Reaction of tetrahydronaphthalene (**3**) with H_2O_2 catalyzed by *Cgl*UPO$_{Pic}$ (blue, middle) and AckBar (blue, bottom) to produce (*R*)- and (*S*)-α-tetralol (**5a**). On top in grey is the authentic standard with enantiomers labeled based on previous reports. Dodecane added as internal standard.

SUPPLEMENTAL DATA S2: Calibration Curves of Relevant Compounds

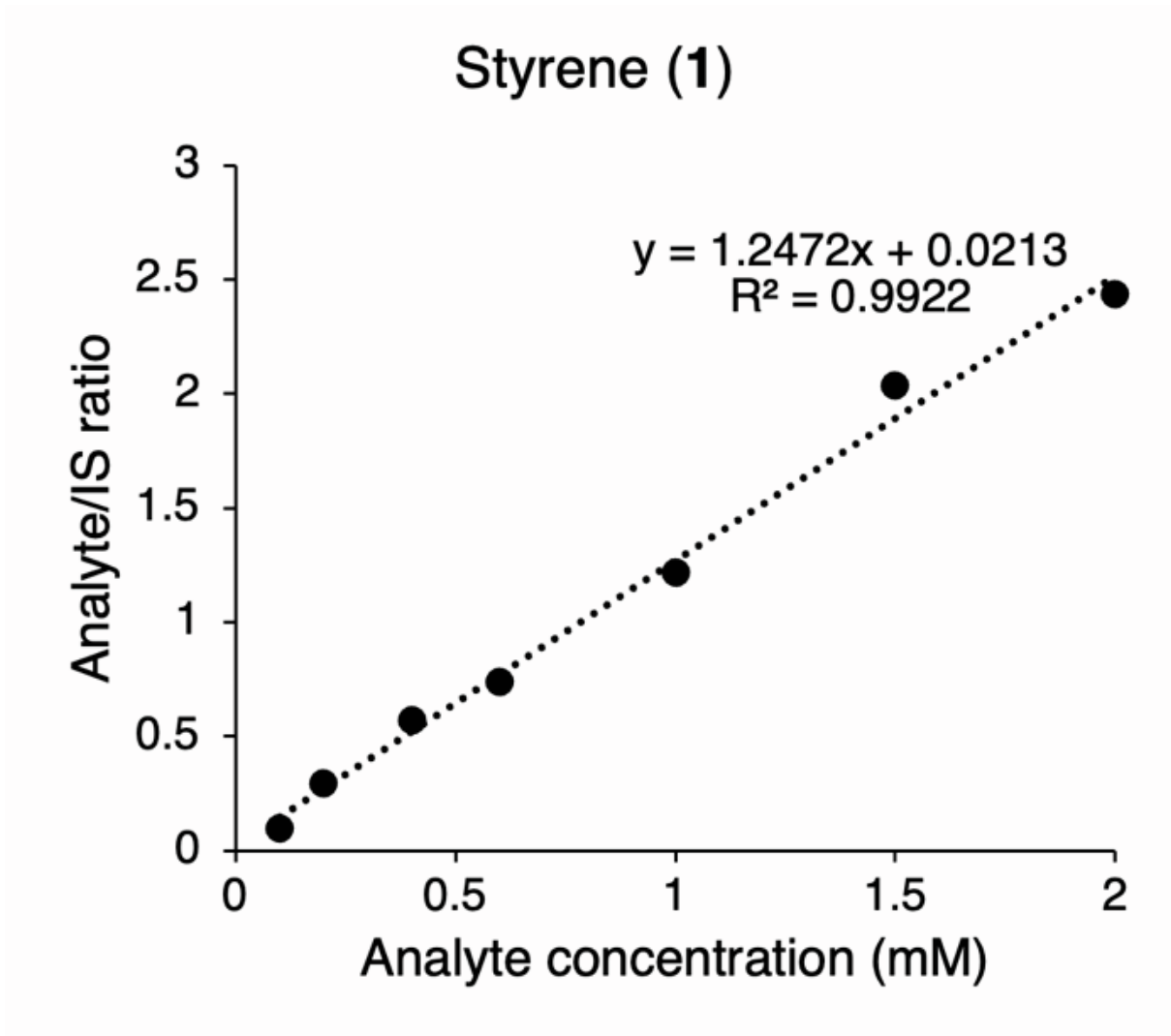

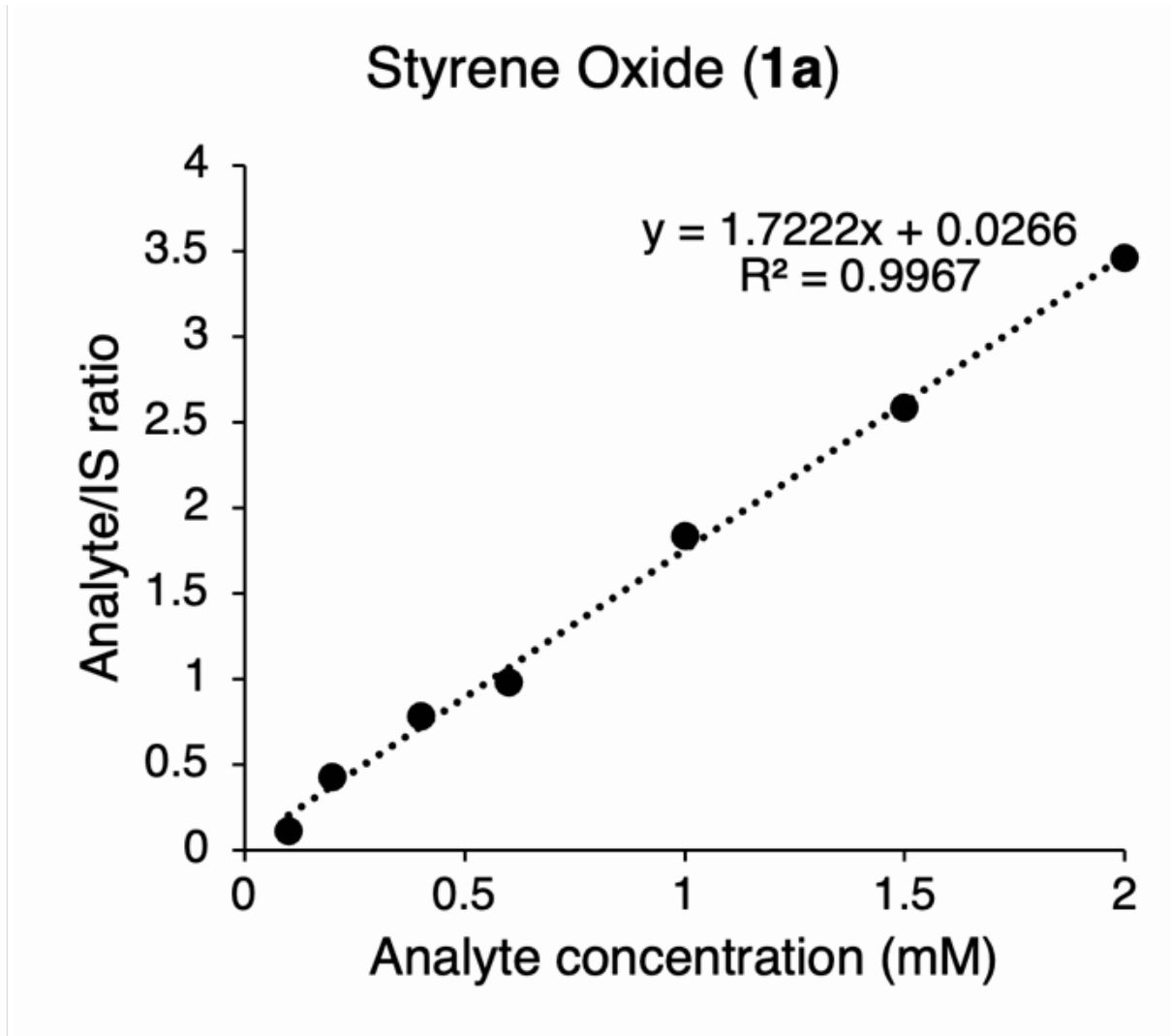

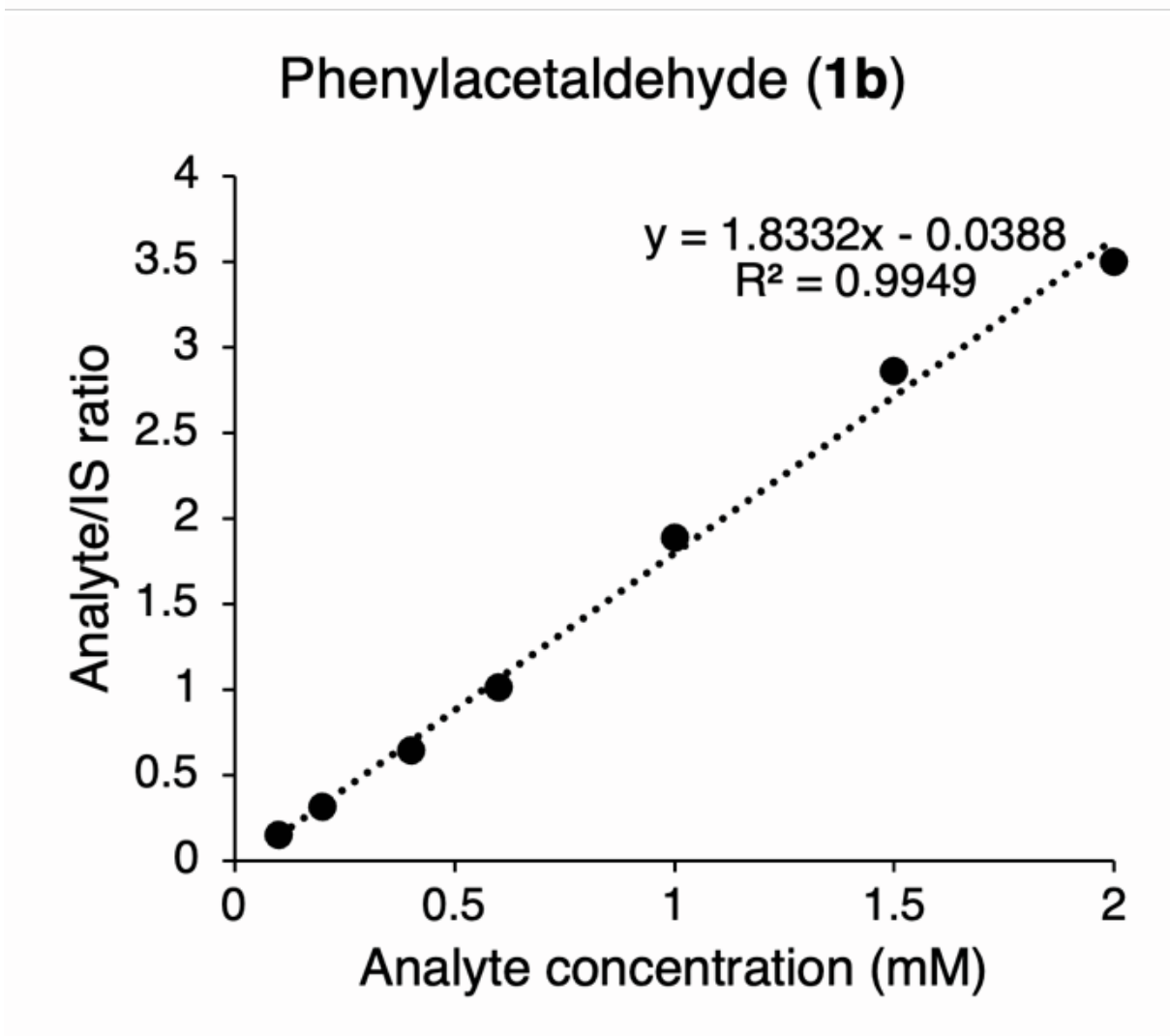

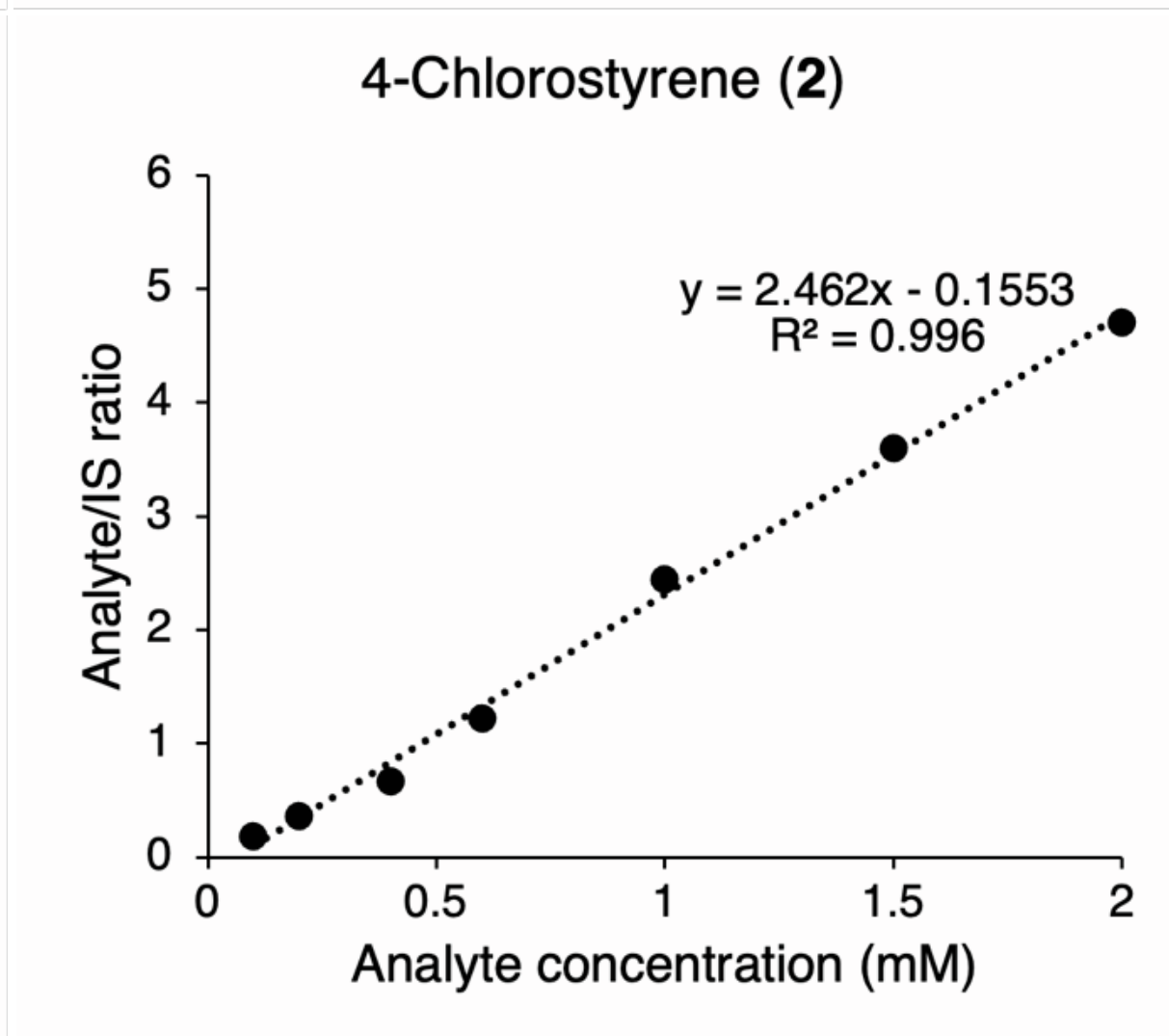

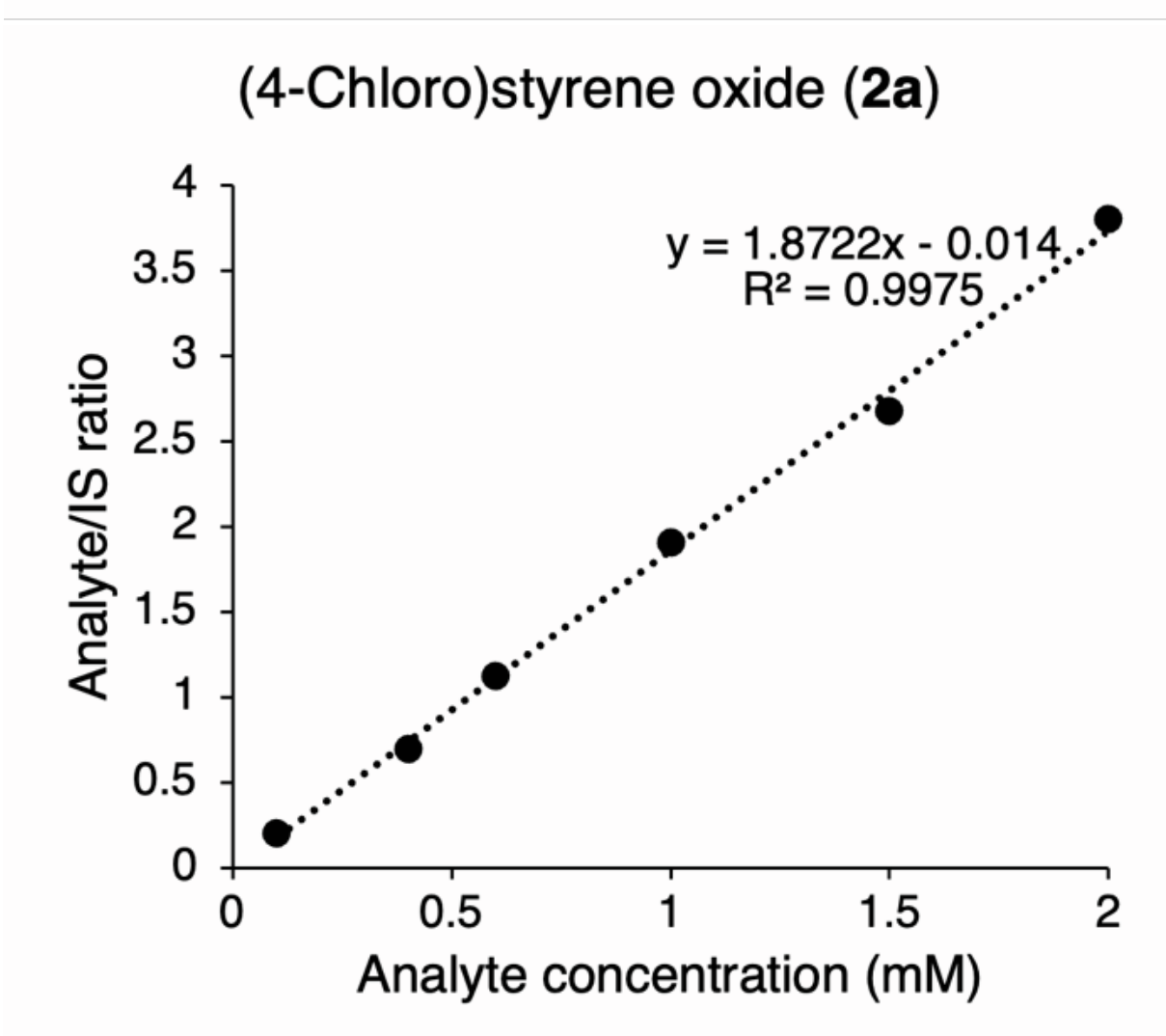

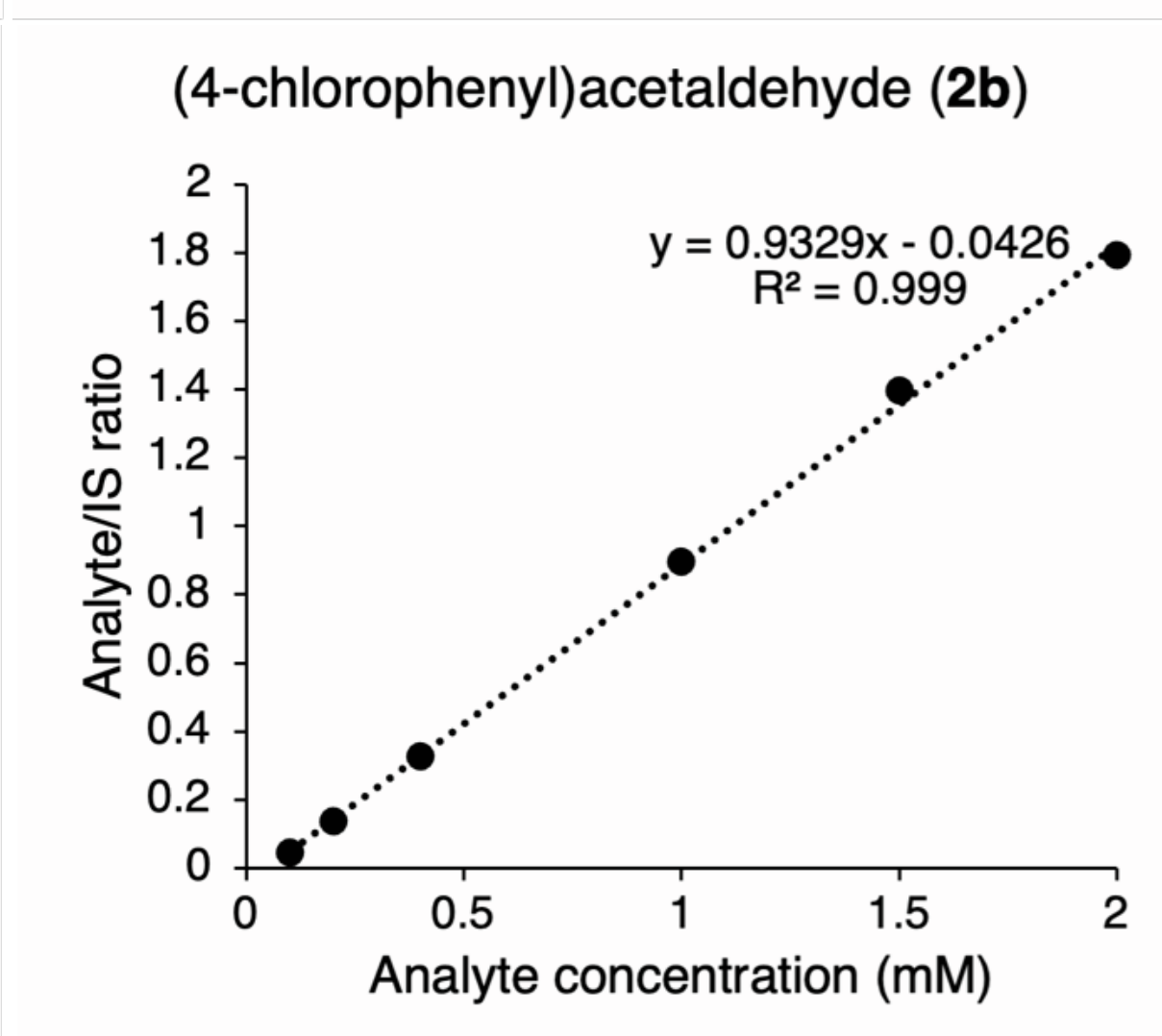

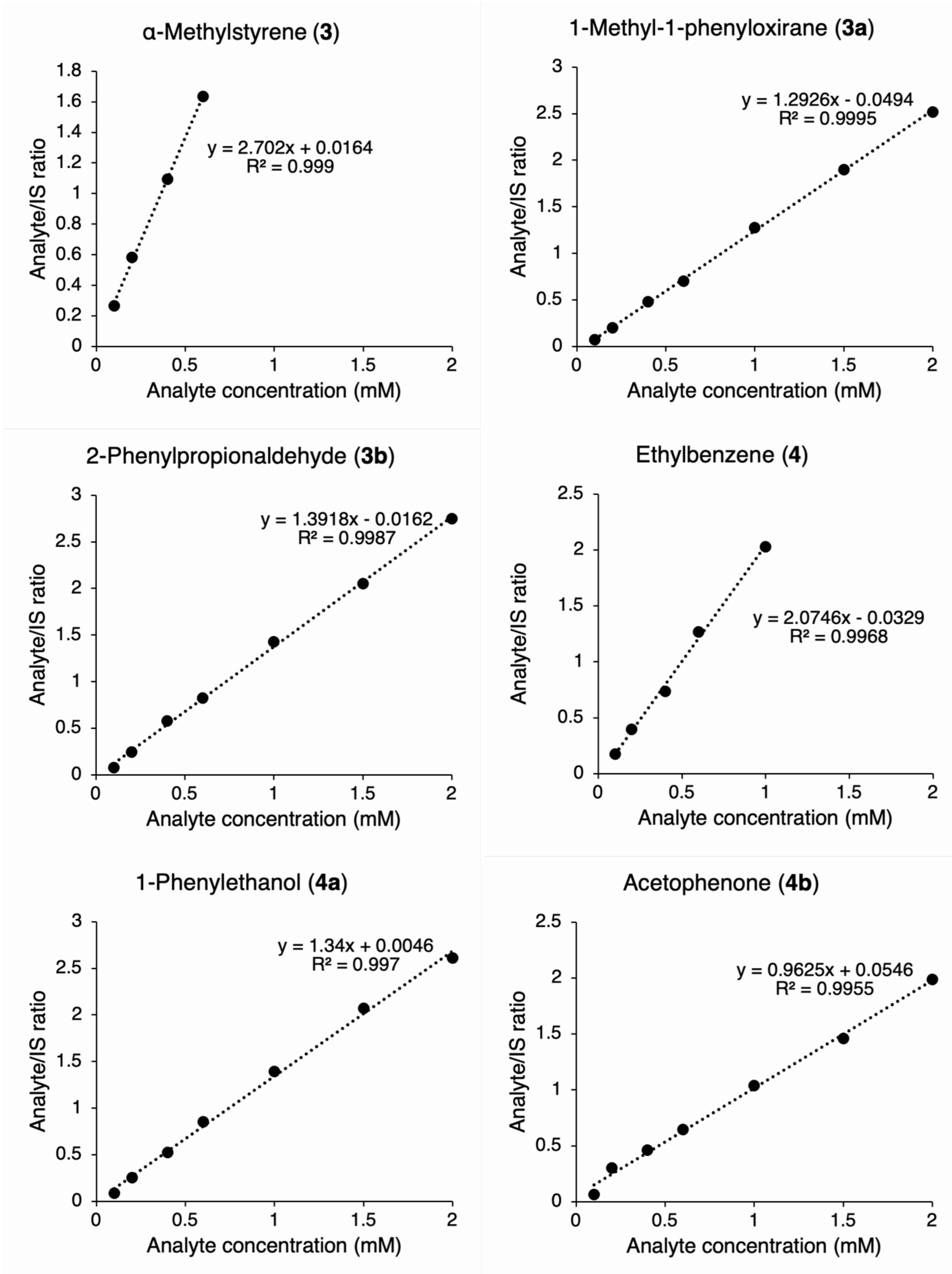
α-Methylstyrene (**3**)
y = 2.702x + 0.0164
R² = 0.999
1-Methyl-1-phenyloxirane (**3a**)
y = 1.2926x - 0.0494
R² = 0.9995
2-Phenylpropionaldehyde (**3b**)
y = 1.3918x - 0.0162
R² = 0.9987
Ethylbenzene (**4**)
y = 2.0746x - 0.0329
R² = 0.9968
1-Phenylethanol (**4a**)
y = 1.34x + 0.0046
R² = 0.997
Acetophenone (**4b**)
y = 0.9625x + 0.0546
R² = 0.9955
Analyte/IS ratio
Analyte concentration (mM)

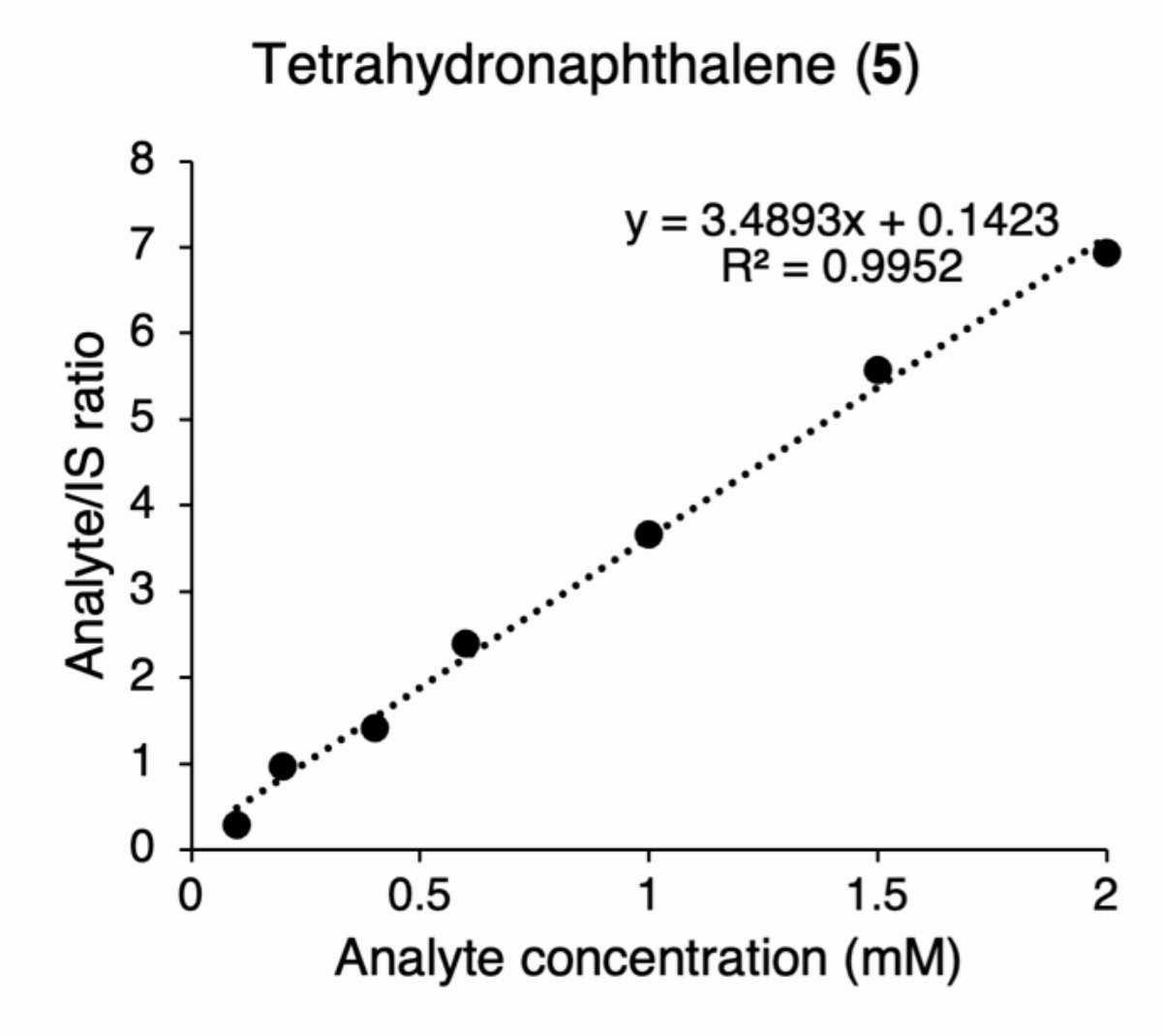
Tetrahydronaphthalene (5)
y = 3.4893x + 0.1423
R² = 0.9952
Analyte/IS ratio
Analyte concentration (mM)
0
1
2
3
4
5
6
7
8
0
0.5
1
1.5
2

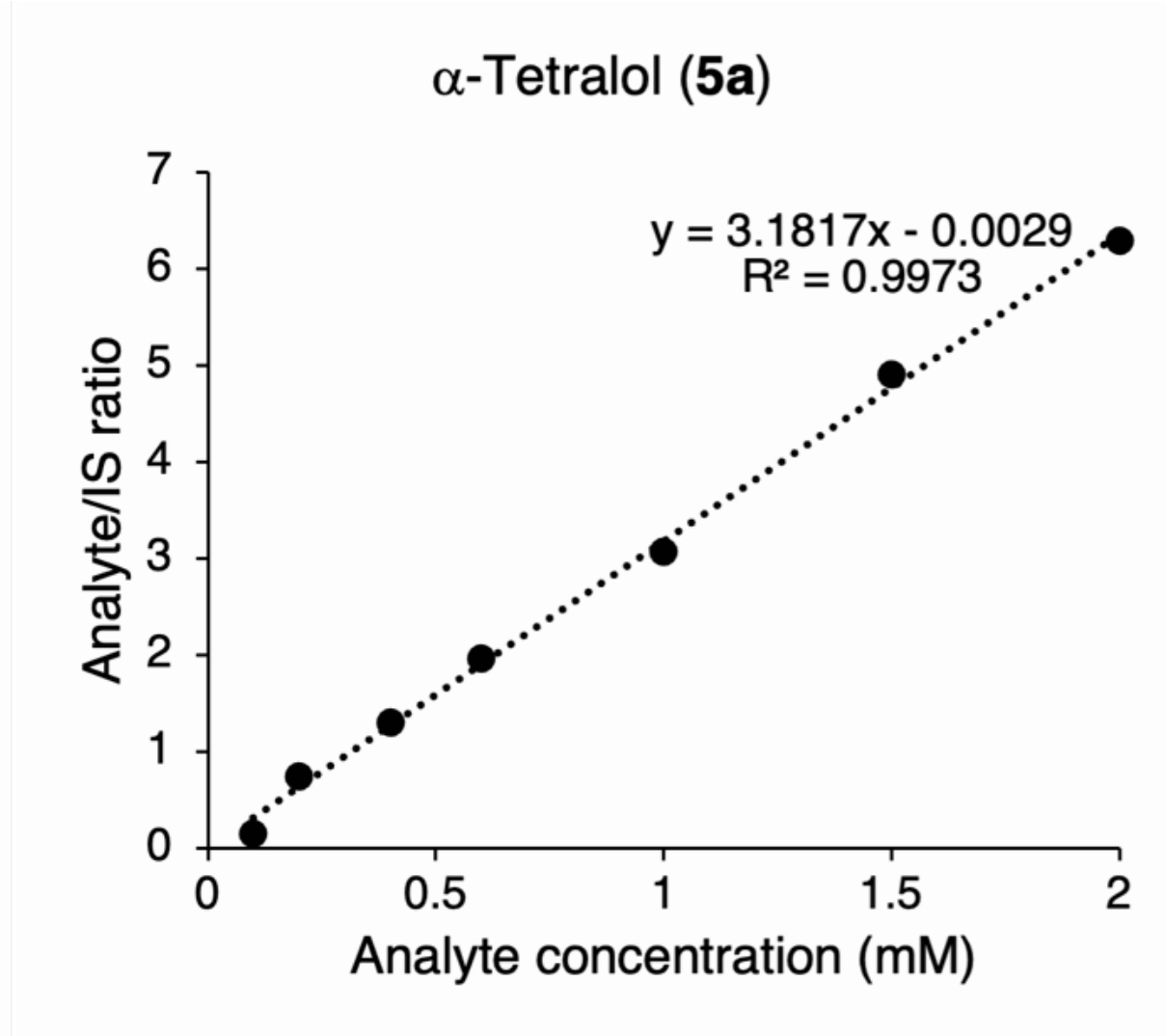
α-Tetralol (5a)
y = 3.1817x - 0.0029
R² = 0.9973
Analyte/IS ratio
Analyte concentration (mM)
0
1
2
3
4
5
6
7
0
0.5
1
1.5
2

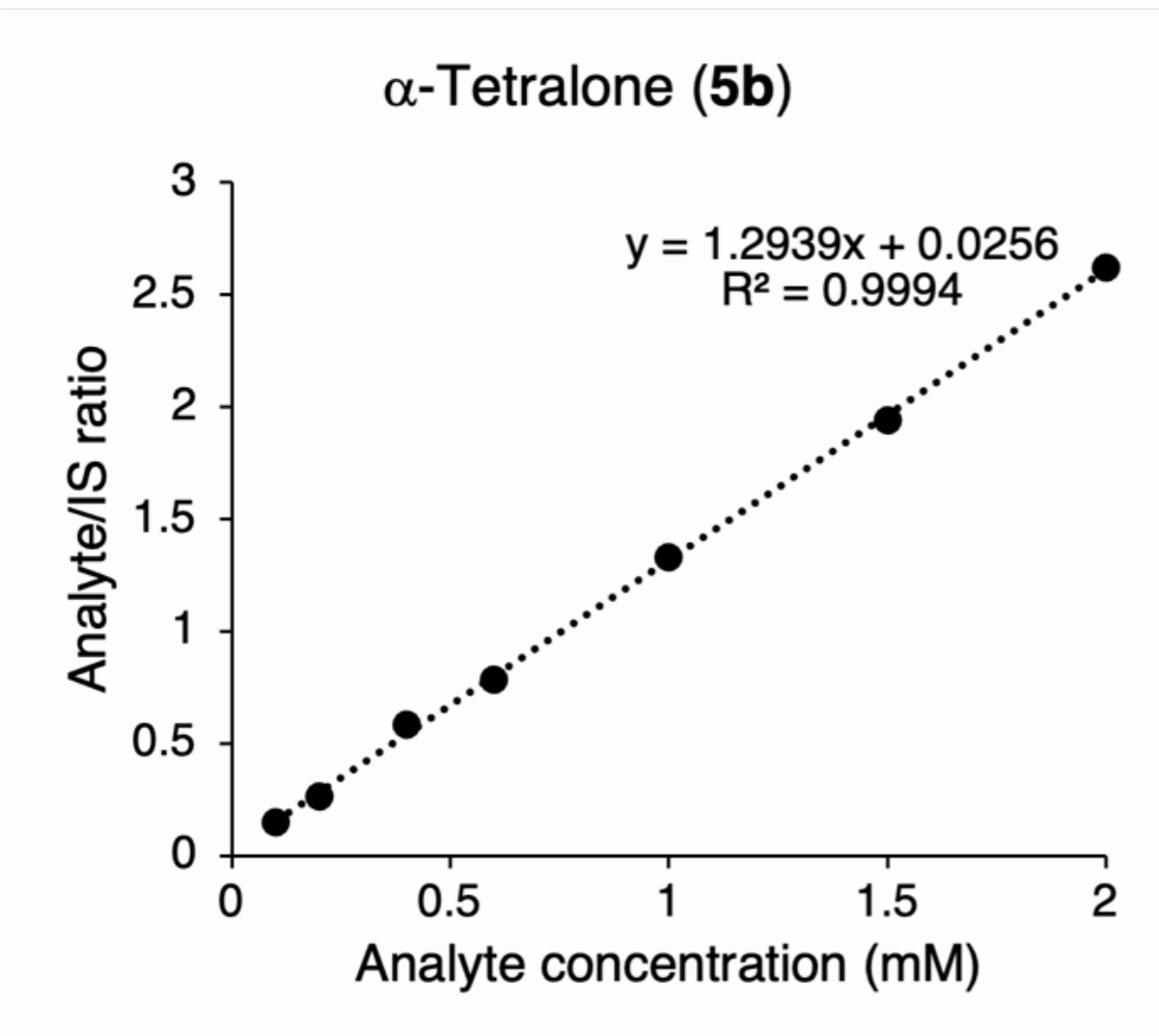
α-Tetralone (5b)
y = 1.2939x + 0.0256
R² = 0.9994
Analyte/IS ratio
Analyte concentration (mM)
0
0.5
1
1.5
2
2.5
3
0
0.5
1
1.5
2

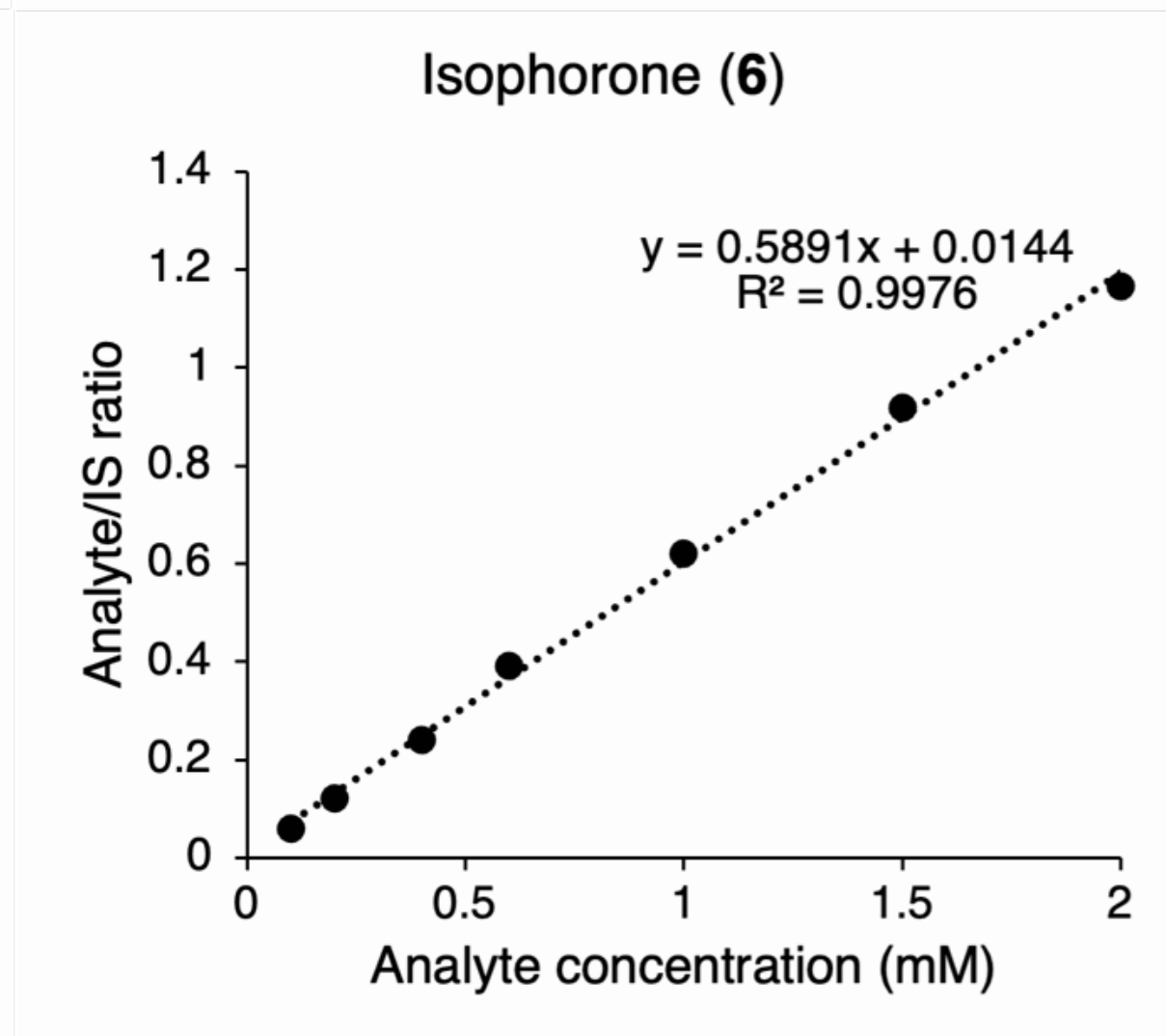
Isophorone (6)
y = 0.5891x + 0.0144
R² = 0.9976
Analyte/IS ratio
Analyte concentration (mM)
0
0.2
0.4
0.6
0.8
1
1.2
1.4
0
0.5
1
1.5
2

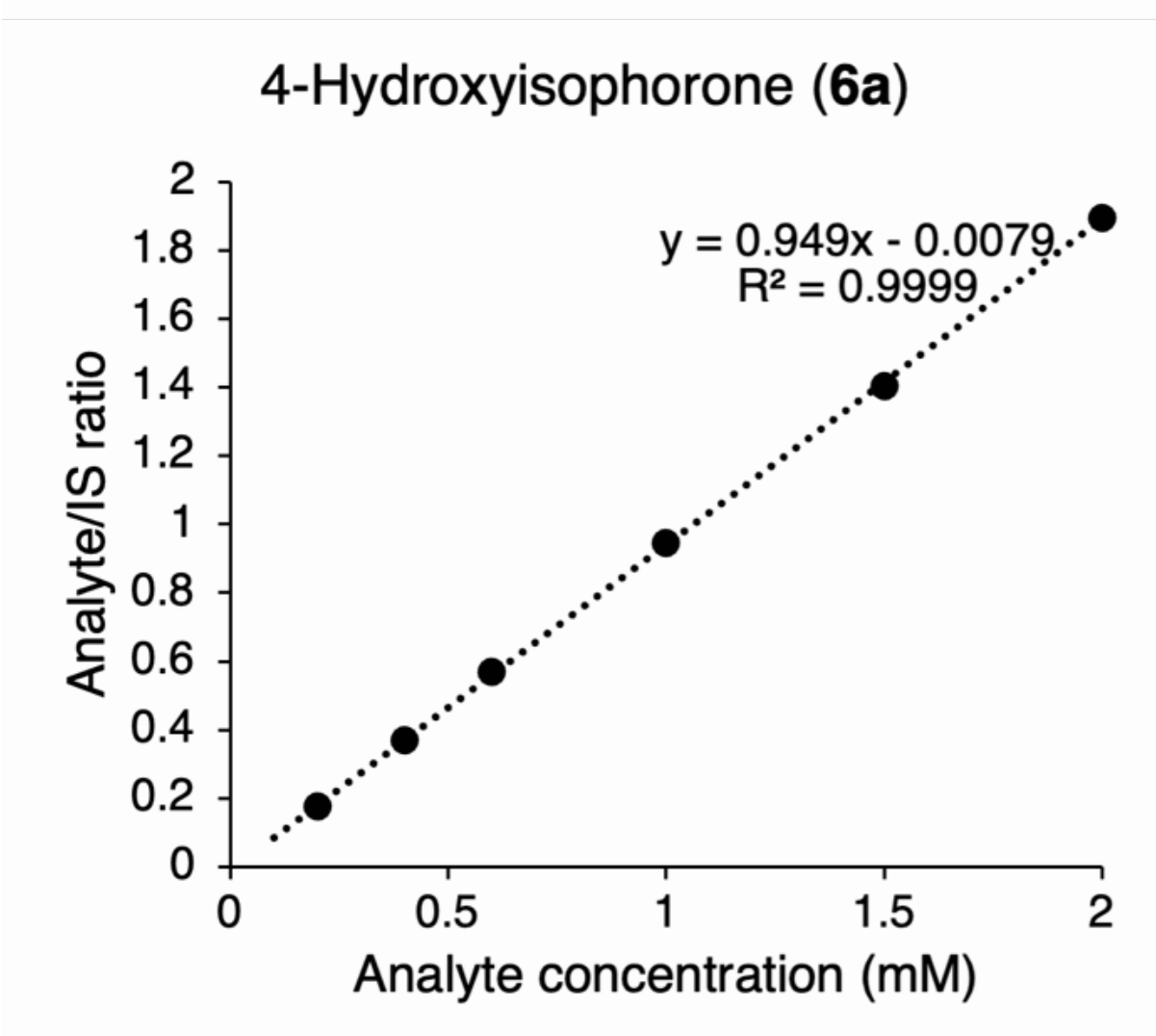
4-Hydroxyisophorone (6a)
y = 0.949x - 0.0079
R² = 0.9999
Analyte/IS ratio
Analyte concentration (mM)
0
0.2
0.4
0.6
0.8
1
1.2
1.4
1.6
1.8
2
0
0.5
1
1.5
2

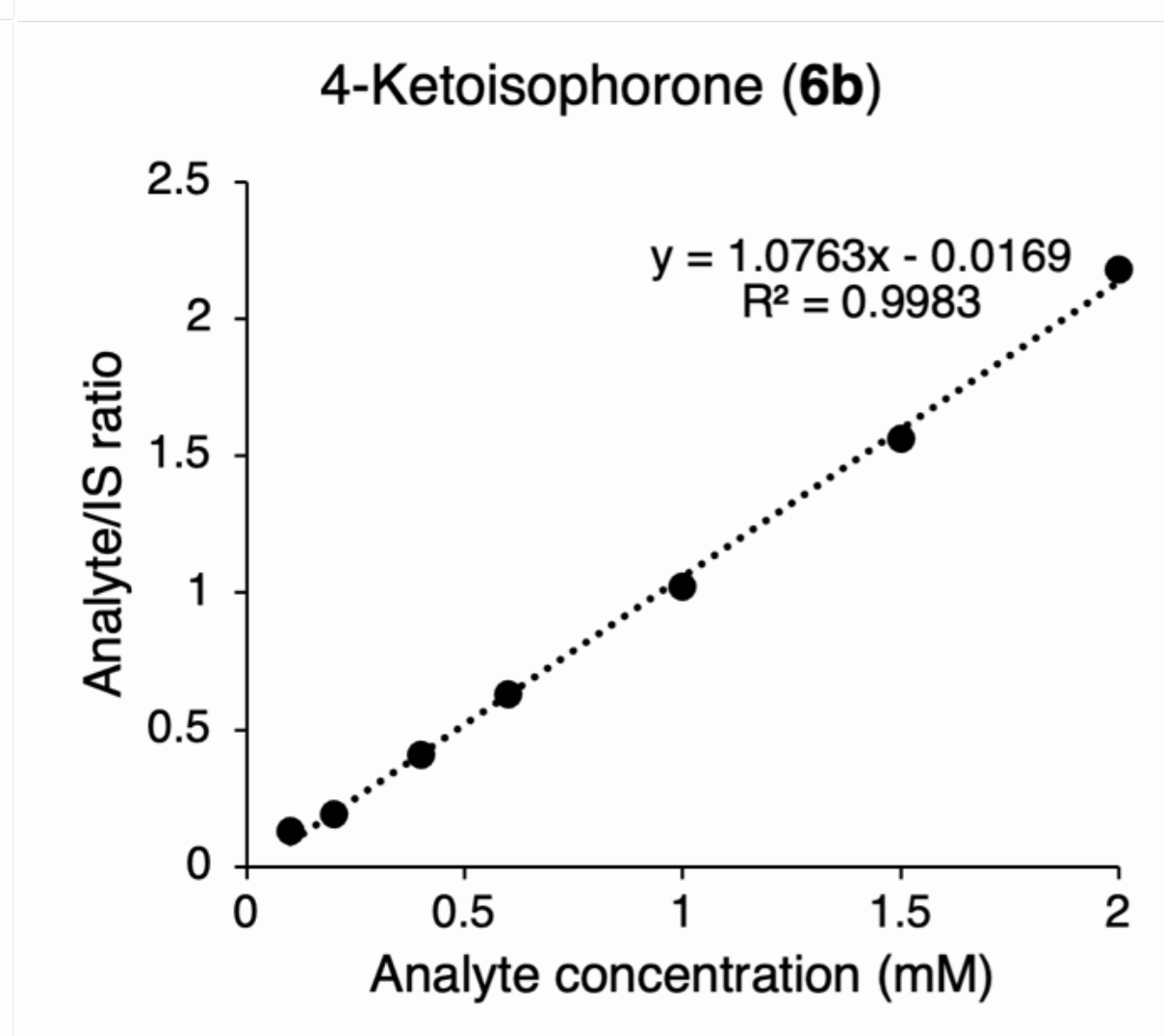
4-Ketoisophorone (6b)
y = 1.0763x - 0.0169
R² = 0.9983
Analyte/IS ratio
Analyte concentration (mM)
0
0.5
1
1.5
2
2.5
0
0.5
1
1.5
2

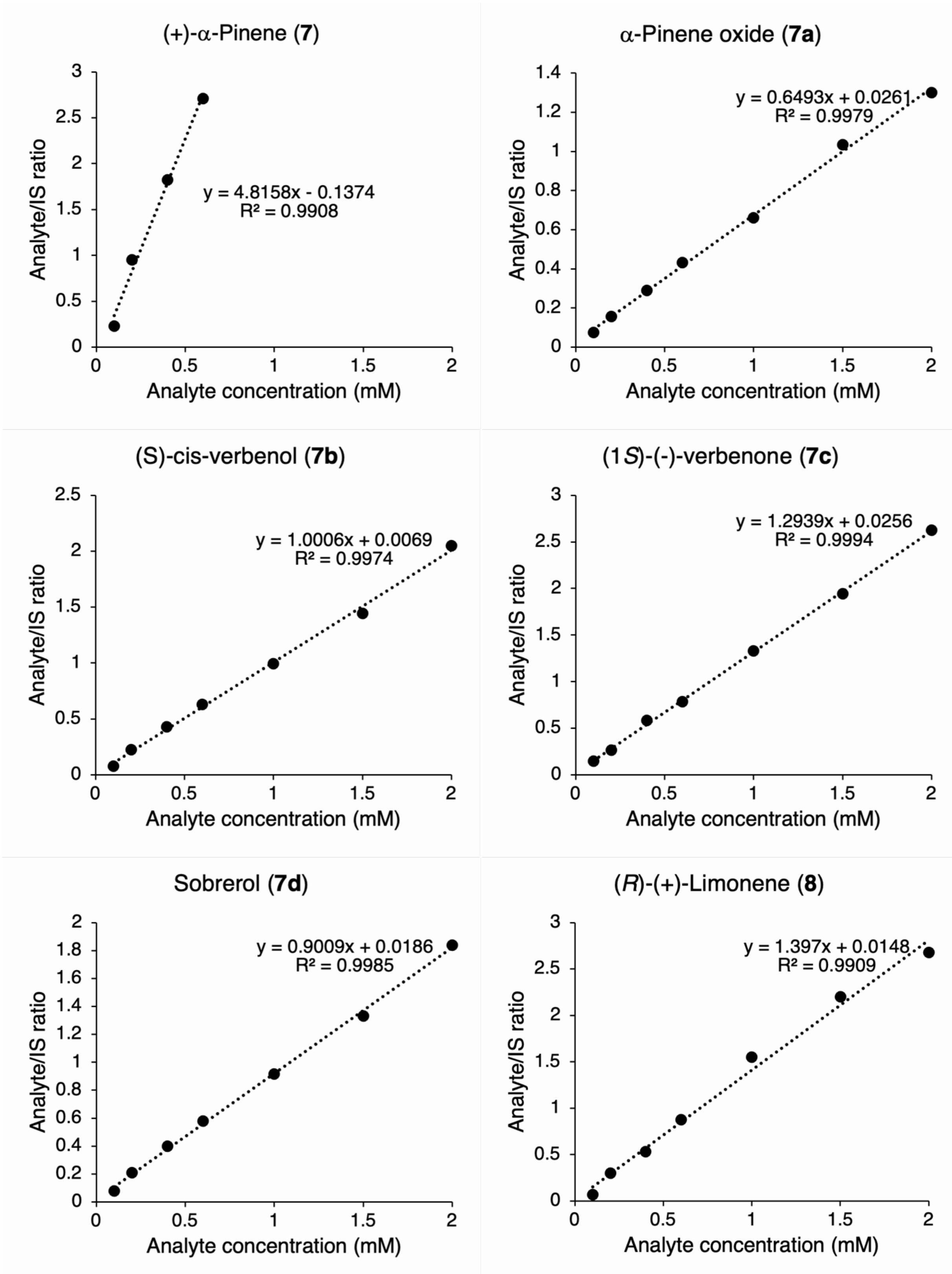
(+)-α-Pinene (7)
y = 4.8158x - 0.1374
R² = 0.9908
α-Pinene oxide (7a)
y = 0.6493x + 0.0261
R² = 0.9979
(S)-cis-verbenol (7b)
y = 1.0006x + 0.0069
R² = 0.9974
(1S)-(-)-verbenone (7c)
y = 1.2939x + 0.0256
R² = 0.9994
Sobrerol (7d)
y = 0.9009x + 0.0186
R² = 0.9985
(R)-(+)-Limonene (8)
y = 1.397x + 0.0148
R² = 0.9909
Analyte/IS ratio
Analyte concentration (mM)

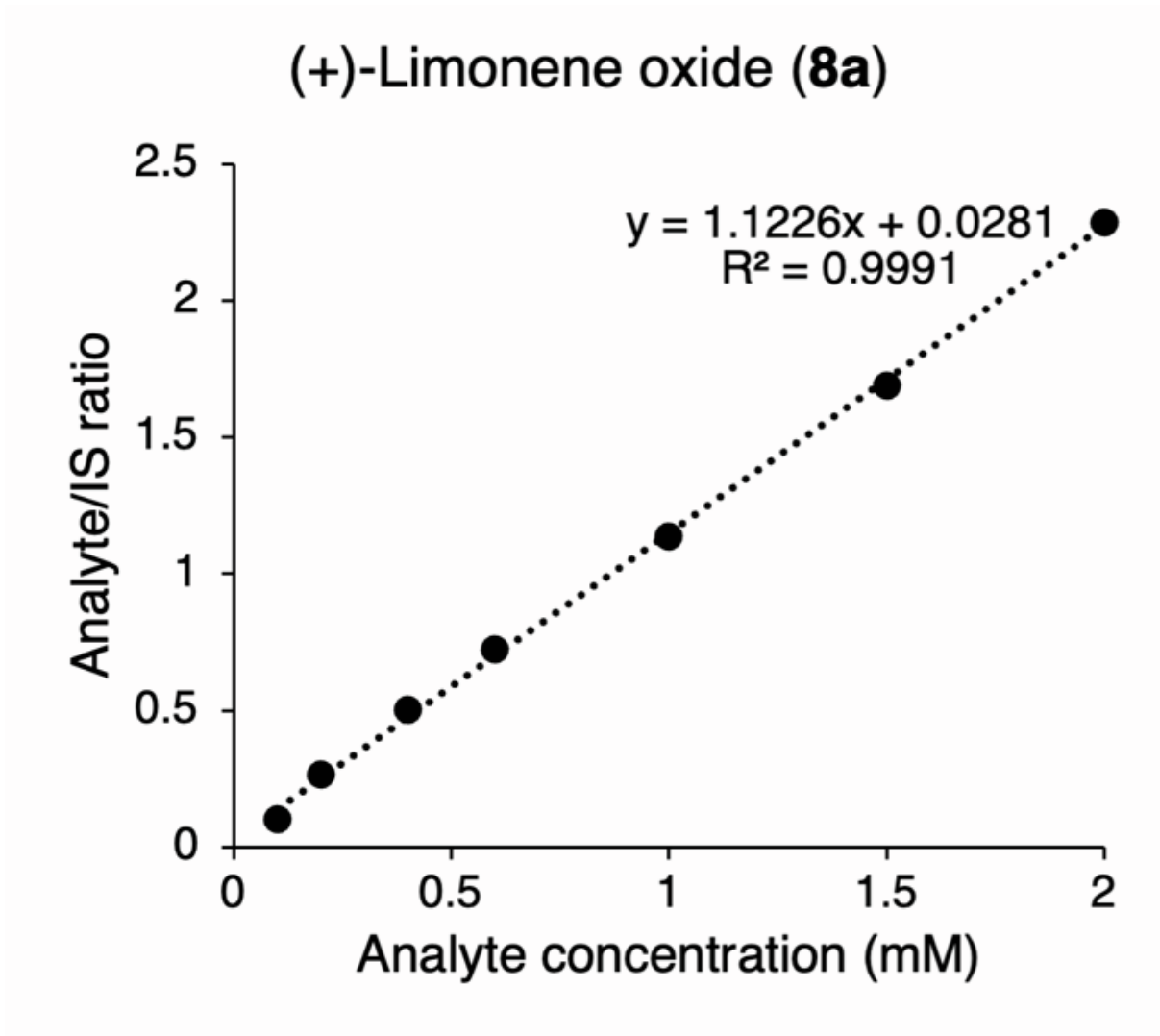

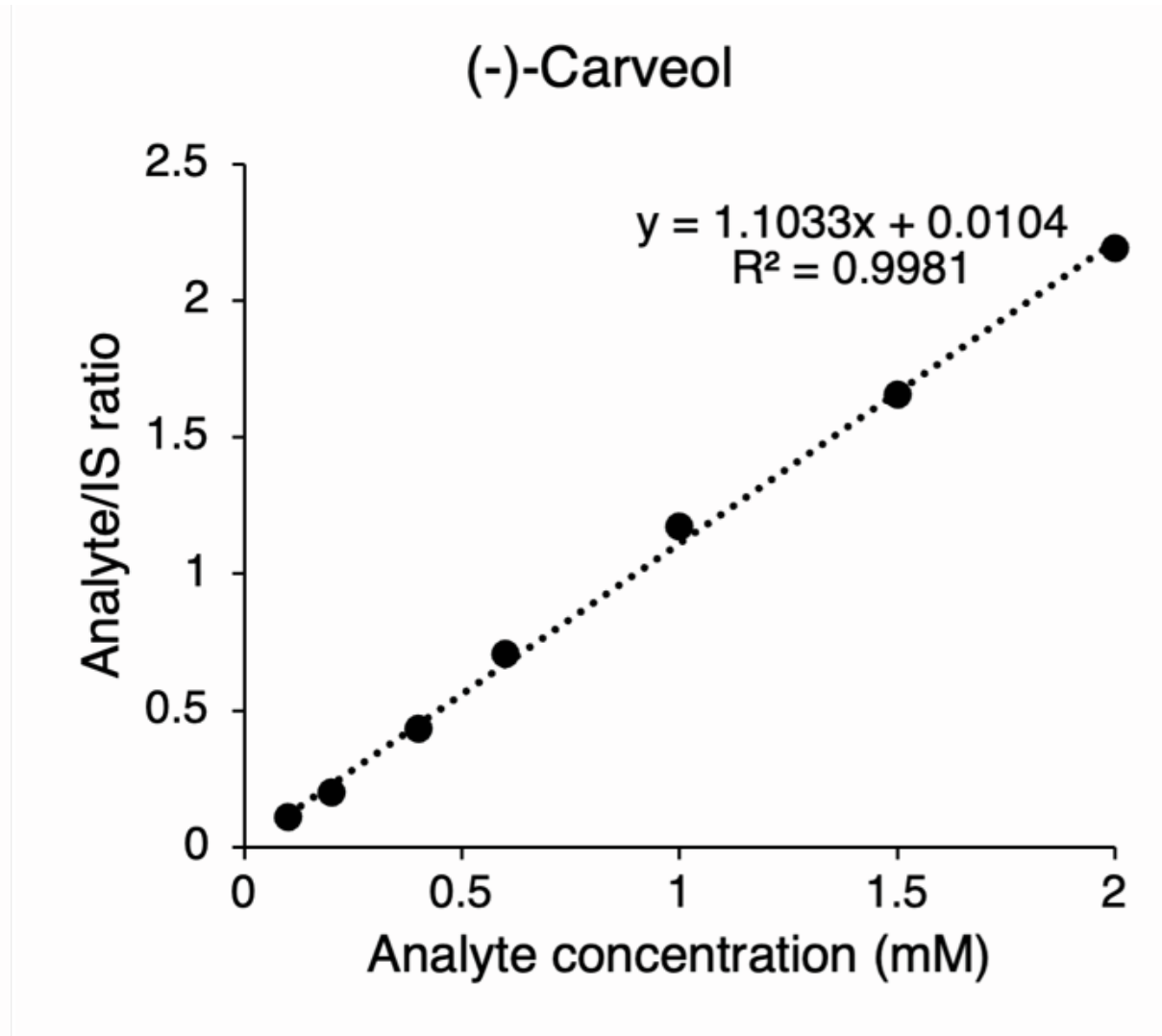

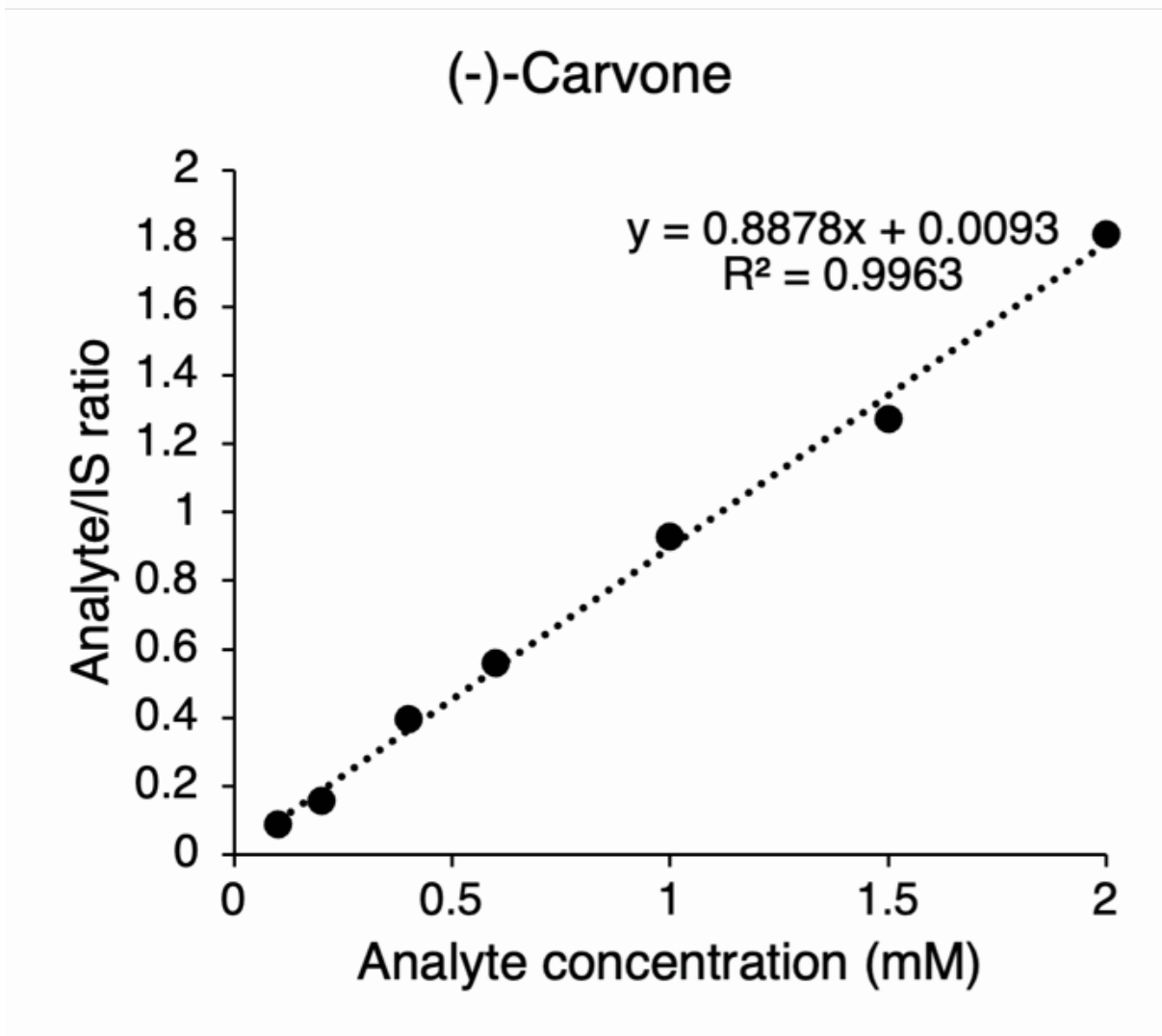

NOTE: α-Methylstyrene, ethylbenzene and pinene calibration curves are truncated due to saturation of MS detector at certain fragmentation masses. As the detected levels in the reaction mixtures are lower than this practical concentration limit, these curves were used for concentration calculations up to the maximum shown.

SUPPLEMENTAL METHODS S1: Synthetic procedures

Two authentic epoxide standards were synthesized for UPO product validation, (R)-limonene diepoxide and 1-methyl-1-phenyloxirane.

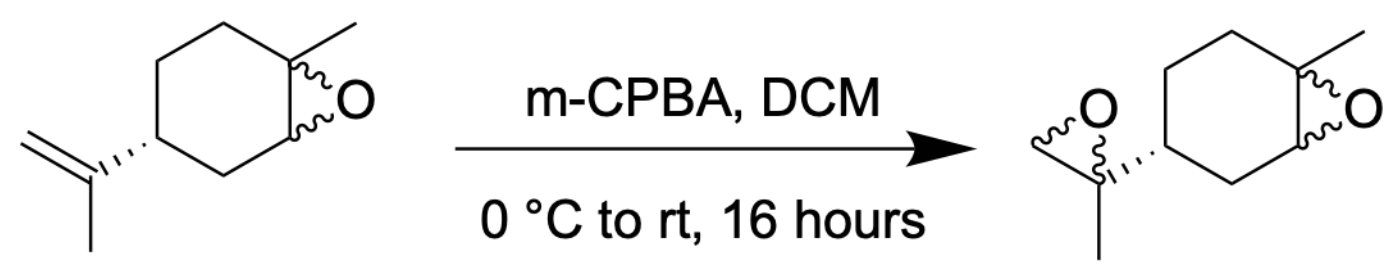

This reaction followed a previous report[4] with a change in scale and length of reaction. In a 50 mL round bottom flask, *m*-CPBA (0.85 g, 4.9 mmol, 1.5 eq) was suspended in 5 mL of CH_2Cl_2 at 0 °C with rapid stirring. (*R*)-Limonene oxide (mixture of cis and trans isomers) (0.5 g, 3.3 mmol, 1 eq) was diluted with 10 mL of CH_2Cl_2 and added to the mixture containing *m*-CPBA over 5 minutes. The reaction was allowed to slowly warm to room temperature, where it was vigorously stirred overnight. The organic white slurry was washed twice with 10 mL of an aq. solution of Na_2SO_3 (10 wt %), yielding a slightly cloudy biphasic mixture. The organic phase was washed twice with a saturated aq. solution of $NaHCO_3$, dried over $MgSO_4$ before removing the solvent in a rotary evaporator. Yield: 71 %.

^1H NMR matched reported spectra as a mixture of isomers.[2]

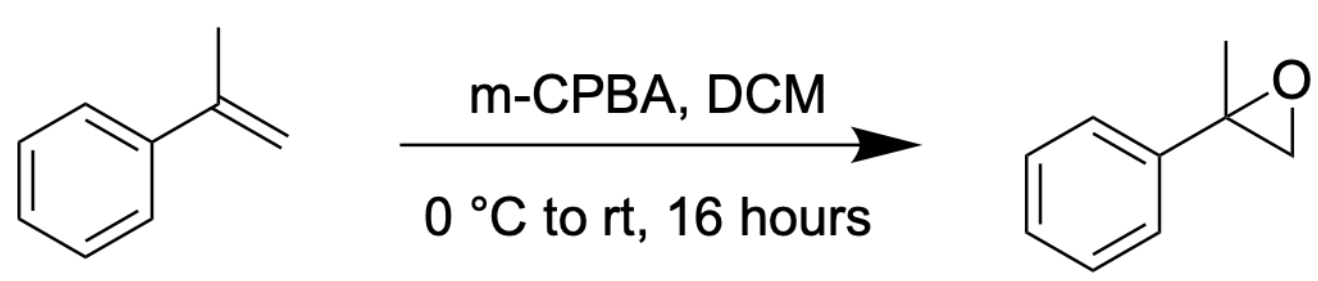

In a 50 mL round bottom flask, mCPBA (0.85 g, 4.9 mmol, 1.5 eq) was suspended in 5 mL of CH_2Cl_2 at 0 °C with rapid stirring. α-Methylstyrene (0.29 g, 3.3 mmol, 1 eq) was diluted with 10 mL of CH_2Cl_2 and added to the mixture containing m-CPBA over 5 minutes. The reaction was allowed to slowly warm to room temperature, where it was vigorously stirred overnight. The organic white slurry was washed twice with 10 mL of an aq. solution of Na_2SO_3 (10 wt%), yielding a clear

biphasic mixture. The organic phase was washed twice with a saturated aq. solution of $NaHCO_3$, dried over $MgSO_4$ before removing the solvent in a rotary evaporator. Yield: 82 %.

^1H NMR matched reported spectra.[3]

SI REFERENCES

1. Sievers, F., and Higgins, D.G. (2018). Clustal Omega for making accurate alignments of many protein sequences. Protein Science *27*, 135–145. https://doi.org/10.1002/pro.3290.

2. Schutz, L., Kazemi, F., Mackenzie, E., Bergeron, J., Gagnon, E., and Claverie, J.P. (2021). Trans‐limonene dioxide, a promising bio‐based epoxy monomer. J. Polym. Sci. *59*, 321–328. https://doi.org/10.1002/pol.20200822.

3. Daniel, P.E., Weber, A.E., and Malcolmson, S.J. (2017). Umpolung Synthesis of 1,3-Amino Alcohols: Stereoselective Addition of 2-Azaallyl Anions to Epoxides. Org. Lett. *19*, 3490–3493. https://doi.org/10.1021/acs.orglett.7b01471.
